# Zebrafish *cobll1a* regulates lipid homeostasis via the RA signaling pathway

**DOI:** 10.3389/fcell.2024.1381362

**Published:** 2024-04-18

**Authors:** Ting Zeng, Jinrui Lv, Jiaxin Liang, Binling Xie, Ling Liu, Yuanyuan Tan, Junwei Zhu, Jifan Jiang, Huaping Xie

**Affiliations:** ^1^ Hunan International Joint Laboratory of Animal Intestinal Ecology and Health, Laboratory of Animal Nutrition and Human Health, College of Life Sciences, Hunan Normal University, Changsha, Hunan, China; ^2^ Hunan Provincial Key Laboratory of Animal Intestinal Function and Regulation, Changsha, Hunan, China

**Keywords:** Zebrafish, liver, retinol, retinoic acid signaling pathway, NAFLD, apolipoprotein

## Abstract

**Background:**

The *COBLL1* gene has been implicated in human central obesity, fasting insulin levels, type 2 diabetes, and blood lipid profiles. However, its molecular mechanisms remain largely unexplored.

**Methods:**

In this study, we established *cobll1a* mutant lines using the CRISPR/Cas9-mediated gene knockout technique. To further dissect the molecular underpinnings of *cobll1a* during early development, transcriptome sequencing and bioinformatics analysis was employed.

**Results:**

Our study showed that compared to the control, *cobll1a* ^−/−^ zebrafish embryos exhibited impaired development of digestive organs, including the liver, intestine, and pancreas, at 4 days post-fertilization (dpf). Transcriptome sequencing and bioinformatics analysis results showed that in *cobll1a* knockout group, the expression level of genes in the Retinoic Acid (RA) signaling pathway was affected, and the expression level of lipid metabolism-related genes (*fasn*, *scd*, *elovl2*, *elovl6*, *dgat1a*, *srebf1* and *srebf2*) were significantly changed (*p* < 0.01), leading to increased lipid synthesis and decreased lipid catabolism. The expression level of apolipoprotein genes (*apoa1a*, *apoa1b*, *apoa2*, *apoa4a*, *apoa4b*, and *apoea*) genes were downregulated.

**Conclusion:**

Our study suggest that the loss of *cobll1a* resulted in disrupted RA metabolism, reduced lipoprotein expression, and abnormal lipid transport, therefore contributing to lipid accumulation and deleterious effects on early liver development.

## 1 Introduction

Vitamin A, known as retinol, is essential for normal physiological functions in living organisms. Retinol’s metabolite, retinoic acid (RA), is pivotal in cellular proliferation and differentiation (Gudas and Wagner, 2011), embryonic growth (Zile, 2001), organogenesis (Stafford et al., 2006), visual transduction (Zhong et al., 2012), immune regulation (Bono et al., 2016), and glucose and lipid metabolism (Tanumihardjo et al., 2016). Retinol Ester (RE) in food is hydrolyzed to retinol by retinol ester hydrolase (REH) (Boily et al., 2003). Afterward, retinol forms a complex with retinol binding protein 4 (RBP4) and plasma transthyretin (TTR) secreted by the liver before being transported to peripheral tissues (Ronne et al., 1983). This RBP4-TTR-retinol complex binds to the signaling receptor and transporter of retinol, STRA6 (STRA6), and undergoes two dehydrogenation reactions to produce RA (Theodosiou et al., 2010; Sun and Kawaguchi, 2011). RA is subsequently rapidly degraded by P450 family enzymes (Pennimpede et al., 2010). Vitamin A deficiency (VAD) is associated with metabolic syndrome (MetS) with liver issues like steatosis seen in rats lacking Vitamin A (Baybutt et al., 2000). Approximately 80% of retinol is stored as RE in the lipid droplets of quiescent hepatic stellate cells (HSCs) (Haaker et al., 2020). Liver injury leads to fibrosis of hematopoietic stem cells, which ultimately leads to retinol deficiency (Saeed et al., 2017). It is reported that decreased vitamin A intake result in nonalcoholic fatty liver disease (NAFLD) (Musso et al., 2007), and obese patients with NAFLD display reduced serum retinol (Villaça Chaves et al., 2008). NAFLD is a hepatic manifestation of MetS characterized by hepatic steatosis, fibrogenesis, and inflammation (Friedman et al., 2018).

The *COBLL1* gene, originally isolated from a human brain cDNA library, is highly expressed in a variety of tissues including the liver, lung, kidney, pancreas, ovary, spinal cord, and brain (Nagase et al., 1999). It is a negative regulator of tumor cell apoptosis and is implicated in the progress of various cancers (Han et al., 2017; Takayama et al., 2018), as well as in insulin resistance-related metabolic diseases (Mancina et al., 2013). Genome-wide association studies have revealed the localization of the human *COBLL1* gene in genomic regions associated with metabolism, including high-density cholesterol, triglyceride metabolism, insulin levels, type 2 diabetes, and other metabolic disorders (Manning et al., 2012; Albrechtsen et al., 2013; Desmarchelier et al., 2014). Furthermore, the SNP rs7607980 in the *COBLL1* gene affects insulin resistance (Kraja et al., 2014), and its polymorphic form has been identified in patients with MetS (Albrechtsen et al., 2013). Loss of *COBLL1* results in excessive lipid accumulation and increased lipolysis in preadipocytes (Simpson-Golabi-Behmel syndrome), suggesting that COBLL1 may have a crucial role in lipid metabolism (Chen et al., 2020). In zebrafish, two *COBLL1* homologs have been identified: *cobll1a* on chromosome 9 and *cobll1b* on chromosome 6. Both homologs possessed a pair of KRAP domains, which are pivotal for protein-protein interactions and subcellular localization (Schwintzer et al., 2011). It is reported that *cobll1b* plays an important role in hematopoiesis during early embryonic development (Kim et al., 2017). Utilizing CRISPR/Cas9 gene editing technology, we constructed zebrafish *cobll1a* gene knockout lines to ascertain the role of this gene in embryonic development. Our study found that the digestive organs, such as the liver, intestine, and pancreas developed abnormally in 4 dpf *cobll1a* ^−/−^ embryos, compared to the control. To investigate the molecular mechanism underlying *cobll1a* gene regulation of digestive organ development, transcriptome sequencing and bioinformatics analysis were conducted. We found a significant alteration in the expression levels of genes related to lipid metabolism in the *cobll1a* gene knockout. Loss of *cobll1a* led to abnormal RA metabolism, increased lipid synthesis, decreased lipolysis metabolism, disrupted lipoprotein metabolism, abnormal lipid transport, and subsequent lipid accumulation. In conclusion, the *cobll1a* gene is critical for the RA signaling pathway and lipid metabolism in zebrafish.

## 2 Materials and methods

### 2.1 Zebrafish breeding

Tuebingen (TU) zebrafish were bred and maintained in our laboratory under the following conditions: a water temperature of 28°C, pH 6.5–7.5, salinity of 450–500 μs/cm, and a 14 h/10 h light and dark cycle. Fish were crossed weekly, the embryos were cultivated at 28.5°C in E3 solution.

### 2.2 Establishment of zebrafish *cobll1a* mutant lines

Mutant lines of zebrafish *cobll1a* were established using CRISPR/Cas9 gene editing technology. The complete gene and amino acid sequence of zebrafish *cobll1a* were obtained from the NCBI database, and the corresponding genome sequence was accessed from UCSC. The target sequence of *cobll1a* was identified via the CRISPOR website, and the target sites were localized in exon 4. Two target sequences were selected: sequence 1 (sgRNA-F1) is 5′-ACC​AGT​TAT​GGA​TGT​TC-3′, and sequence 2 (sgRNA-F2) is 5′-TGA​TCG​GCT​CTC​TCG​AAT-3’. The core sequence of T7 RNA polymerase promoter was added to the 5′ end of the target sequence to generate forward primers. The PCR amplification, using forward primers (sgRNA-F1/2) and reverse primers (sgRNA-R), the purified PCR product as a template, sgRNAs were synthesized *in vitro* with the T7 *in vitro* transcription kit (ThermoFisher). Purified sgRNAs were recovered with the RNA purification kit (Qiagen) and mixed with Cas9 protein (ThermoFisher) for injection. Zebrafish embryos were injected at the one-cell stage and then incubated at 28.5°C. Genomic DNA was extracted from the F0 embryos and tested for *cobll1a* mutation using primers 5′-TGC​ATA​TAC​TGT​ATG​TGG​GAC​A-3′ and 5′-TCT​TGT​TGT​CGT​CAC​TTC​CT-3’. DNA showing the deletions or insertions was sequenced and the genomic DNA of F1 and F2 generations were amplified using the following primers (F) 5′-TGC​ATA​TAC​TGT​ATG​TGG​GAC​A-3′ and (R) 5′-TGA​CAA​AAC​TGA​CCA​CT-3’.

At 36 hours post-fertilization (hpf), the effciency of the sgRNAs was validated by randomly selecting injected and wild-type embryos. The PCR product of the control is 775 bp, with the two target sites being 134 bp apart. If both target sites work, a deletion or an insertion sequence would be generated, in comparison to the control. After validating the two target sites successfully work, the remaining embryos were raised to 45 dpf. Caudal fin clipping was performed individually, and genomic DNA was extracted for genotyping. Fish with missing or inserting DNA sequences were raised to adulthood (F0) and crossbred with wild-type to obtain F1 generation. Genotypes of F1 mutants were determined, and DNA fragments less than 775 bp were recovered and sequenced. Mutant lines resulting in protein-coding frame shift were selected for further studies.

### 2.3 Whole-mount *in situ* hybridization (WISH)

Zebrafish embryos at the desired stages were fixed overnight in 4% paraformaldehyde (PFA) at 4°C. They were then rinsed twice with PBST, treated with a 10 mg/mL protease K solution, refixed with 4% PFA at room temperature for 20 min, and rinsed twice again with PBST. They were then incubated with DIG-labeled antisense RNA probe overnight at 68°C. On the following day, the embryos were washed with 50% formamide/2xSSCT, 2xSSCT and 0.2xSSCT solutions and incubated with anti-DIG antibodies overnight. After washing three times with MABT solution, with a 25 min interval, the staining reaction was performed with BCIP-NBT solution and imaged using a Leica stereo microscope. Antisense RNA probes labeled with digoxin-UTP for *cobll1a*, *fabp10a*, *insulin*, *trypsin*, *fabp2*, *rdh10*, *radh1a2*, *cyp26a1*, *rbp4*, and *fasn* genes were synthesized using the T7 *in vitro* transcription kit (Thermo Fisher). The primer sequences for these genes were listed in Supplementary Table S1.

### 2.4 Quantitative real-time polymerase chain reaction (qRT-PCR)

Fifty 52 hpf control and *cobll1a* ^−/−^ embryos were collected in triplicate, quickly frozen in liquid nitrogen and stored at −80°C. Total RNA was extracted from the embryos using TRIZOL (Takara) reagent under manufacturer’s instructions. 1μg of RNA was reverse transcribed into cDNA using reverse transcriptase (TaKaRa) and oligonucleotide primers. Expression level of related genes was measured by quantitative PCR using 2×SYBR Green Master qPCR Mix (Vazyme). The qPCR primers used for this study are listed in Supplementary Table S2. Relative expression level of the tested mRNAs were determined using *18s* as an internal reference and the comparative Ct (2−^△△^Ct) method (*p* < 0.05).

### 2.5 RNA sequencing and differentially expressed genes analysis

Fifty 52 hpf control and *cobll1a* ^−/−^ embryos were collected in triplicate and were subjected to RNA-seq and data analysis by Shanghai Ouyi Biotechnology Co, LTD. (Shanghai, China). Total RNA was extracted using the mirVana miRNA Separation Kit (Ambion), and the integrity of RNA was evaluated using the Agilent 2,100 bioanalyzer (Agilent Technologies, Santa Clara, CA, United States). Following manufacturer’s instructions, cDNA libraries were prepared and sequenced on the Illumina platform (HiSeqTM 2,500 or Illumina HiSeq X Ten) to produce paired-end reads of 125 bp/150 bp. The raw data were processed by Trimmomatic, and the cleaned read segments were mapped to the GRCz11 reference genome using Hisat2. FPKM was calculated using the output file from feature Counts. Genes with FPKM>1 were considered as expressed, and the DESeq2 (v 1.40.2) algorithm was employed for differential gene expression analysis. Differentially expressed genes (DEGs) were filtered with *p* < 0.05 and |log2FoldChange|>1 as thresholds. Volcano plots and heat maps were generated to display the distribution of overlapping DEGs between the knockout and the control groups. The R cluster Profiler package (v 4.8.3) was used for GO and KEGG enrichment analysis of DEGs. The enrichment results were visualized using the R package ggplot2 (v 3.4.3) and the online analysis website Sangerbox3.0 (http://www.sangerbox.com/). Gene Set Enrichment Analysis (GSEA) was also performed on the list of genes whose fold changes were detected in the experiment. The enrichment of up-regulated or down-regulated gene sets in the KEGG pathway database was calculated, excluding gene sets with fewer than 10 genes or more than 500 genes. The T-statistical average of genes in each KEGG pathway was calculated using 1,000 permutation tests. Pathways with a normalized enrichment score (NES) > 0 were considered up-regulated, and those with NES<0 were considered down-regulated. Key genes in each pathway were annotated using the biomaRt software package and the GSEA enrichment results were visualized through the function implementation package “GseaVis”.

### 2.6 Oil red O staining

Fixed zebrafish embryos were initially rinsed with phosphate-buffered saline (PBS) for 5 min, followed by permeabilization using sequentially 60% and 100% isopropyl alcohol for 30 min. Subsequently, the embryos were stained with 0.5% Oil Red O (Sigma, USA) for 3 h at room temperature in dark. After staining, the embryos were washed again with PBS for 5 min.

### 2.7 Statistical analysis

All experiments were repeated at least three times. Significant differences between different types of embryos were calculated using a *t*-test. *, **, *** denote *p* < 0.05, <0.01, and <0.001, respectively.

## 3 Results

### 3.1 *Cobll1a* expression pattern during embryonic development in zebrafish

**FIGURE 1:**
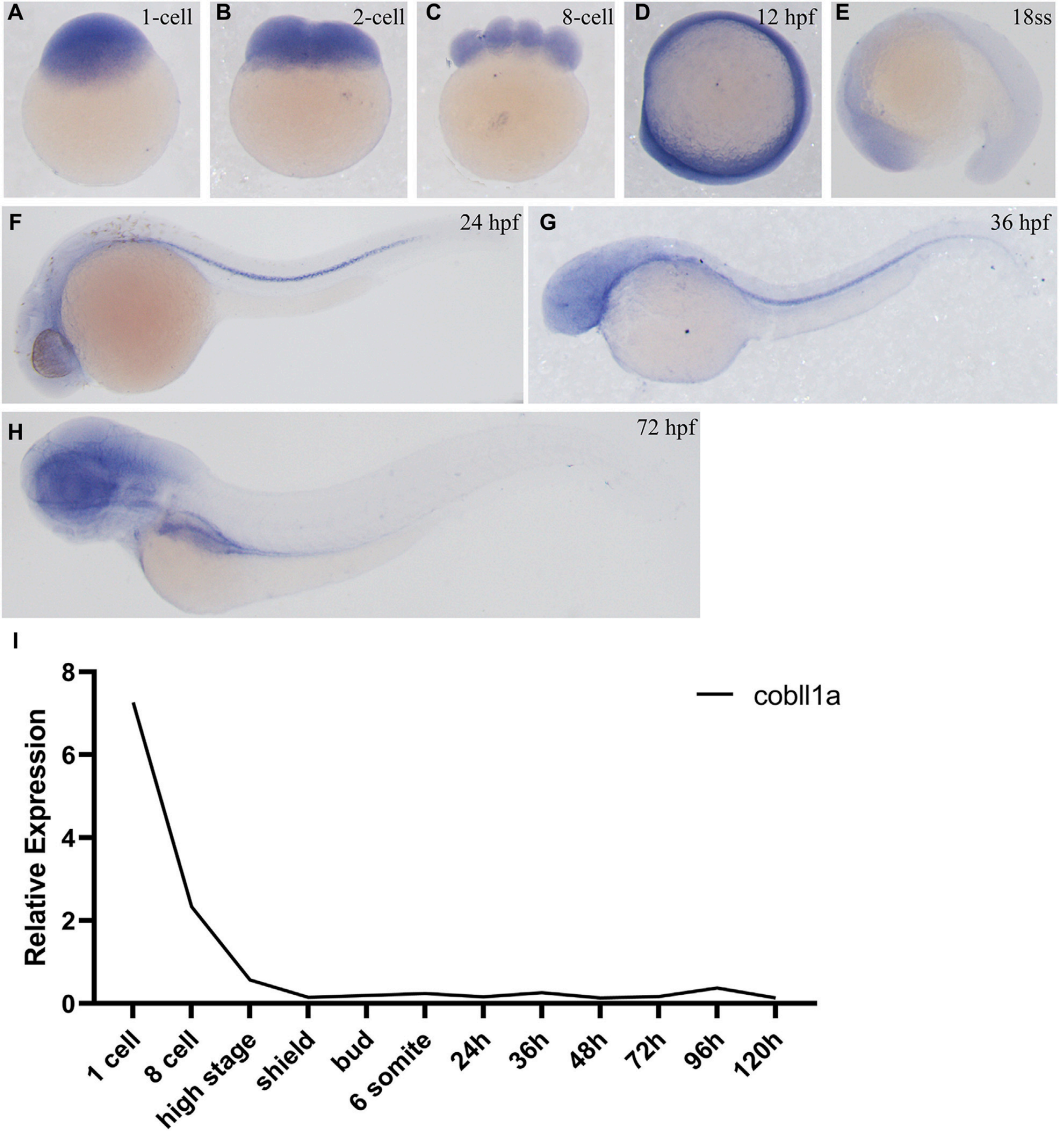
Expression pattern of *cobll1a*. **(A–H)** Spatio-temporal expression profile of the *cobll1a* gene detected by *in situ* hybridization in embryos. **(A–C)** *cobll1a* is expressed at the one to eight cell stages. **(D)** *cobll1a* is ubiquitously expressed at 12 hpf. **(E)** *cobll1a* is expressed in the head. **(F, G)** *cobll1a* was expressed in the notochord at 24 hpf and 36 hpf. **(H)** *cobll1a* is expressed in the eye, brain, liver and digestive system in 72 hpf embryos. **(I)** Using *18S* as the reference gene, RT-qPCR results demonstrated the relative expression of the *cobll1a* at different stages.

In order to investigate the role of *cobll1a* in early zebrafish development, we employed Whole-Mount *In Situ* Hybridization (WISH) to observe the spatial-temporal expression pattern of *cobll1a*. WISH results demonstrated that *cobll1a* was expressed in the one-cell stage and eight-cell stage embryos (Figures 1A–C), ubiquitously expressed at 12 hpf (Figure 1D) and localized in the head at 18 somite stage (ss) (Figure 1E). Expression of *cobll1a* was detected in the spinal cord at 24 hpf and 36 hpf (Figures 1F, G), and notably enriched in the eye, brain, liver, and digestive organs at 72 hpf (Figure 1H). Additionally, RT-qPCR was utilized to measure *cobll1a* mRNA expression level at different developmental stages (Figure 1I). The RT-qPCR data revealed that *cobll1a* mRNA was highly expressed at the one-cell stage and gradually decreased, which was consistent with the WISH results (Figures 1A–H). These findings suggest that *cobll1a* may be involved in early development in zebrafish.

### 3.2 Abnormal embryonic development due to *cobll1a* gene knockout

**FIGURE 2:**
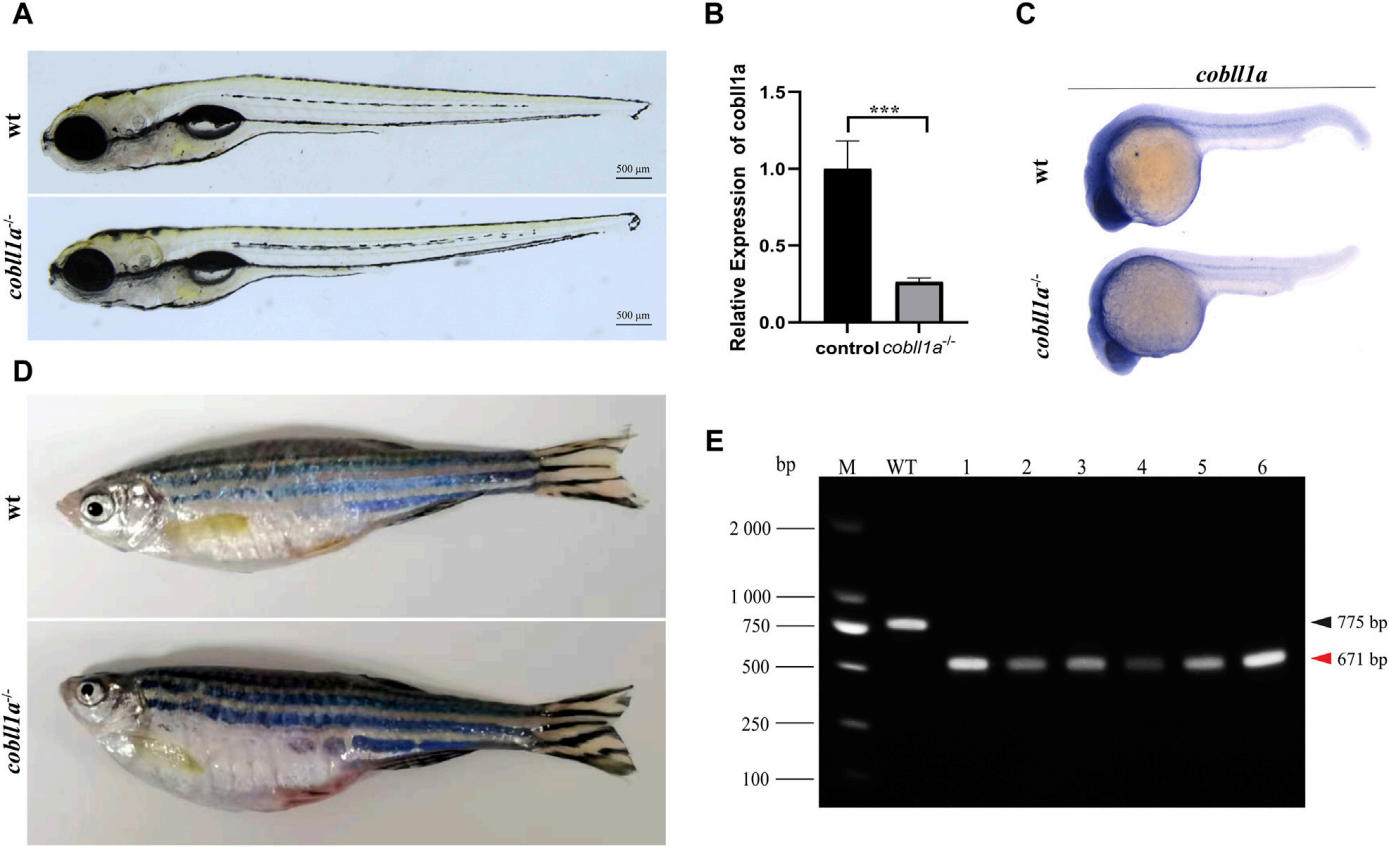
Loss of *cobll1a* results in a microcephalic phenotype. **(A)** *cobll1a* ^−/−^ larvae developed normally at 5 dpf. **(B)** Transcriptional expression level of *cobll1a* in *cobll1a* ^−/−^ embryos at 52 hpf were scrutinized by RT-qPCR, compared with the control. **(C)** An RNA probe of *cobll1a* was utilized *in situ* hybridization of WT and *cobll1a* ^−/−^ embryos at 24 hpf. **(D)** *cobll1a* ^−/−^ juveniles exhibited an enlarged abdomen and smaller eyes and head at 8 months. **(E)** Genotyping results of *cobll1a* ^−/−^ as shown in Figure 2A.

To elucidate the role of the *cobll1a* gene, CRISPR/Cas9 gene editing technology was employed to generate *cobll1a* mutant line. Initially, we conducted a bioinformatics analysis of the gene, selected a pair of knockout targets separated by 134 bp on exon4 (Supplementary Figure S2A), and established three independent mutant lines from different F0 mutants. *Cobll1a* line1 harbored a 2 bp insertion at target site 1 and a 77 bp deletion at target site 2 (Supplementary Figure S2B), *cobll1a* line2 had a a 4 bp deletion at target site 1 and a 100 bp deletion at target site 2 (Supplementary Figure S2C), while *cobll1a* line3 displayed a seven bp deletion at target site one and a 77 bp deletion at target site 2 (Supplementary Figure S2D). These alterations resulted in a frame shift in the open reading frames. In *cobll1a* line1, an early stop codon resulted in a truncated COBLL1A protein consisting of 104 amino acids (Supplementary Figure S2B). In *cobll1a* line2, the COBLL1A protein was altered at the 35th amino acid and translation was terminated after an additional 34 amino acids (Supplementary Figure S2C); whilst in *cobll1a* line3, the COBLL1A protein was altered at the 70th amino acid and ceased translation after an additional 31 amino acids (Supplementary Figure S2D). These nonsense mutations resulted in a loss of function of the *cobll1a* gene in all three mutant lines (Supplementary Figures S2B-S2D). Heterozygotes from these three lines exhibited no obvious phenotype and were able to mature into adult. The impact of the *cobll1a* gene’s impact on zebrafish development was studied by incrosing the *cobll1a* ^−/−^ and observed for 5 days. All the embryos of the three mutant lines developed normally, no significant difference was observed in the overall development of the 5 dpf *cobll1a* ^−/−^ homozygous mutant embryos compared to the control (Figure 2A). Given that each line resulted in a truncated *COBLL1A* protein, Line2 was chosen for subsequent experiments. To further confirm if *cobll1a* was knocked out successfully, the mRNA expression level of *cobll1a* was examined by RT-qPCR experiment. The results showed that a significant reduction in *cobll1a* gene expression was observed in *cobll1a* ^−/−^ embryos compared to the control group *p* < 0.001 (Figure 2B). Moreover, we used WISH to detect the expression level of *cobll1a* in WT and *cobll1a* ^−/−^ embryos, and the results showed that the expression of *cobll1a* in 24 hpf *cobll1a* ^−/−^ embryos decreased, compared to the control (Figure 2C). To validate the genotype, genomic DNA was extracted, followed by PCR and agarose gel electrophoresis. Results from the agarose gel electrophoresis showed that in lanes one to six, a band of 671 bp was observed, indicating that all of them are *cobll1a* ^−/−^ (Figure 2E). The 8-month-old *cobll1a* ^−/−^ adult fish exhibited an enlarged abdomen, more diminutive eyes, and smaller head phenotype, in contrast to the control (Figure 2D).

### 3.3 Loss of *cobll1a* impacts liver development

**FIGURE 3:**
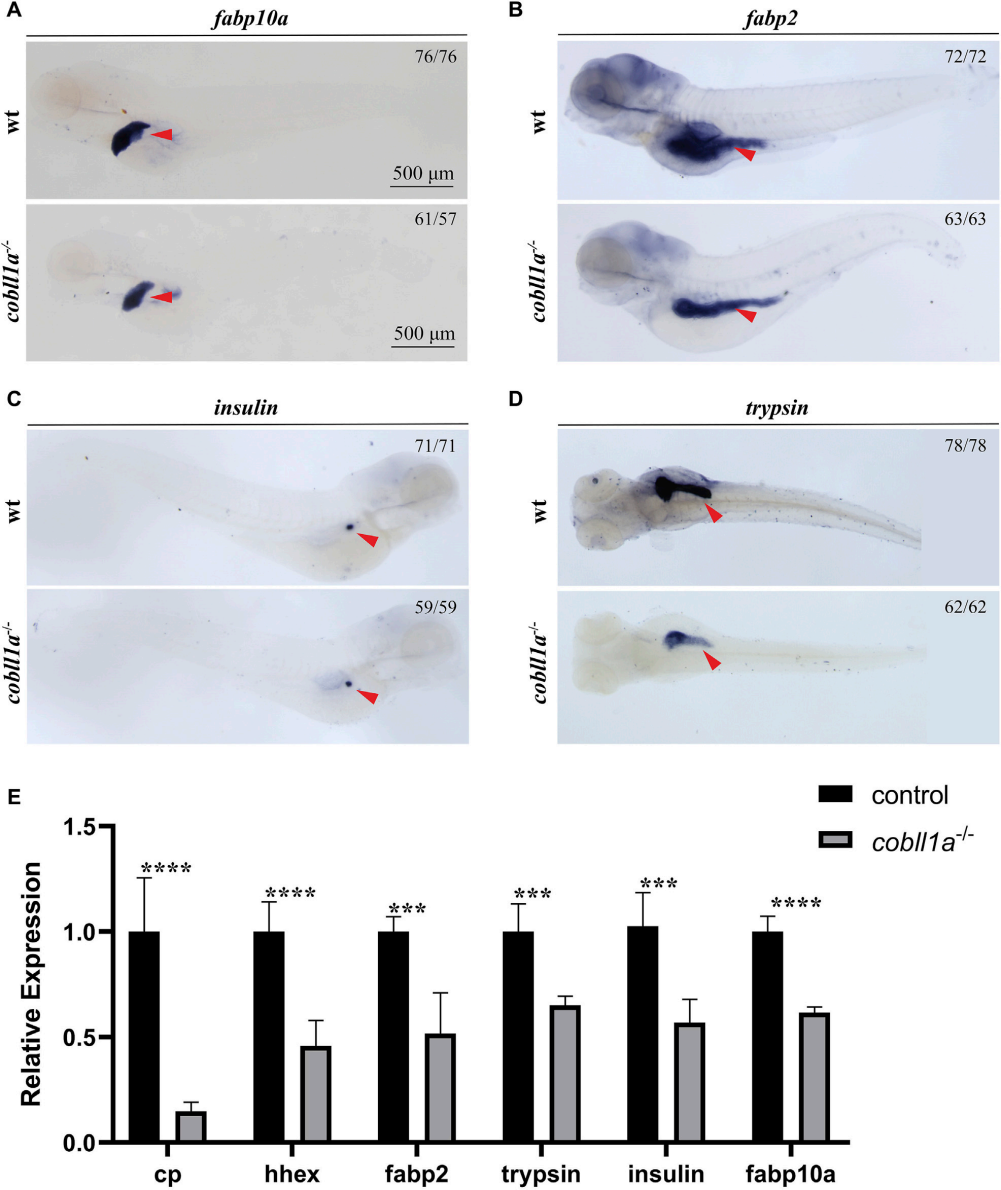
Depicts the abnormal liver development observed in the absence of *cobll1a*. **(A)** An RNA probe of *fabp10a* was utilized *in situ* hybridization of WT and *cobll1a* ^−/−^ embryos at 4 dpf. **(B–D)** WT and *cobll1a* ^−/−^ embryos at 4 dpf were detected with anti-sense RNA probes: intestinal marker *fabp2* **(B)**, endocrine pancreatic marker *insulin* **(C)**, and excretory pancreatic marker *trypsin* **(D)** through WISH. **(E)** The mRNA expression level of hepatopancreatic-intestinal development-related genes in WT and *cobll1a* ^−/−^ embryos were analyzed using RT-qPCR at the 52 hpf stage.

*In situ* hybridization results showed that *cobll1a* was expressed in the liver and other digestive organs at 72 hpf. Previous studies have shown that abnormalities in the human *cobll1* gene lead to defects in glucose and lipid metabolism (Desmarchelier et al., 2014). Since the liver plays a crucial role in metabolism, we proposed that the *cobll1a* mutation in zebrafish may influence liver development and metabolism. To test this hypothesis, RT-qPCR was performed to measure the expression levels of liver development-related genes in 52 hpf *cobll1a* ^−/−^ embryos. The results indicated a decreased expression of hepatic progenitor cell markers *hhex* and *cp* in *cobll1a* mutants, compared to the control (Figure 3E). This suggests that the loss of *cobll1a* may led to abnormal liver development in zebrafish. It is reported that zebrafish’s foregut endoderm cells develop into progenitor cells of the liver, pancreas, and intestine, forming organ buds at specific locations before maturing into organs (Field et al., 2003). Whether the *cobll1a* mutation also affects the development of other digestive organs remains unclear. The liver cell-specific expression gene *fabp10a*, the early intestinal development marker *fabp2*, the exocrine pancreatic development marker *trypsin*, and the endocrine pancreatic marker *insulin* were examined. RT-qPCR results showed that the expression level of marker genes in digestive organs was reduced compared to the control (Figure 3E). To further verify the results that *cobll1a* mutation led to abnormal hepato-pancreatic intestine development in zebrafish, we used antisense digoxin-labeled RNA probes for WISH. The WISH results showed that the expression of *fabp10a, fabp2*, *insulin*, and *trypsin* in *cobll1a* ^−/−^ embryos were decreased (Figures 3A–D), which is consistent with the RT-qPCR results.

### 3.4 The role of *cobll1a* in zebrafish earlydevelopment using a transcriptome profile

To uncover the molecular mechanism of *cobll1a* in liver development, three groups of 52 hpf wild type and *cobll1a* ^−/−^ zebrafish embryos were collected, with 50 embryos in each group. The specimens were sent to Shanghai Ouyi Biomedical Technology Co, Ltd. For high-throuphput transcriptome sequencing.

**FIGURE 4:**
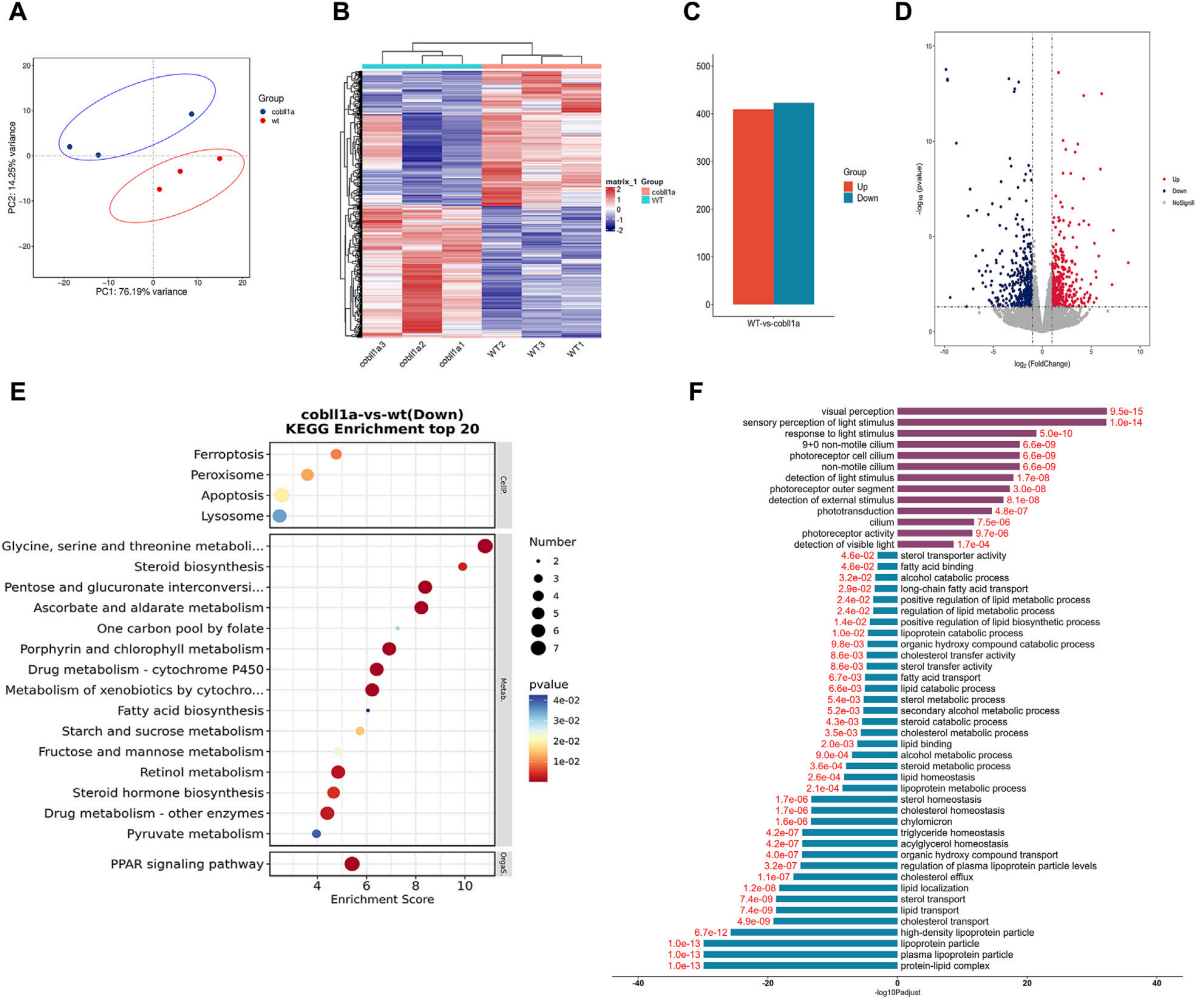
The DEGs analysis of WT and *cobll1a* ^−/−^ zebrafish embryo transcriptomes. **(A)** PCA cluster analysis of WT and *cobll1a* mutant zebrafish embryos demonstrated a significant correlation among three biological replicates in both the WT and *cobll1a* mutant groups. **(B)** Clustering heat maps of WT and *cobll1a* ^−/−^ were divided into two distinct groups. **(C)** The total number of differential genes in WT and *cobll1a* mutant zebrafish embryo samples was represented, with red denoting 409 up-regulated genes and blue indicating 423 down-regulated genes. **(D)** The volcano map displays DEGs between the two groups, with red representing up-regulated genes, blue signifying down-regulated genes, and gray denoting non-DEGs. Each dot represents a gene. **(E)** KEGG analysis of down-regulated DEGs revealed a significantly enriched pathway. **(F)** GO enrichment analysis of DEGs provides a description of their functions. Purple indicates up-regulated genes, blue represents down-regulated genes, and red fonts depict corrected *p*-values. Black font is used for the GO feature comment.

Transcriptome sequencing and subsequent bioinformatics analysis yielded an average of approximately 500,000 reads per sample, all mapped to the reference genome. Principal Component Analysis (PCA) results visualized the relationship between experimental and control samples, depicting a pronounced separation between the two groups (Figure 4A). Heat maps of DEGs (Differentially Expressed Genes) were established using gplot analysis, distinctly segregating experimental and control samples into two separate groups (Figure 4B). The screening threshold was set as |log2Foldchange| ≥ 1 and *p*-value≤0.05. A total of 832 genes were identified, of which 423 were down-regulated and 409 were up-regulated (Figure 4C). Volcano maps were used to illustrate the distribution of up-regulated and down-regulated genes (Figure 4D). Functional enrichment analysis of DEGs revealed significant enrichment in KEGG (Kyoto Encyclopedia of Genes and Genomes) pathways such as “retinol metabolism”, “steroid hormone biosynthesis”, “drug metabolism-cytochrome P450”, and “PPAR signaling pathway” (Figure 4E). This suggests that the *cobll1a* knockout results in abnormal retinol and lipid metabolism. Additionally, 235 significantly enriched GO terms were identified, notably associated with the liver: “Lipoprotein complex”, “lipid metabolic process”, “cholesterol transport”, “lipid transport”, “triglyceride homeostasis”, “cholesterol effluence”, “cholesterol homeostasis”, “lipoprotein metabolic process”, “acylglycerol homeostasis”, “sterol transport”, “fatty acid binding” and other biological processes (Figure 4F). These results suggest that the deletion of the *cobll1a* gene disrupts lipid metabolism, lipid homeostasis, and lipid transport function, primarily affecting down-regulated genes enrichment. To further verify liver-related genes, DEGs such as *apoa1a*, *apoa1b*, *apoa4a*, and *apoa4b.1* were selected from GO and KEGG for validation.

### 3.5 *Cobll1a* absence triggers disturbances in retinoic acid metabolism

**FIGURE 5:**
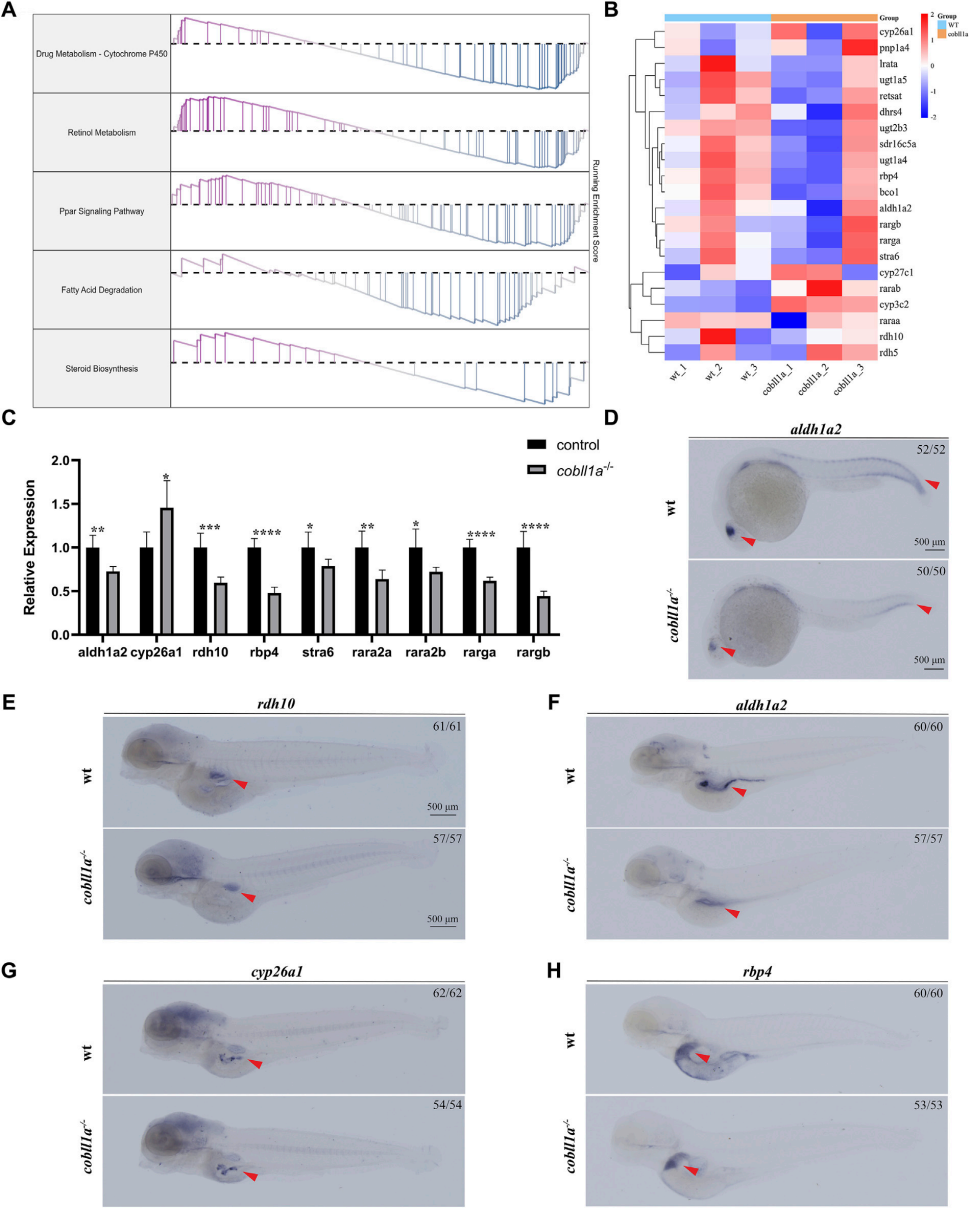
Loss of *cobll1a* leads to abnormal RA metabolism in zebrafish. **(A)** Line chart of GSEA results with the right ordinate representing the enrichment score. **(B)** Cluster heat maps of RA metabolism-related genes in DEGs, displaying significant differences between WT and *cobll1a* mutant. **(C)** RT-qPCR analysis of the transcriptional expression level of RA metabolism-related genes at 52 hpf *cobll1a* ^−/−^ compared with WT. **(D, F)** The expression of *aldh1a2* was decreased in *cobll1a* ^−/−^embryos, observed in eye and somatic cells at 24 hpf, in liver and intestinal tissues at four dpf. **(E)** At 4 dpf, the expression of *rdh10* was decreased in *cobll1a* ^−/−^ embryos. **(G)** At 4 dpf, the expression of *cyp26a1* was increased in *cobll1a* ^−/−^ embryos. **(H)** At 4dpf, the expression of *rbp4* was decreased in *cobll1a* ^−/−^ embryos.

Analysis of RNA-seq results revealed an abnormality in the RA metabolism following *cobll1a* mutation, including the PPAR signaling pathway and cytochrome p450 signaling pathway (Figure 5A). Consequently, focus shifted to the RA metabolic pathway. Retinol, oxidized to retinal by retinol dehydrogenase 10 (*rdh10*) (Metzler and Sandell, 2016), undergoes further dehydrogenation by retinal dehydrogenase to yield RA (Kedishvili, 2016). Humans and mice possess three variants of retinal dehydrogenases: *aldh1a1*, *aldh1a2*, and *aldh1a3*(Cunningham and Duester, 2015). However, zebrafish lack the *aldh1a1* gene, and RA is primarily synthesized by *aldh1a2*(Grandel et al., 2002). Humans and mice encompass three RA receptors (RARα, β, γ), while zebrafish only have two homologues of RARα (*raraa* and *rarab*), two of RARγ (*rarga* and *rargb*), lacking the RARβ gene (Kastner et al., 1995). Genes associated with RA metabolism, such as *aldh1a2* and *rdh10*, were detected among the DEGs. Consequently, we selected significantly altered RA metabolism-related genes for cluster analysis (Figure 5B) and RT-qPCR.Figure 5C illustrates significant changes in the expression of RA metabolism-related genes compared to the control. RT-qPCR results revealed a down-regulation in the expressions level of RA anabolic genes *rdh10* and *aldh1a2*, and an up-regulation in the expression level of the RA catabolism gene *cyp26a1*. Additionally, the expression level of RARs genes *raraa*, *rarab*, *rarga*, and *rargb* were markedly down-regulated (*p* < 0.05) (Figure 5C). The expression level of retinol-binding protein *rbp4* and retinoid-inducing protein *stra6*, responsible for retinol circulation, were significantly down-regulated (Figure 5C).

The expression pattern of *rdh10*, *aldh1a2*, *cyp26a1*, and *rbp4* were detected using WISH, the results showed that they were expressed in intestine or liver (Figures 5E–H). Furthermore, the expression of *aldh1a2* was reduced in 24 hpf *cobll1a* ^−/−^ embryos compared to control (Figure 5D). The expressions of *rdh10*, *aldh1a2*, and *rbp4* in 4 dpf *cobll1a* ^−/−^ embryos were down-regulated (Figures 5E, F, H), and *cyp26a1* was up-regulated (Figure 5G), corroborating the RT-qPCR results.

### 3.6 *Cobll1a* deficiency leads to hepatic lipid accumulation

**FIGURE 6:**
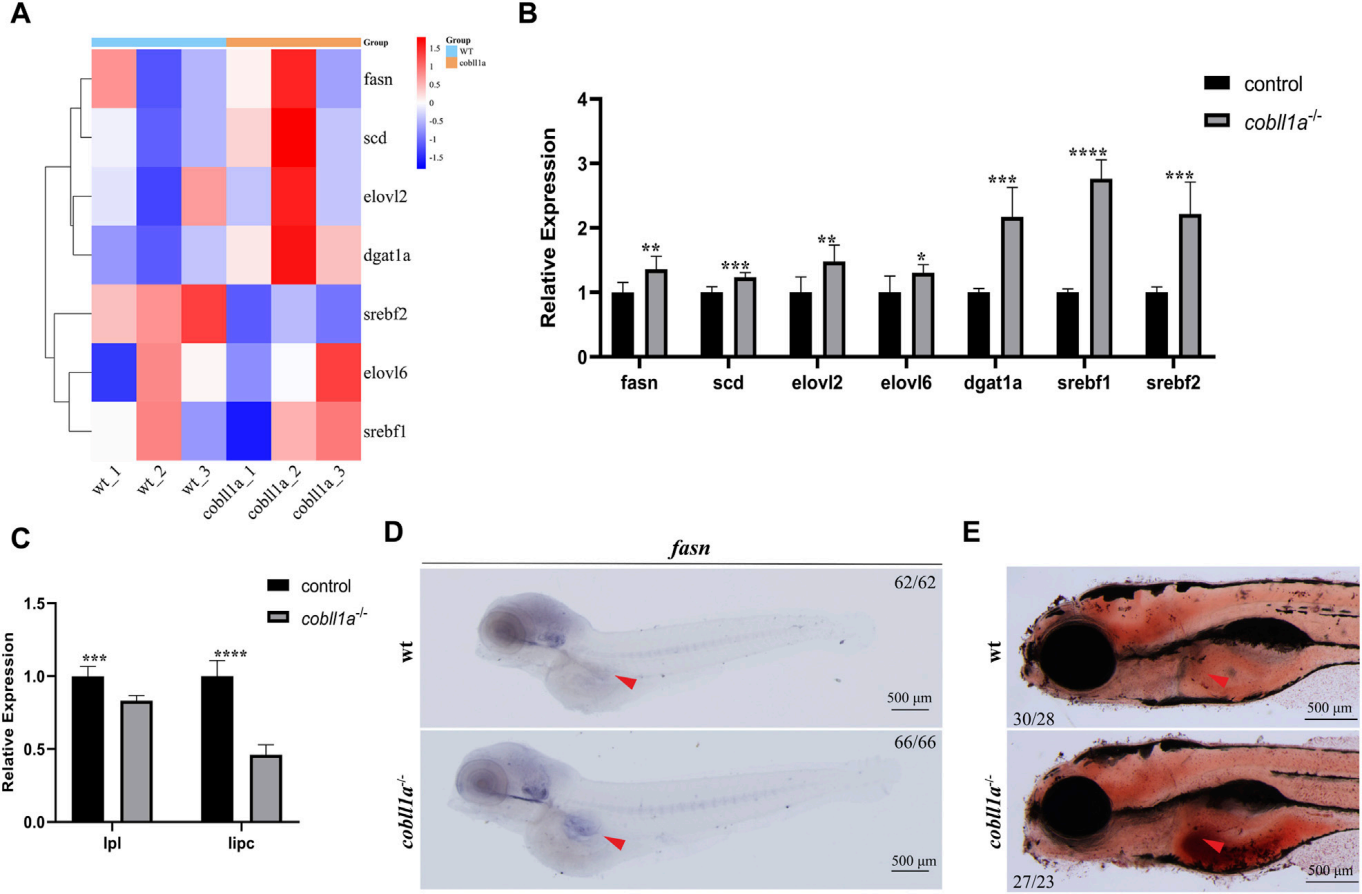
Loss of *Cobll1a* resulted in Abnormal Lipid Metabolism in Zebrafish. **(A)** Clustering heat maps of lipid anabolism-related genes in DEGs. WT and *cobll1a* mutated zebrafish embryo samples form two distinct groups. **(B)** RT-qPCR used to assess transcriptional expression level of lipid anabolism-related genes in *cobll1a* ^−/−^ embryos at 52 hpf, compared with WT. **(C)** RT-qPCR analysis of transcriptional expression level of lipid catabolism-related genes in *cobll1a* ^−/−^ embryos at 52 hpf, compared to the control. **(D)** At 4 dpf, *fasn* is expressed in adipocytes and the expression of *fasn* is up-regulated in *cobll1a* ^−/−^ embryos. **(E)** At 5 dpf, Oil Red O staining was used to assess the lipid distribution and content of WT and *cobll1a* ^−/−^ embryos.

Steatosis, a defining feature of NAFLD, progressively evolves into steatohepatitis and fibrosis (Angulo, 2002). It is chiefly characterized by lipid accumulation, including non-esterified fatty acids (NEFA), triglycerides, and non-esterified cholesterol. Studies suggest that exogenous RA treatment in wild-type mice results in decreased lipogenesis and enhanced catabolism (Amengual et al., 2010). Some researchers posit a potential correlation between liver triglyceride levels and reduced liver retinol in NAFLD (Chaves et al., 2014). Prior studies reveal that *cobll1a* mutation impacts RA metabolism and inhibits the RA signaling pathway. So we hypothesized that *cobll1a* mutation may result in an increase in hepatic fat anabolism, leading to steatosis and subsequent NAFLD progression. To verify whether *cobll1a* mutations contribute to enhanced hepatic fat anabolic metabolism, we analyzed fatty acid synthase (*fasn*), stearoyl-coA desaturase (*scd*), and elongation of very long-chain fatty acids protein two and six (*elovl2*, *elovl6*) using RT-qPCR. We also assessed the expression level of diacylglycerol oacyltransferase 1a (*dgat1a*) and sterol regulatory elements-binding transcription factor 1 and 2 (*srebf1*, *srebf2*) genes, crucial nuclear transcription factors regulating liver lipid metabolism. The cluster heat map and RT-qPCR results revealed significant up-regulation of *fasn*, *scd*, *elovl2*, *elovl6*, *dgat1a*, *srebf1*, and *srebf2* genes in *cobll1a* ^−/−^ embryos compared to the control group, suggesting increased fat anabolism in *cobll1a* ^−/−^ embryos (Figures 6A, B). Moreover, the expression level of lipoprotein lipase (*lpl*) and liver lipase (*lipc*) was significantly down-regulated, indicating reduced lipid catabolism (Figure 6C). Subsequently, we utilized WISH to detect *fasn* expression. WISH results displayed upregulation of *fasn* expression in *cobll1a* ^−/−^ embryos (Figure 6D). Next, we used oil red O staining to detect the distribution and content of fat in *cobll1a* mutant and the control embryos, and the results showed that the oil red O positive region in *cobll1a* mutant was enlarged and there was obvious lipid accumulation compared with the control (Figure 6E).

### 3.7 *Cobll1a* deletion impairs lipid transport

The transport of lipids such as cholesterol, cholesterol esters, and triglycerides, which are fundamentally insoluble in aqueous environments, is dependent on their association with the lipoprotein complex (Illingworth, 1993). Plasma lipoprotein, a composite of proteins and lipids, is responsible for transporting dietary lipids from the small intestine to the liver, muscle, and adipose tissue. It also plays an instrumental role in lipid transportation from the liver to other tissues and in cholesterol reverse transport from peripheral tissues to the liver and intestine (Feingold, 2022). There are four primary types of plasma lipoproteins, namely, chylomicrons (CMs), very low-density lipoproteins (VLDL), low-density lipoproteins (LDL), and high-density lipoproteins (HDL). Apolipoproteins, the protein constituents of lipoprotein, include apoE, apoB, apoA-I, apoA-II, apoA-IV, apoC-I, etc. They contribute to the transport and redistribution of lipids between various types of cells and tissues, either as co-factors of lipid metabolic enzymes or by maintaining the structural stability of lipoprotein particles (Otis et al., 2015).

**FIGURE 7:**
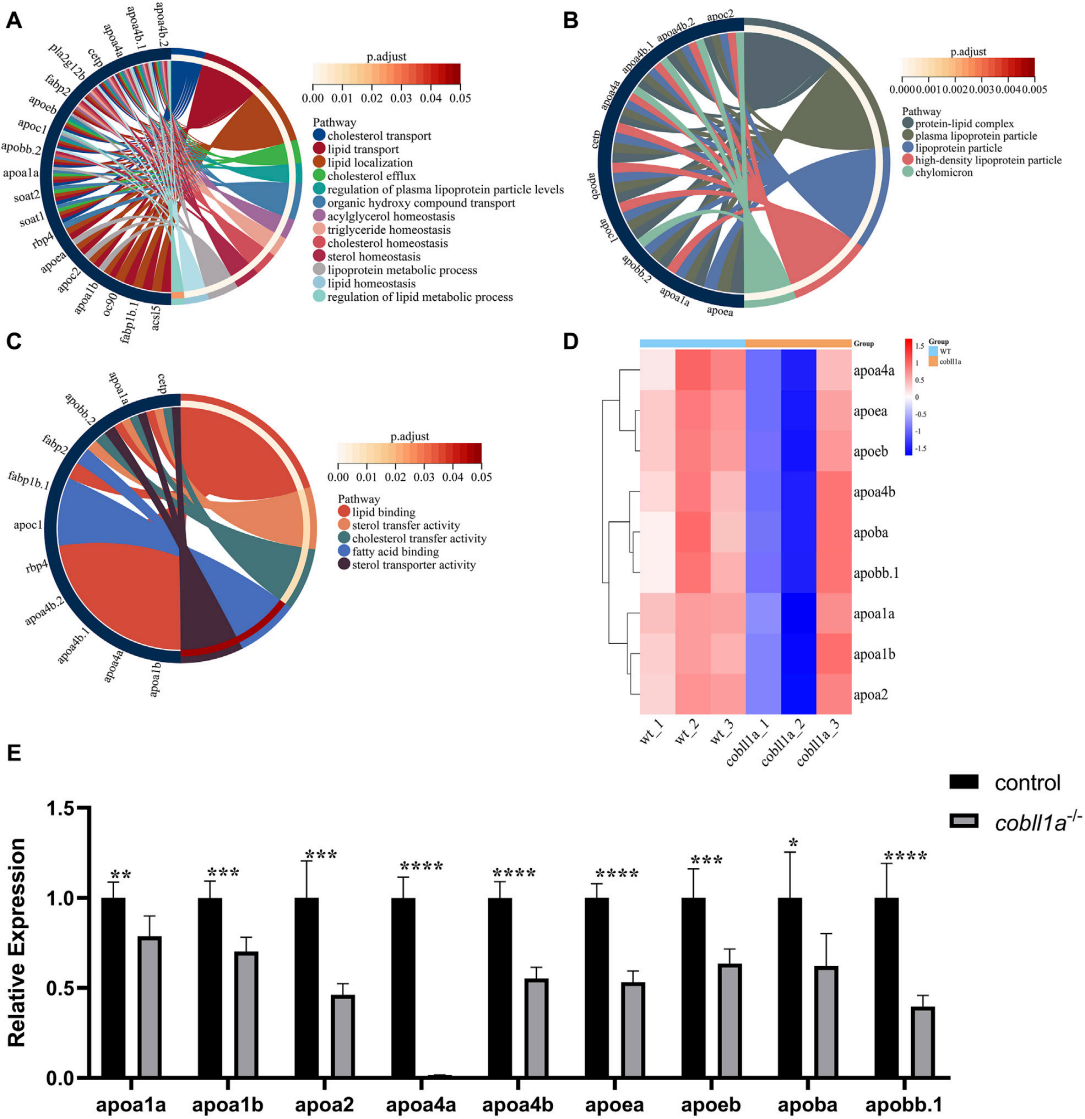
Zebrafish embryos exhibit abnormal lipoprotein metabolism in *cobll1a* mutant. **(A–C)** Enrichment analysis of GO molecular function (MF), biological process (BP), and cell component (CC) of biological processes in *cobll1a* ^−/−^ embryos. **(D)** Cluster heat maps of genes associated with lipoprotein metabolism, where samples from WT and *cobll1a* mutant zebrafish embryos are segregated into two distinct groups. **(E)** Transcriptional expression level of apolipoprotein metabolism-related genes in 52 hpf *cobll1a* ^−/−^ embryos were analyzed by RT-qPCR compared with WT.

Our RNA-seq data identified a number of apolipoprotein-related genes in DEGs, with apolipoprotein expression in *cobll1a* ^−/−^ embryos significantly down-regulated compared to the control. GO gene enrichment analysis related to lipoproteins revealed that the primary biological processes affected were cholesterol transport, lipid transport, lipid localization, and triglyceride homeostasis, among others (Figure 7A). Abnormalities were observed in cellular components such as the lipoprotein complex, plasma lipoprotein particles, and lipoproteins (Figure 7B). Molecular functions like lipid binding, sterol transport activity, cholesterol transport activity, and fatty acid binding were also affected (Figure 7C). These GO analysis results indicated disturbances in lipoprotein metabolism and abnormalities in lipid transport, cholesterol efflux, and reverse transport upon *cobll1a* deletion. Cluster heat map analysis of apolipoproteins in DEGs showed significant differences in the expression of apolipoproteins between WT and *cobll1a* ^−/−^ embryos (Figure 7D). RT-qPCR was subsequently utilized to validate the expression level of *apoa1a*, *apoa1b*, *apoa2*, *apoa4a*, *apoa4b*, *apoea*, *apoeb*, *apoba*, *apob.1*, as depicted in Figure 7E, demonstrating a significant reduction in the expression of the related apolipoprotein genes.

## 4 Discussion

In human, *COBLL1* is predominantly expressed in several organs including islets, kidneys, skeletal muscle, liver, mast cells, adipocytes, placenta, and esophagus (Plešingerová et al., 2018). The polymorphic sites of *COBLL1*, rs10195252 and rs3923113, are implicated in the regulation of fasting circulating levels of triglycerides and high-density lipoprotein cholesterol (Teslovich et al., 2010). This study elucidates the critical role of *cobll1a* in liver and RA metabolism in zebrafish. Zebrafish *hhex* is expressed in liver buds between 22 and 50 hpf, and is associated with a smaller liver in mutants (Wallace et al., 2001). The gene *cp*, expressed in hepatocytes at 32 hpf, serves as a specific molecular marker for early hepatocytes and the liver in zebrafish (Korzh et al., 2001). In *cobll1a* ^−/−^ mutant embryos, the expression of *hhex* and *cp* was significantly down-regulated compared to the control, indicating a reduction in liver progenitor cells, resulting in a small liver. In fact, the expression of the hepatocyte-specific gene *fabp10a* was significantly down-regulated at 72hpf, and there was a decrease in the size of both the intestine and pancreas. RNA-seq data revealed abnormal RA metabolism in *cobll1a* ^−/−^ mutant embryos, characterized by reduced RA synthesis. Prior studies have affirmed that RA is crucial for pancreatic and liver development in zebrafish, and its deficiency results in abnormal pancreas and liver development (Stafford and Prince, 2002). A deficiency in RA also induces microphthalmia and forebrain reduction in zebrafish embryos (Le et al., 2012), which is also observed in *cobll1a* ^−/−^ embryos.

To elucidate the molecular mechanism underpinning the abnormal liver development in *cobll1a* ^−/−^ mutants, RNA-seq was conducted. Results showed that down-regulated DEGs were predominantly concentrated in PPAR signaling pathway, cytochrome P450 signaling pathway, and other pathways involved in RA metabolism, cholesterol metabolism, and lipid metabolism. Peroxisome proliferator-activated receptors (PPARs) signaling pathway, a class of nuclear receptors, plays a crucial role in regulating lipid homeostasis during both development and adulthood, and disruption of PPAR signaling has been associated with diseases such as obesity and glucose intolerance (Rees et al., 2008). PPARs heterodimerize with the retinoid X receptor (RXR) to bind to the peroxisome proliferator response element (PPRE) promoter sequence, enabling the transcription of genes associated with fatty acid metabolism (Monsalve et al., 2013). RXR, a potent drug target for the treatment of metabolic syndrome and cancer, is a key member of nuclear receptors (Zhang et al., 2011). RXR forms homo-dimers and hetero-dimers with a range of other nuclear receptors, including fatty acid receptors (PPARs), bile acid receptors (farnesoid x receptors, FXR, pregnane x receptor (PXR), constitutive androstane receptor (CAR), vitamin D receptor (VDR), and RA receptor (RAR), liver x receptor (LXR), allowing these dimers to regulate the transcription of target genes by binding to their promoter regions (Dawson and Xia, 2012). Most RXR partners have roles in regulating lipid homeostasis (He et al., 2013). As most of these receptors are abundantly expressed in the liver, endogenous RA may regulate many hepatic nuclear receptor-mediated pathways. Cytochrome P450 (CYPs), important heme-containing proteins, plays key roles in the metabolism of exogenous substances and endogenous compounds (Tompkins and Wallace, 2007).

The regulation of retinol homeostasis is facilitated by a complex network of enzymes and proteins involved in retinol transportation, synthesis, and catabolism (Moise et al., 2007). Its physiological function is primarily mediated by its metabolites, retinal and RA (Napoli, 2012). The balance of RA is managed by enzyme expression responsible for its synthesis and oxidative degradation (Ross, 2003). Notably, the liver RA level in *rdh10* ^+/−^ mice was significantly reduced, with increased liver triglycerides and decreased gene expression involved in fatty acid β-oxidation (Yang et al., 2018). Mice expressing liver-specific RARα dominant negative protein exhibited microalveolar steatosis at 4 months, decreased mitochondrial β-oxidized fatty acids, and developed hepatocellular carcinoma and hepatic adenoma at 1 month (Yanagitani et al., 2004). Clinically, retinol analogs have been found to inhibit the occurrence of secondary primary tumors in patients diagnosed with hepatocellular carcinoma (HCC) (Muto et al., 1996). Previous studies have revealed a significant loss of liver retinol levels in NAFLD (Zhong et al., 2019), with the liver retinol level inversely correlated with the severity of liver damage and liver fat (Chaves et al., 2014; Trasino et al., 2015). In both mice and human NAFLD, liver retinol levels are inversely associated with liver steatosis (Friedman et al., 2018).

*Fasn* plays a pivotal role in lipid synthesis, responsible for combining palmitic acid from acetyl-CoA and malonyl-CoA into long-chain saturated fatty acids (Angeles and Hudkins, 2016). The elovls family of ultralong chain fatty acid elongase are crucial enzyme that manage fatty acid metabolism in animals (Morcillo et al., 2011). *Elovl2* has been implicated in the biosynthesis of long-chain polyunsaturated fatty acids in mammals (Leonard et al., 2002), while *elovl6* primarily catalyzes palmitate and palmitoleate into stearate and oleate (Moon et al., 2014). *Dgat1a* facilitates the conversion of diacylglycerol and fatty acyl-CoA into triacylglycerol (Barbosa et al., 2019). *Srebf1* and *srebf2* are significant nuclear transcription factors that regulate liver lipid metabolism and are crucial regulatory factors of fatty acid metabolism (Rong et al., 2017). Overexpression of *srebf1* activates the synthesis of fatty acids and triglycerides (Horton et al., 2002), while overexpression of *srebf2* activates cholesterol biosynthesis and uptake, and slightly triggers fatty acid biosynthesis (Rong et al., 2017). After the *cobll1a* mutation in this study, the expression of *fasn*, *elovl2*, *elovl6*, *dgat1a*, *srebf1*, and *srebf2* genes was upregulated, thus promoting the production of new fat and eventually inducing steatosis (Anderson and Borlak, 2008).

Following absorption by intestinal cells, REs are carried and transported into circulation by CMs, HDL, LDL, and VLDL (Levi et al., 2012). Impairment in VLDL synthesis can result in the accumulation of triglycerides in the liver (Tanoli et al., 2004). Apolipoprotein B (ApoB) is a major component of CMs particles, VLDL, and LDL (Olofsson and Borèn, 2005), and defects in ApoB synthesis or secretion can induce high adipose degeneration in the liver (Di Filippo et al., 2014). Zebrafish embryonic hepatocytes exhibited lipid accumulation after the double mutation of apoBa and apoBb.1 of apoB genes (Templehof et al., 2021). Apolipoprotein A4 (ApoA4) is primarily expressed in the intestine and liver, reacts to fat absorption, and plays a role in regulating glucose homeostasis and lipid metabolism, thus reducing susceptibility to atherosclerosis (Li et al., 2021). ApoA4 knockout rats and mice demonstrated significant hepatic steatosis (Wang et al., 2019). Apolipoprotein AI (ApoAI), the main component of HDL, plays a crucial role in lipid transport and metabolism (Chistiakov et al., 2016). APOA1 promotes cholesterol efflux by interacting with the ATP-binding box (ABC) transporter (Navab et al., 2011). APOE, an exchangeable amphipathic apolipoprotein, binds to LDL receptors, thereby regulating lipid uptake (Mahley, 1988). Apoe-deficient mice developed severe atherosclerosis due to an increase in circulating LDL cholesterol (Nakashima et al., 1994). In this study, the expression of *apoa1a*, *apoa1b*, *apoa2*, *apoa4a*, *apoa4b*, *apoea*, *apoeb*, *apoba*, and *apobb.1* genes was significantly down-regulated after *cobll1a* mutation, indicating that the loss of *cobll1a* resulted in lipid transport disorders in the liver of juvenile zebrafish (Xu et al., 2021).

In summary, *cobll1a* plays a crucial role in the liver lipid metabolism of zebrafish, and its mutation severely impairs RA metabolism and normal lipid homeostasis.

## Data Availability

The data presented in the study are deposited in the NCBI Sequence Read Archive under accession SRA: https://dataview.ncbi.nlm.nih.gov/object/PRJNA1069604?reviewer=8sirsbvs21hj0a2cpo3fu6ea6j, accession number PRJNA1069604.
