# Supplementary figures and images for: Zebrafish cobll1a regulates lipid homeostasis via the RA signaling pathway
Source: Front Cell Dev Biol. 2024 Apr 18;12:1381362. doi: 10.3389/fcell.2024.1381362 (PMC11063382; doi:10.3389/fcell.2024.1381362)

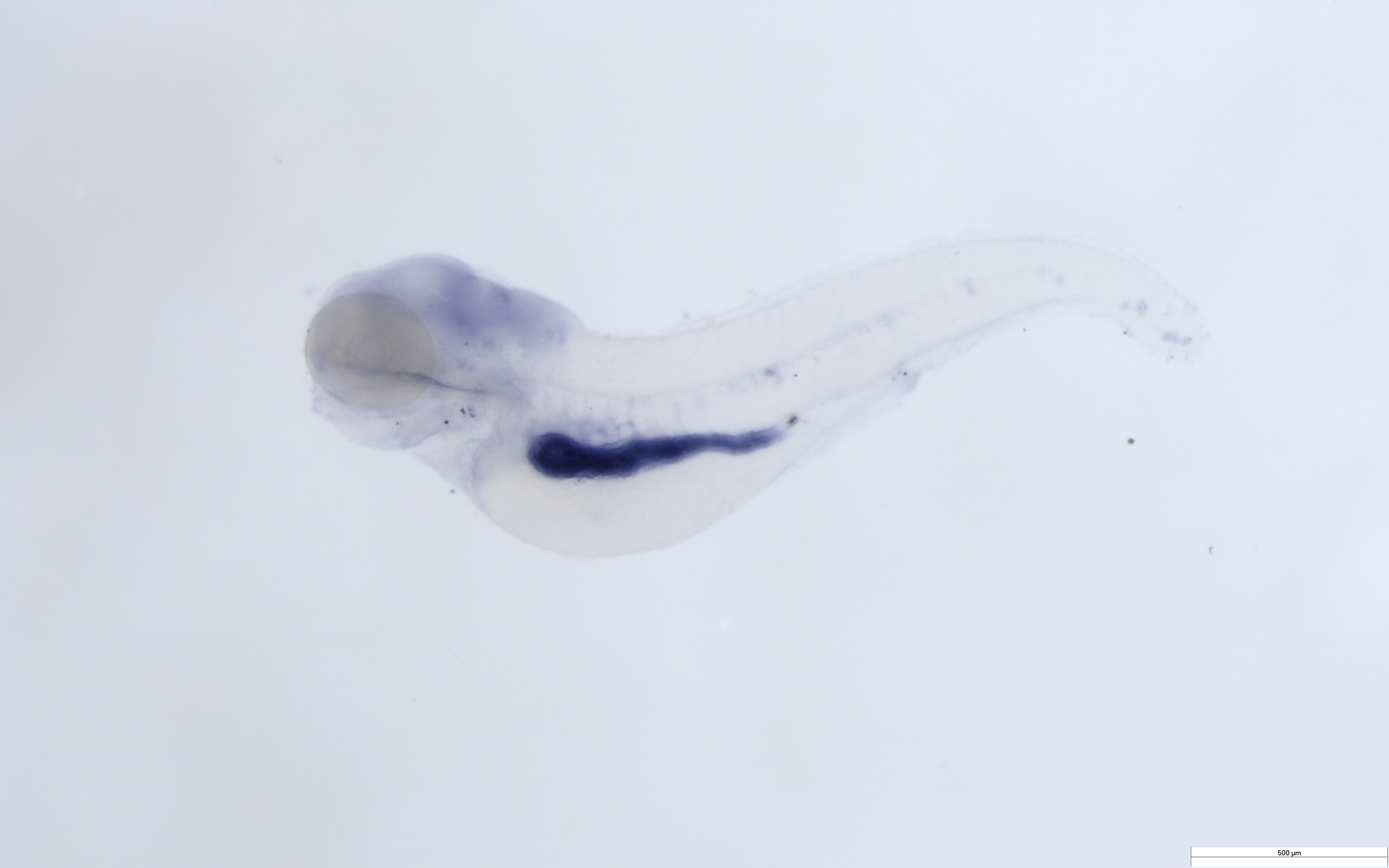

Supplement: Supplementary file 1 [file DataSheet3.ZIP › fig3/cobll1a mutant ifabp 4d 3.jpg]

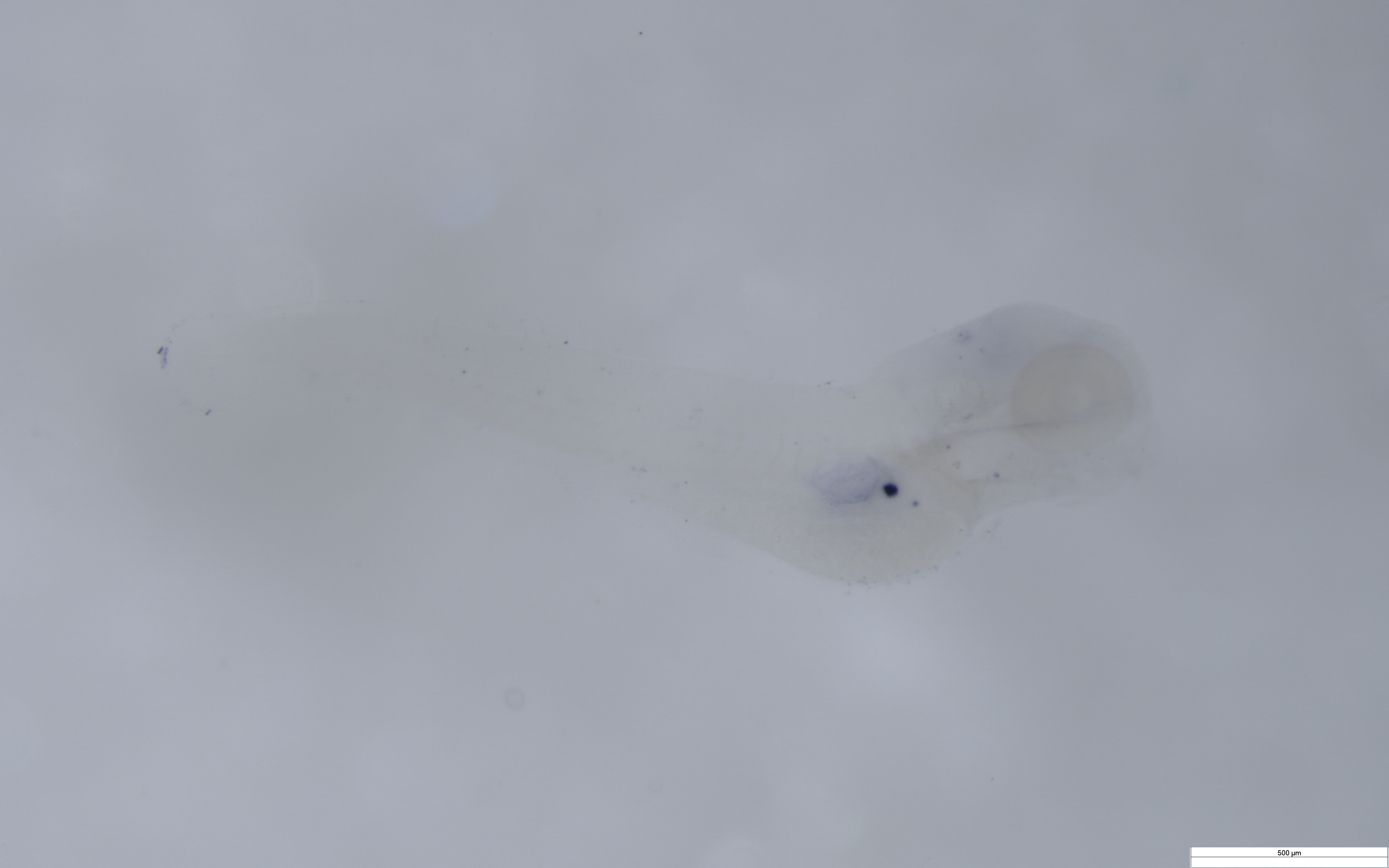

Supplement: Supplementary file 1 [file DataSheet3.ZIP › fig3/cobll1a mutant ins 4d 3.jpg]

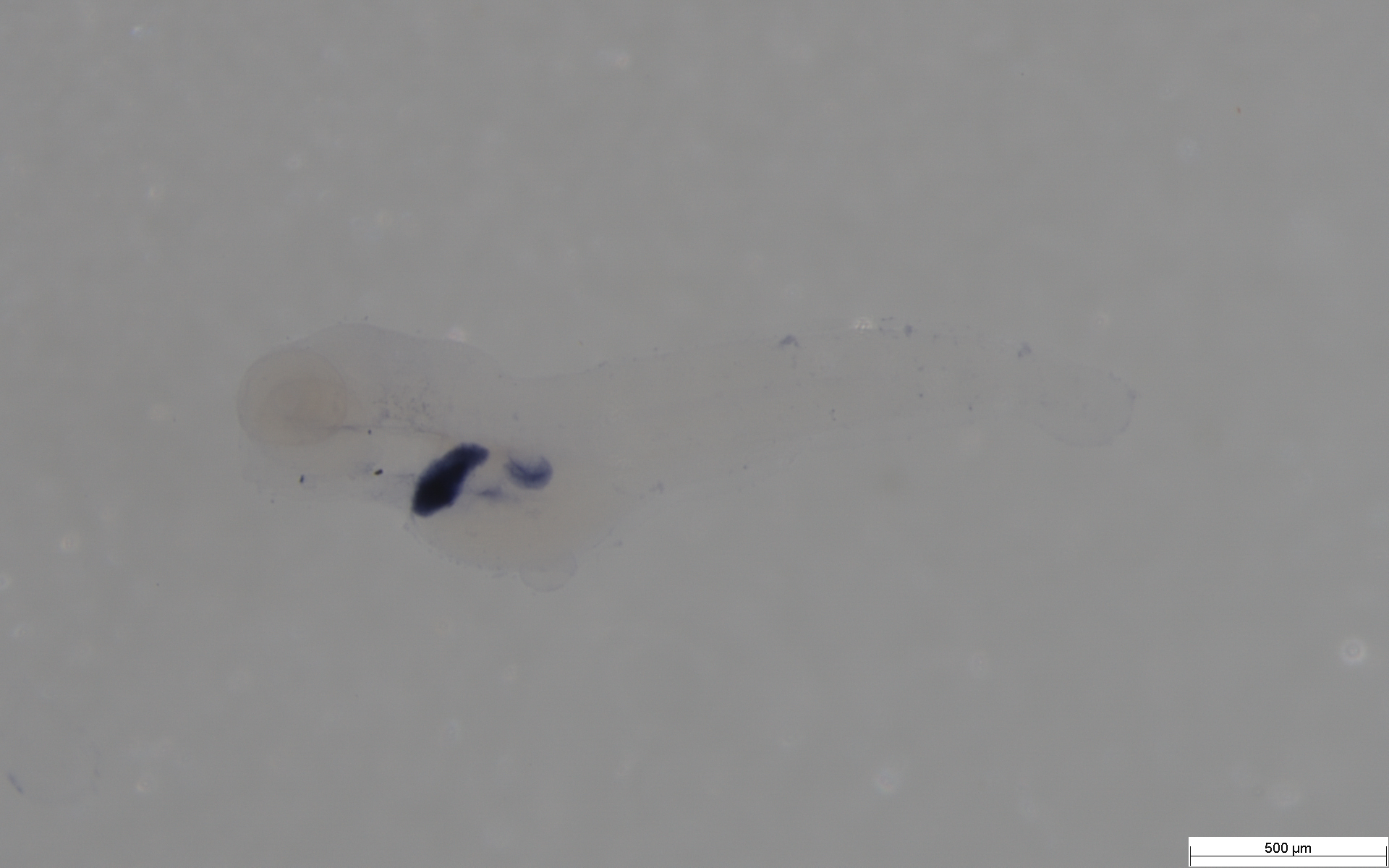

Supplement: Supplementary file 1 [file DataSheet3.ZIP › fig3/cobll1a mutant lfabp 4d e3_ch00.jpg]

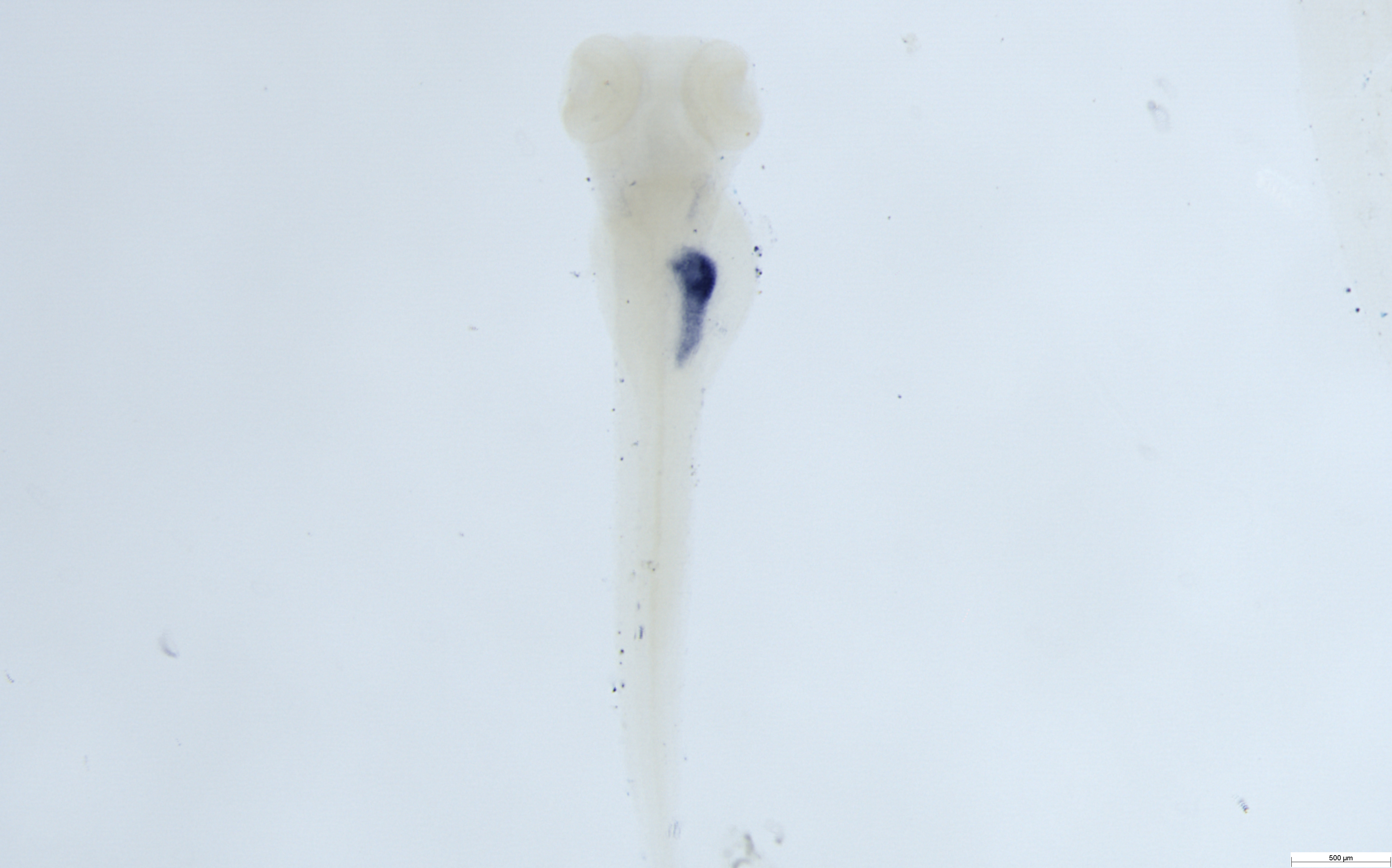

Supplement: Supplementary file 1 [file DataSheet3.ZIP › fig3/prss1-cobll1a mut 4d 3.2x e1-1 2023.10.jpg]

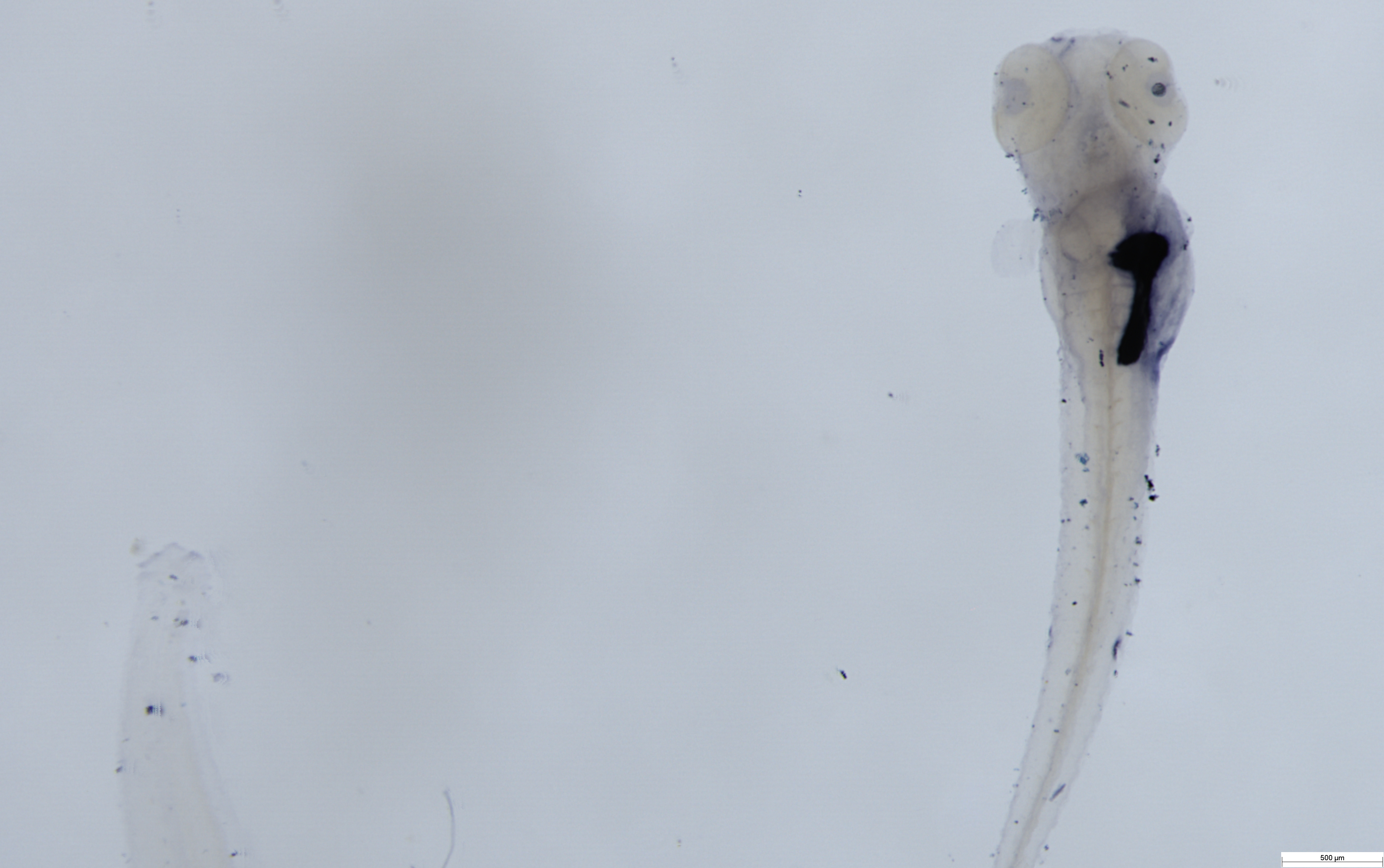

Supplement: Supplementary file 1 [file DataSheet3.ZIP › fig3/prss1-TU con 4d 3.2x e3-2 2023.10.jpg]

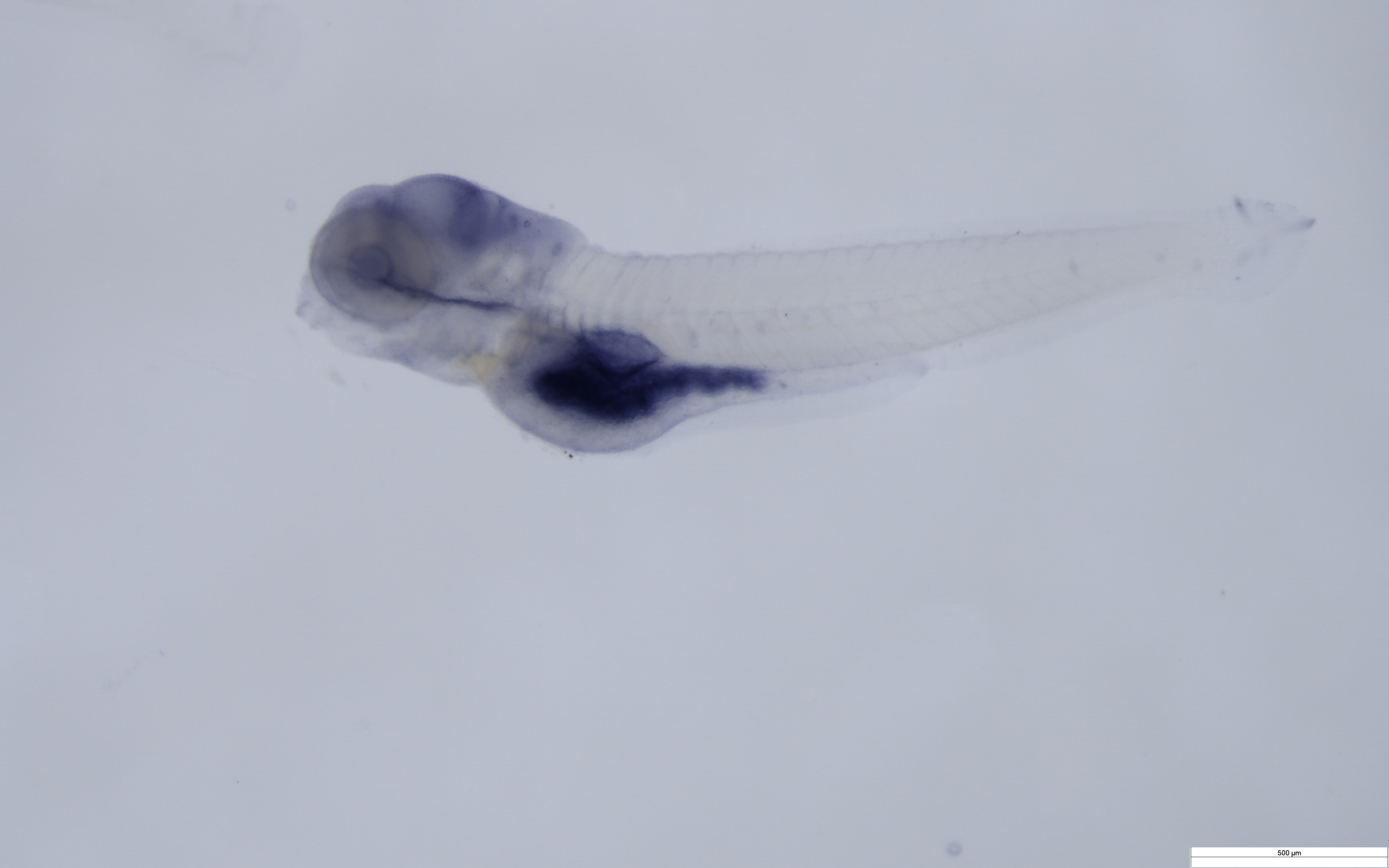

Supplement: Supplementary file 1 [file DataSheet3.ZIP › fig3/tu ifabp 4d 3.jpg]

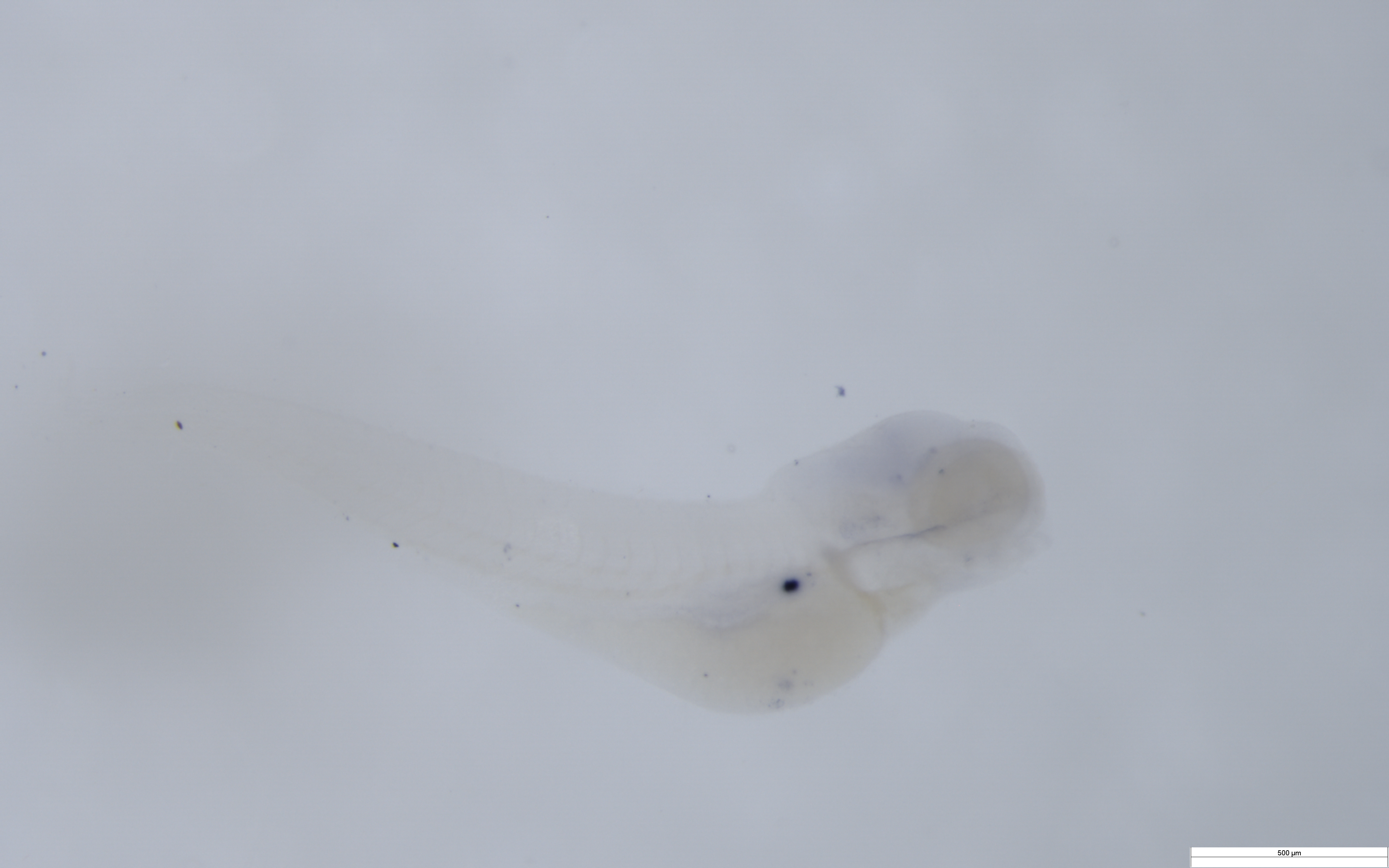

Supplement: Supplementary file 1 [file DataSheet3.ZIP › fig3/tu ins 4d 3.jpg]

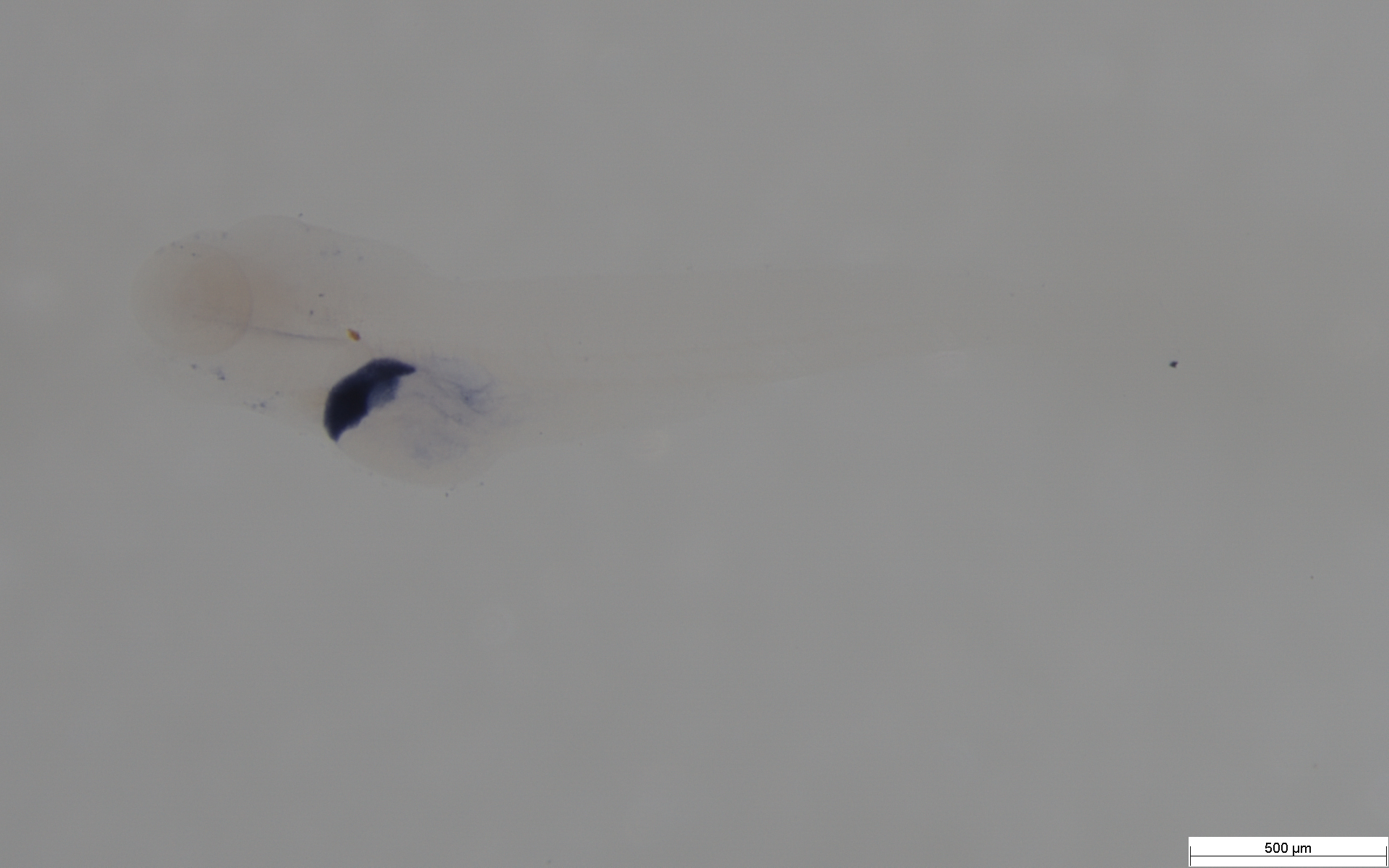

Supplement: Supplementary file 1 [file DataSheet3.ZIP › fig3/TU lfabp 4d e1_ch00.jpg]

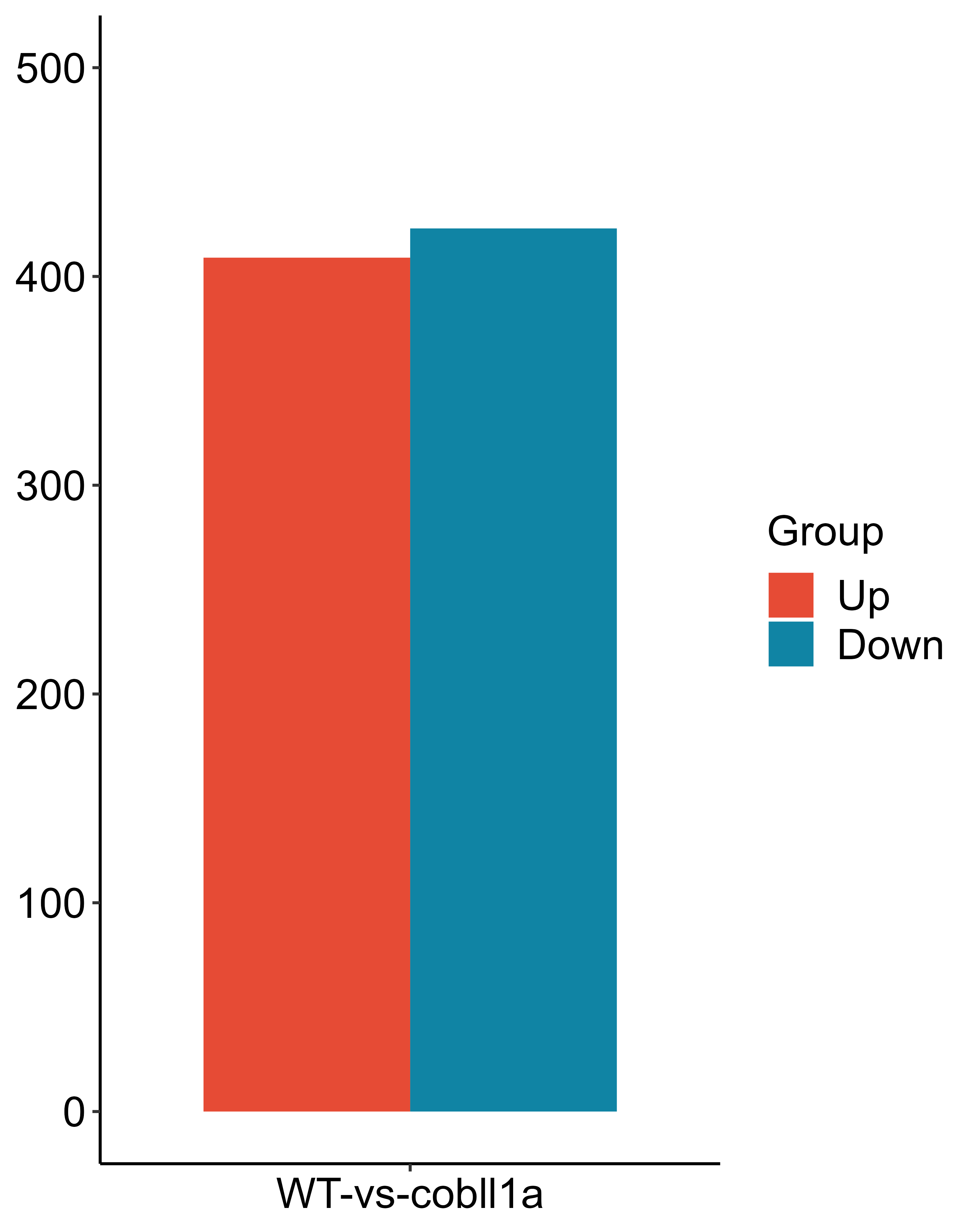

Supplement: Supplementary file 1 [file DataSheet3.ZIP › fig4/bar.png]

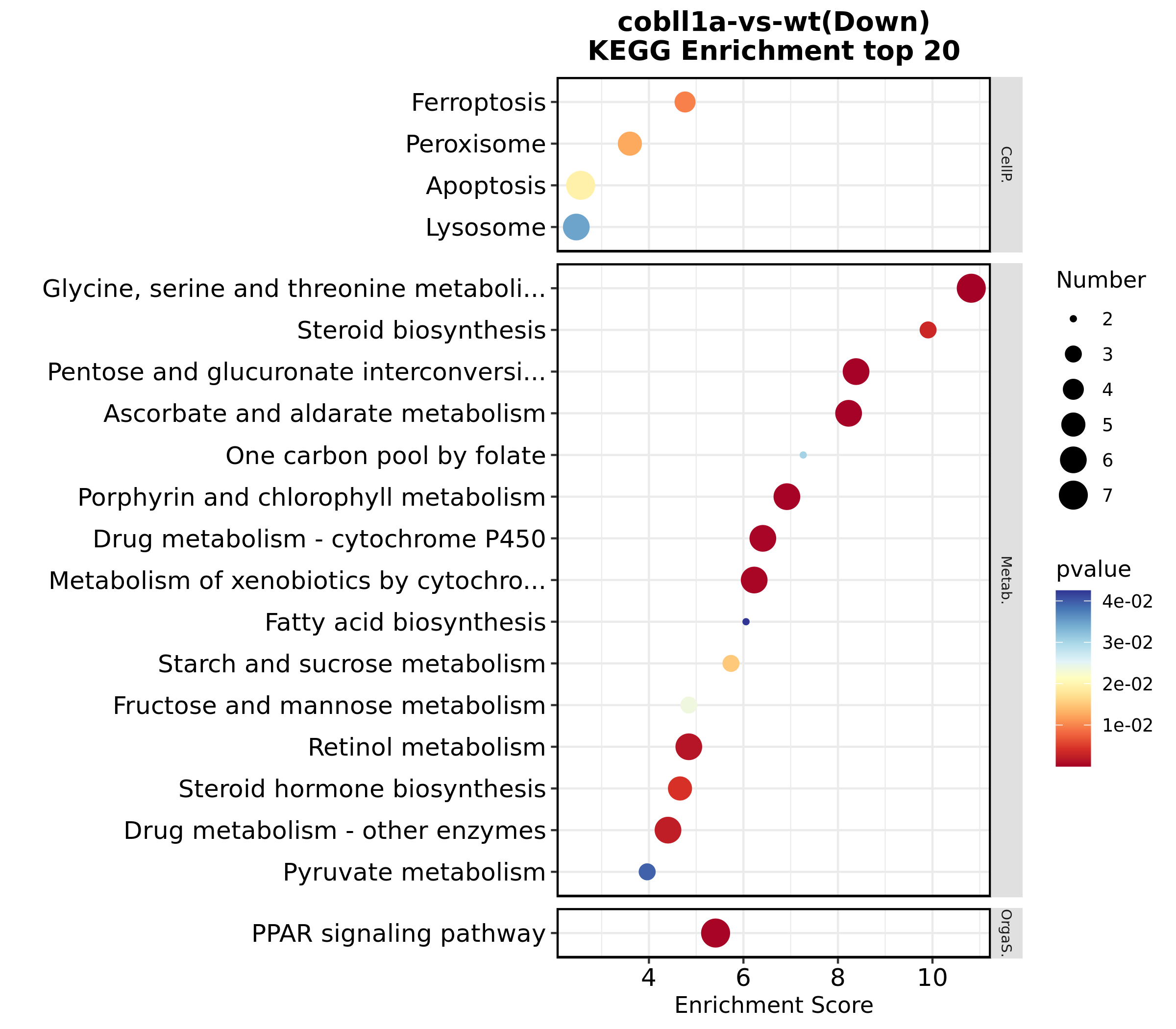

Supplement: Supplementary file 1 [file DataSheet3.ZIP › fig4/KEGG.top.Down.png]

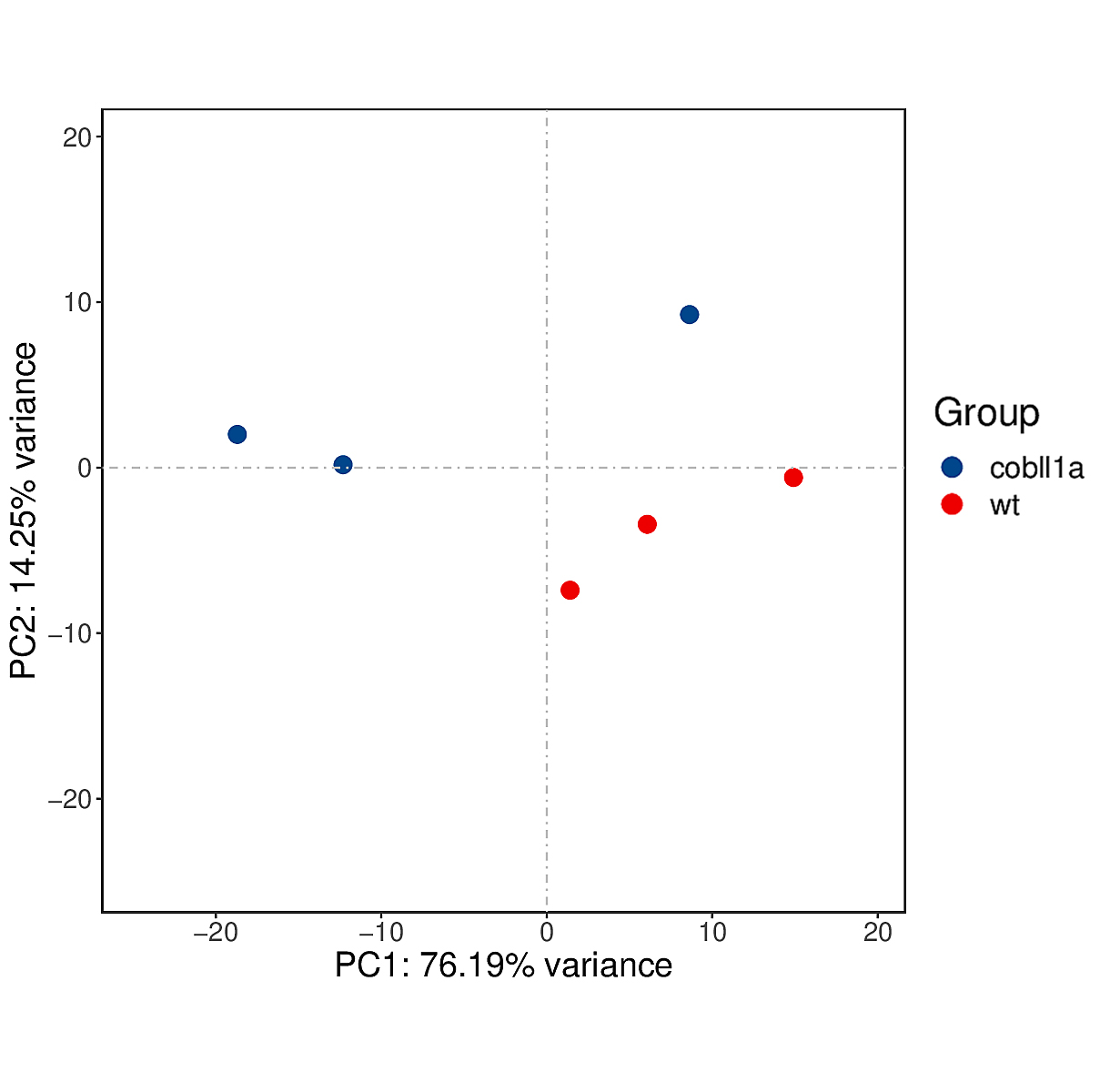

Supplement: Supplementary file 1 [file DataSheet3.ZIP › fig4/PCA.jpg]

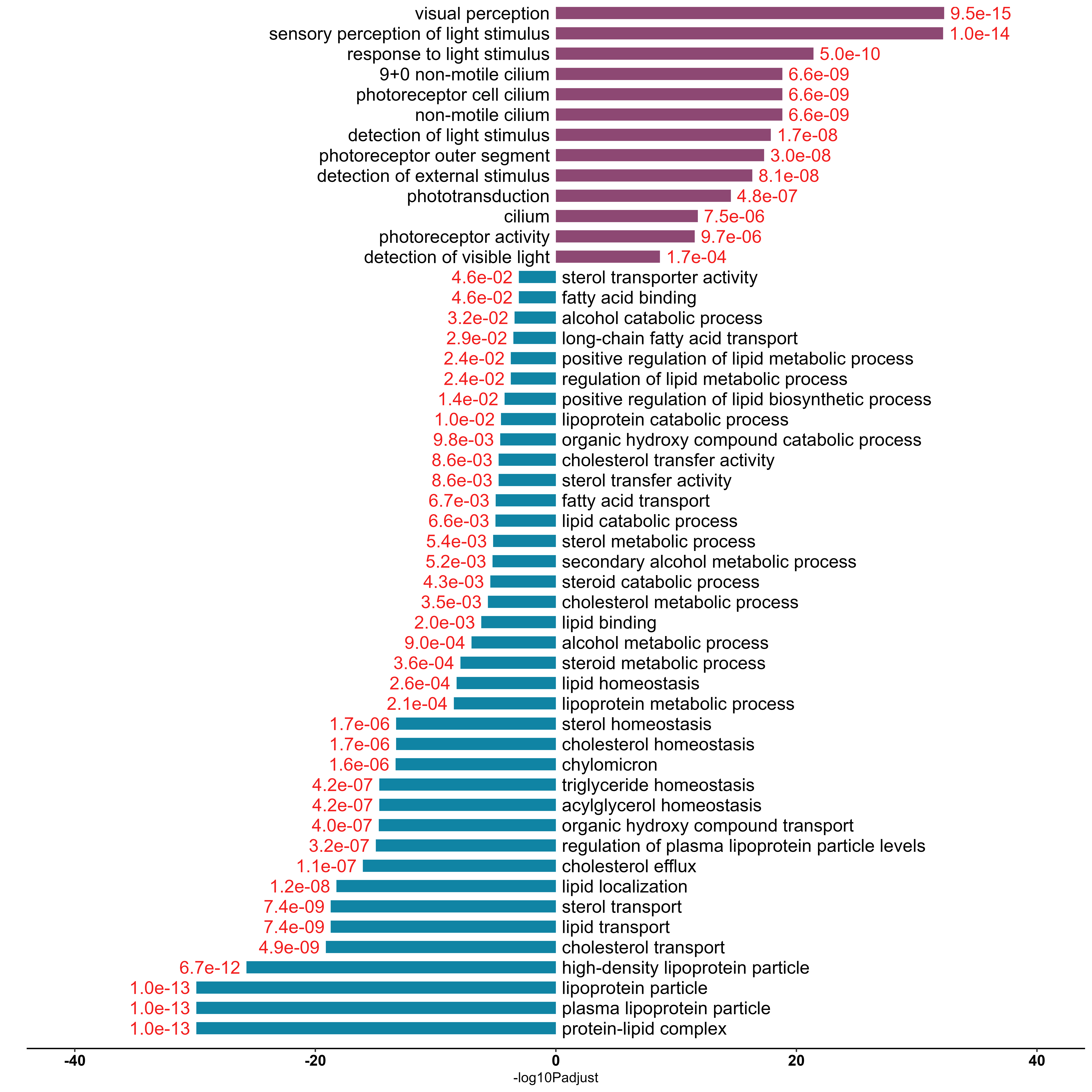

Supplement: Supplementary file 1 [file DataSheet3.ZIP › fig4/plot.png]

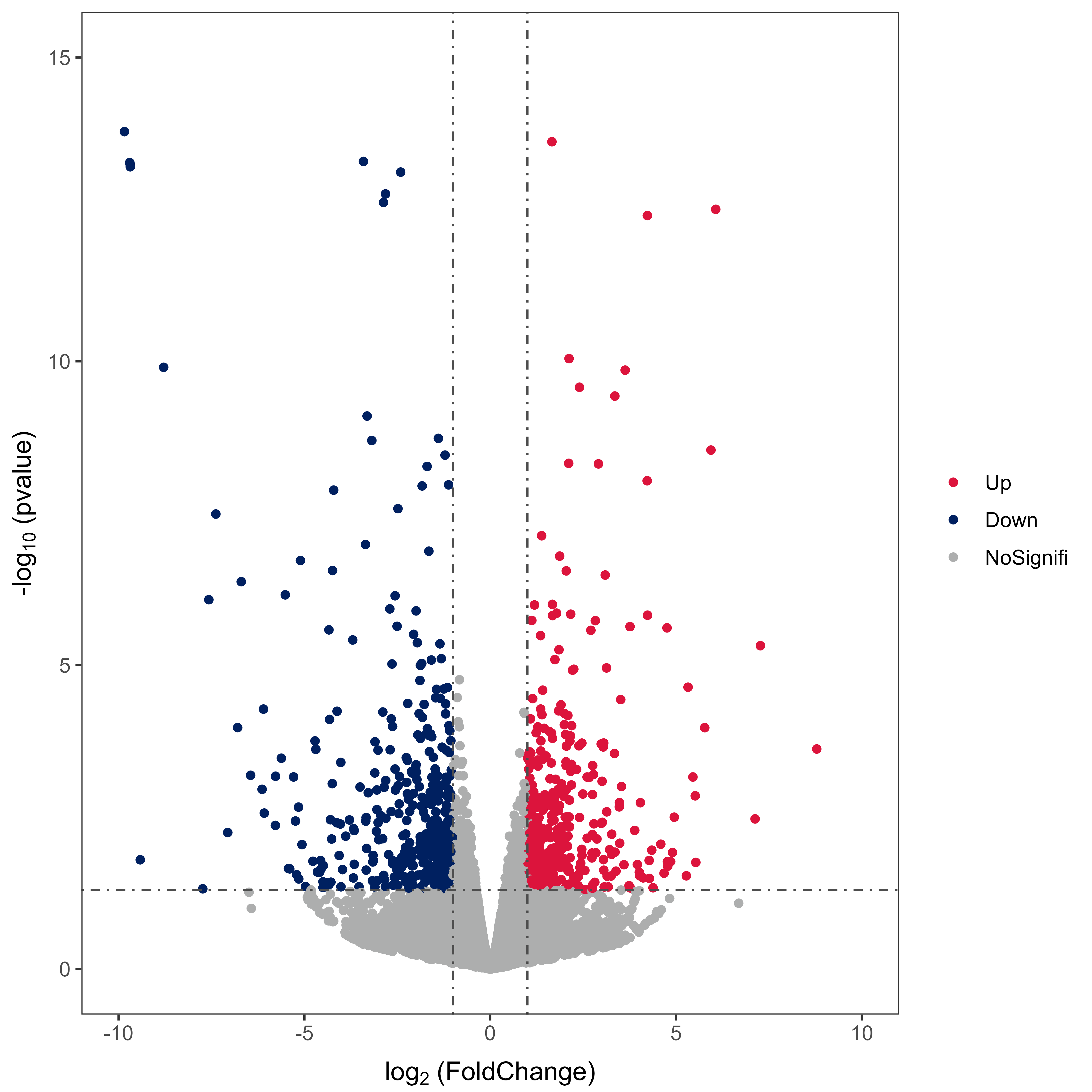

Supplement: Supplementary file 1 [file DataSheet3.ZIP › fig4/volcano.png]

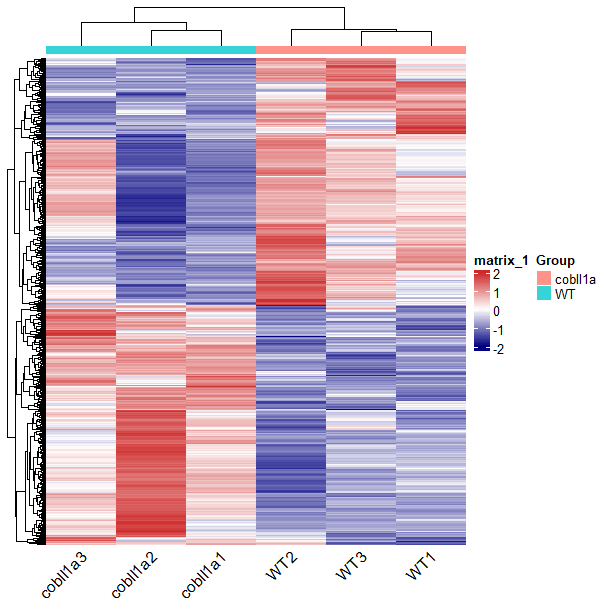

Supplement: Supplementary file 1 [file DataSheet3.ZIP › fig4/╚╚═╝.png]

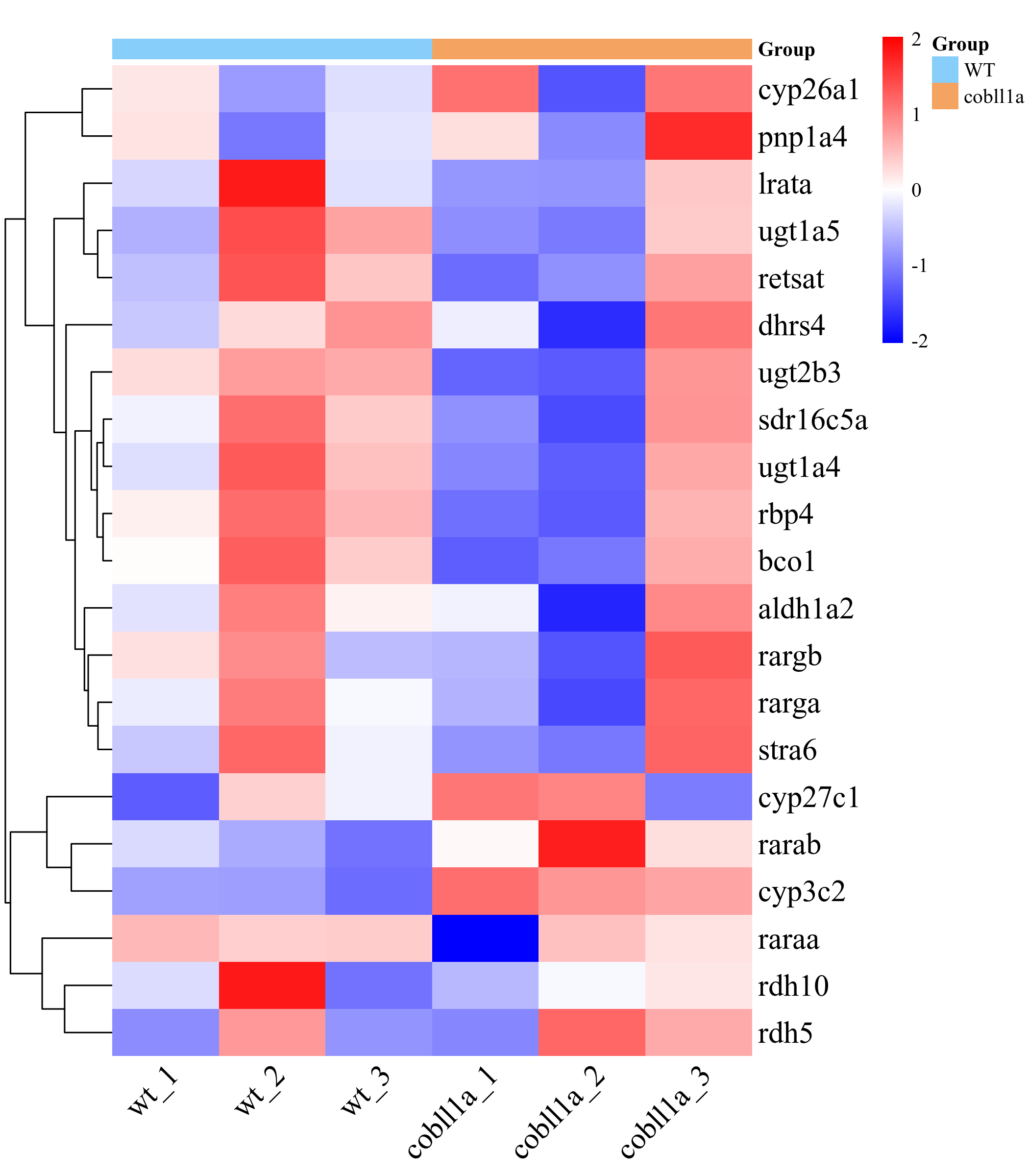

Supplement: Supplementary file 3 [file DataSheet4.ZIP › fig5/cluster-heatmap-1.png]

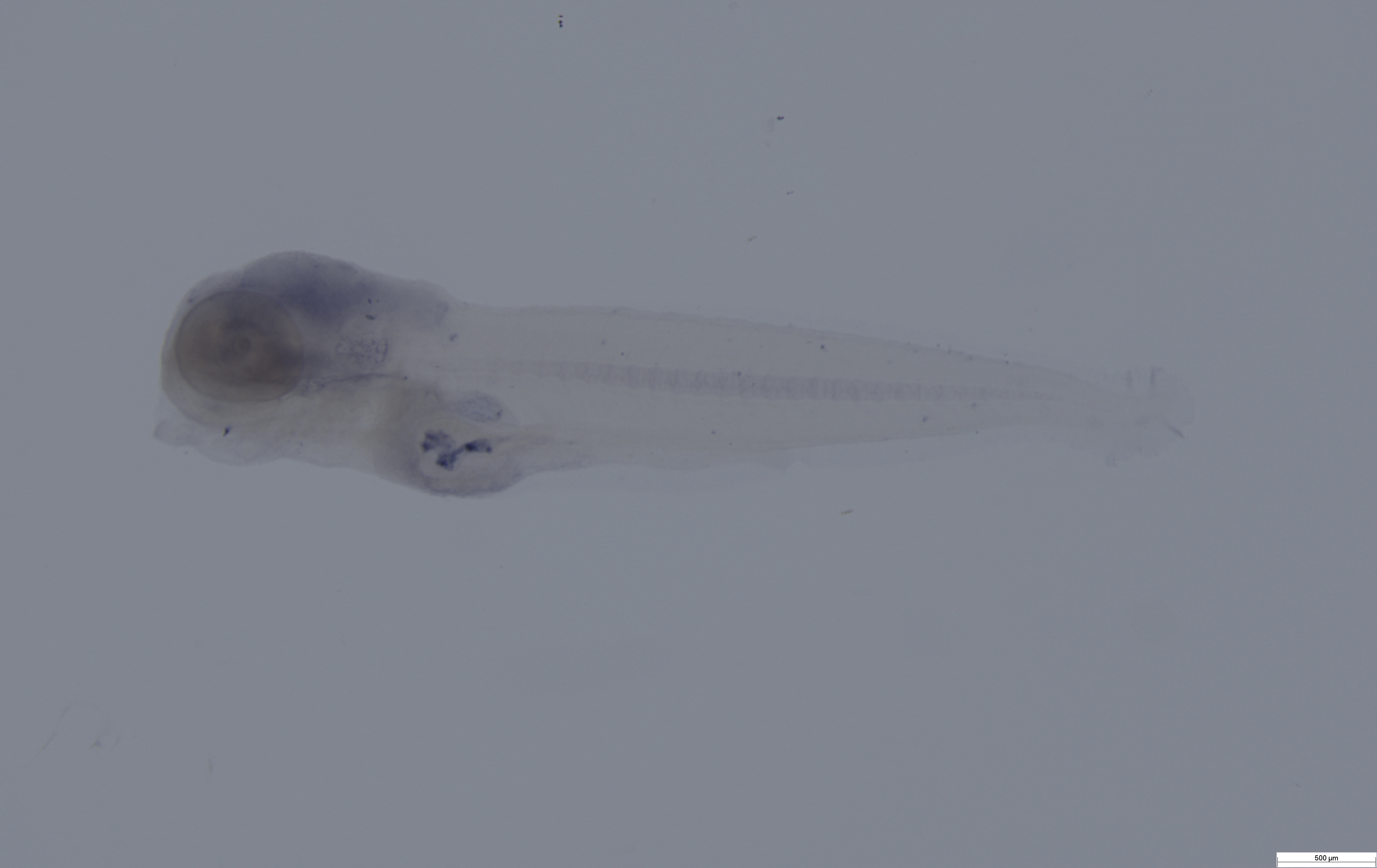

Supplement: Supplementary file 3 [file DataSheet4.ZIP › fig5/coa mut cyp26a1 4d e3 3.2X.jpg]

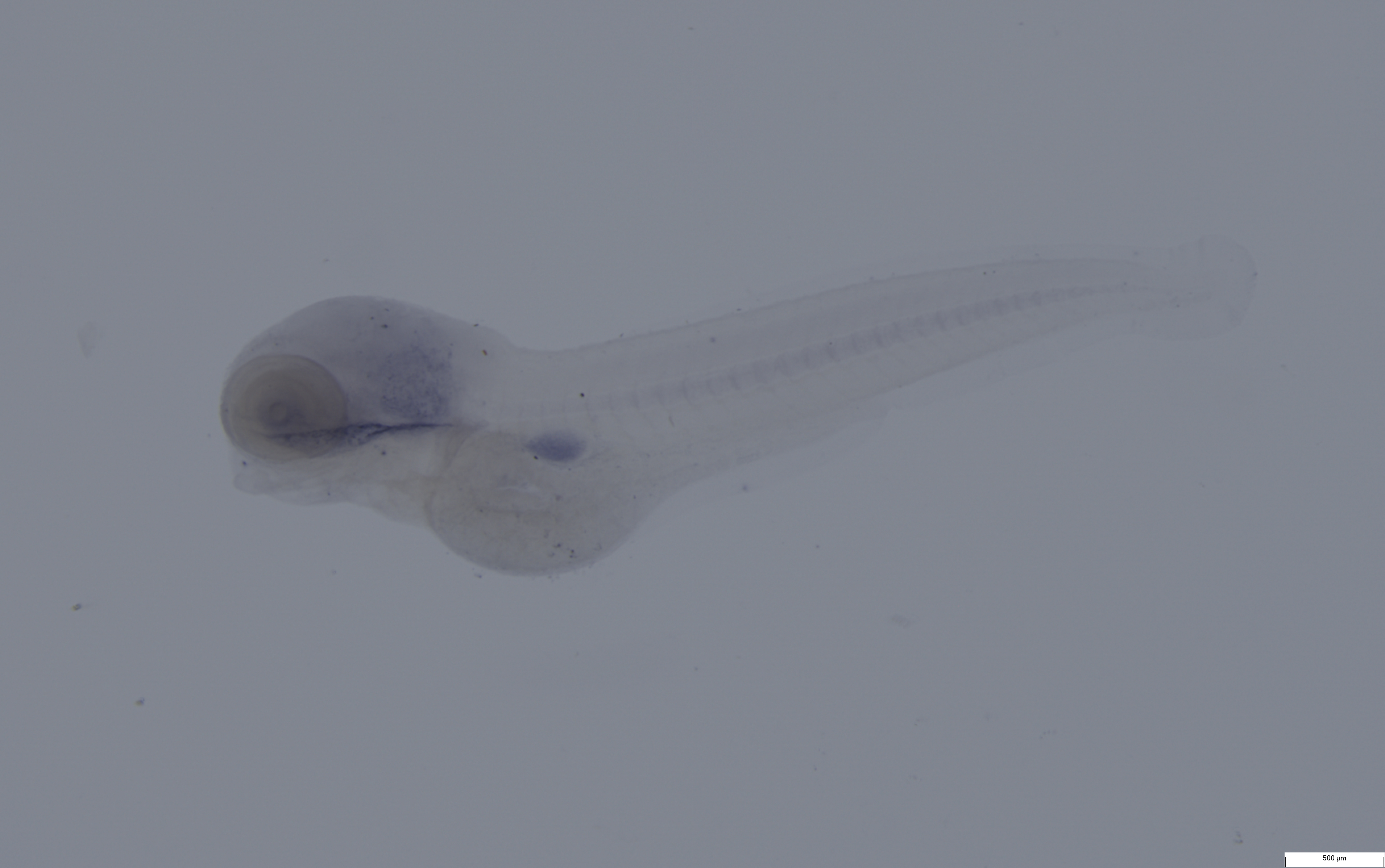

Supplement: Supplementary file 3 [file DataSheet4.ZIP › fig5/coa mut rdh10 4d e1 3.2X.jpg]

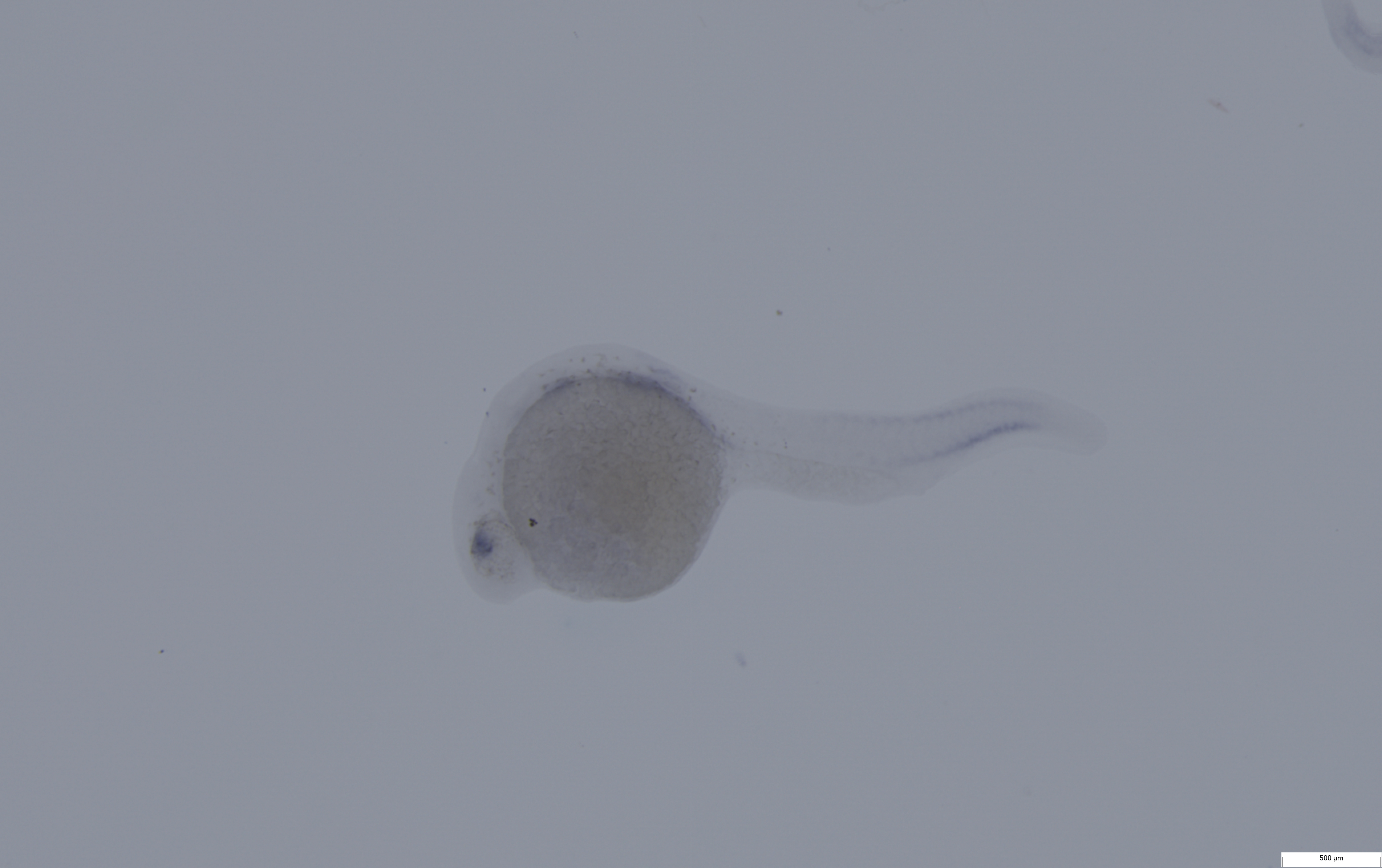

Supplement: Supplementary file 3 [file DataSheet4.ZIP › fig5/coa-mut e1 aldh1a2 3.2x 24h.jpg]

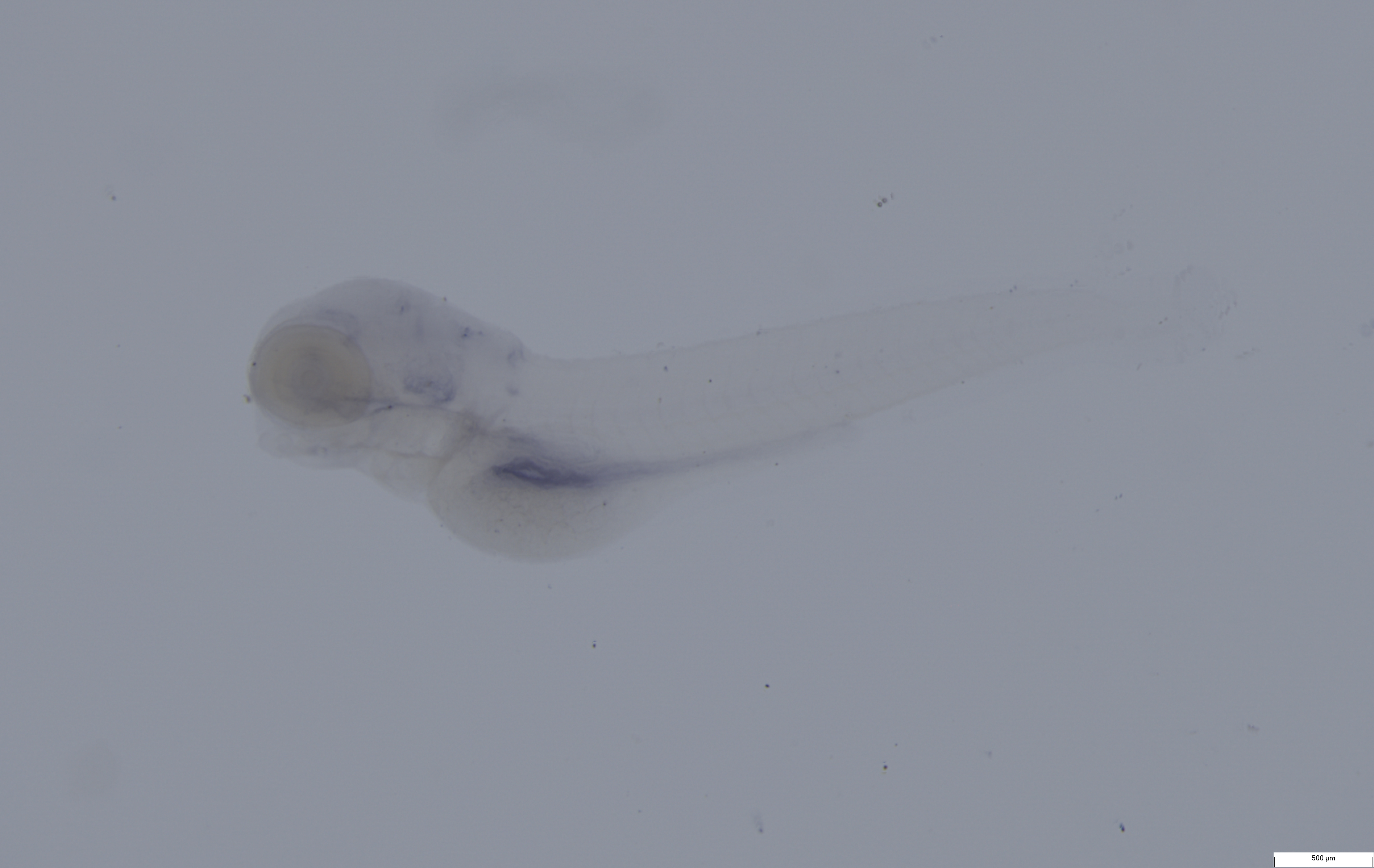

Supplement: Supplementary file 3 [file DataSheet4.ZIP › fig5/coa-mut e1 aldh1a2 3.2x 4d.jpg]

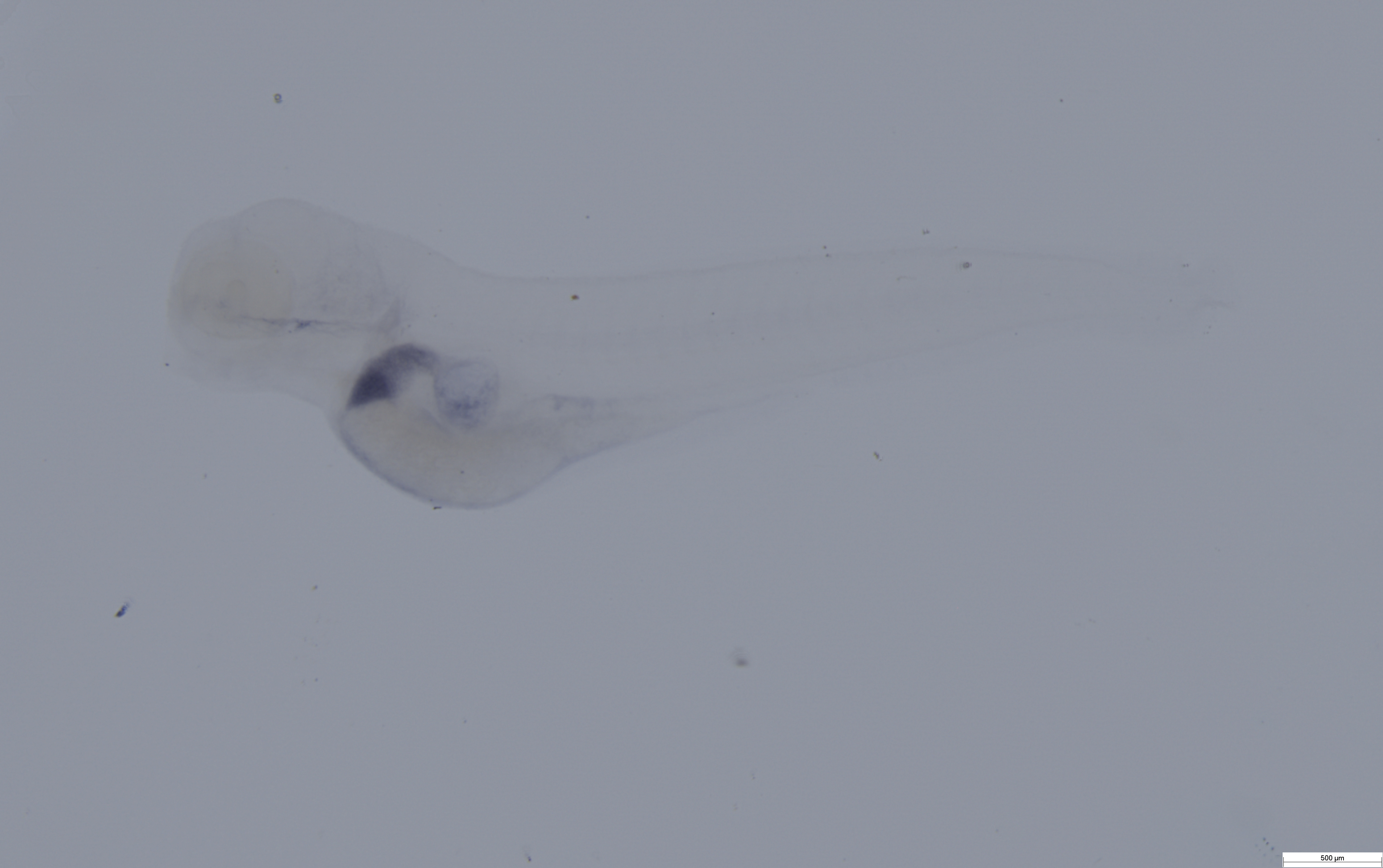

Supplement: Supplementary file 3 [file DataSheet4.ZIP › fig5/coa-mut e2 rbp4 3.2X 4d.jpg]

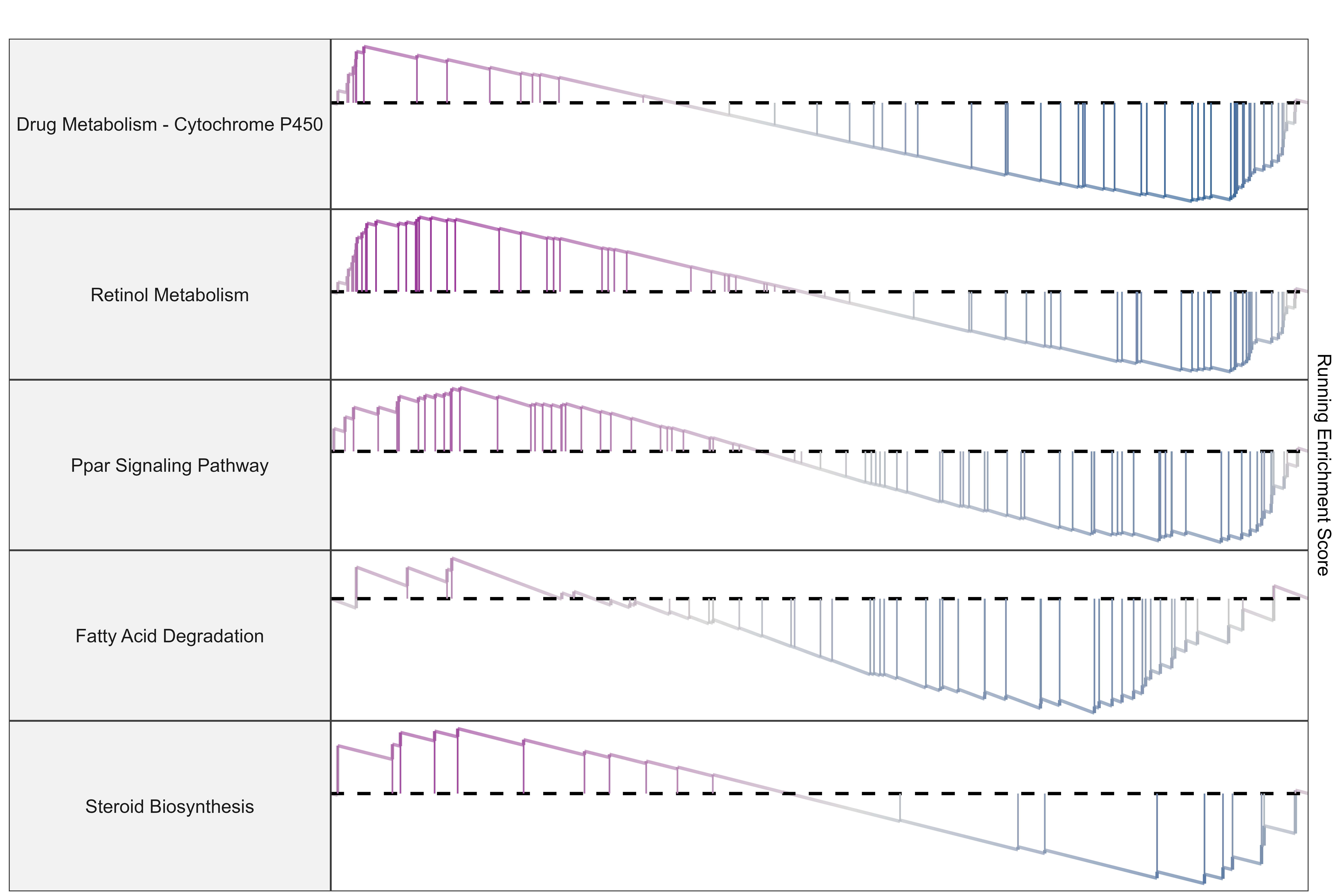

Supplement: Supplementary file 3 [file DataSheet4.ZIP › fig5/gsea.png]

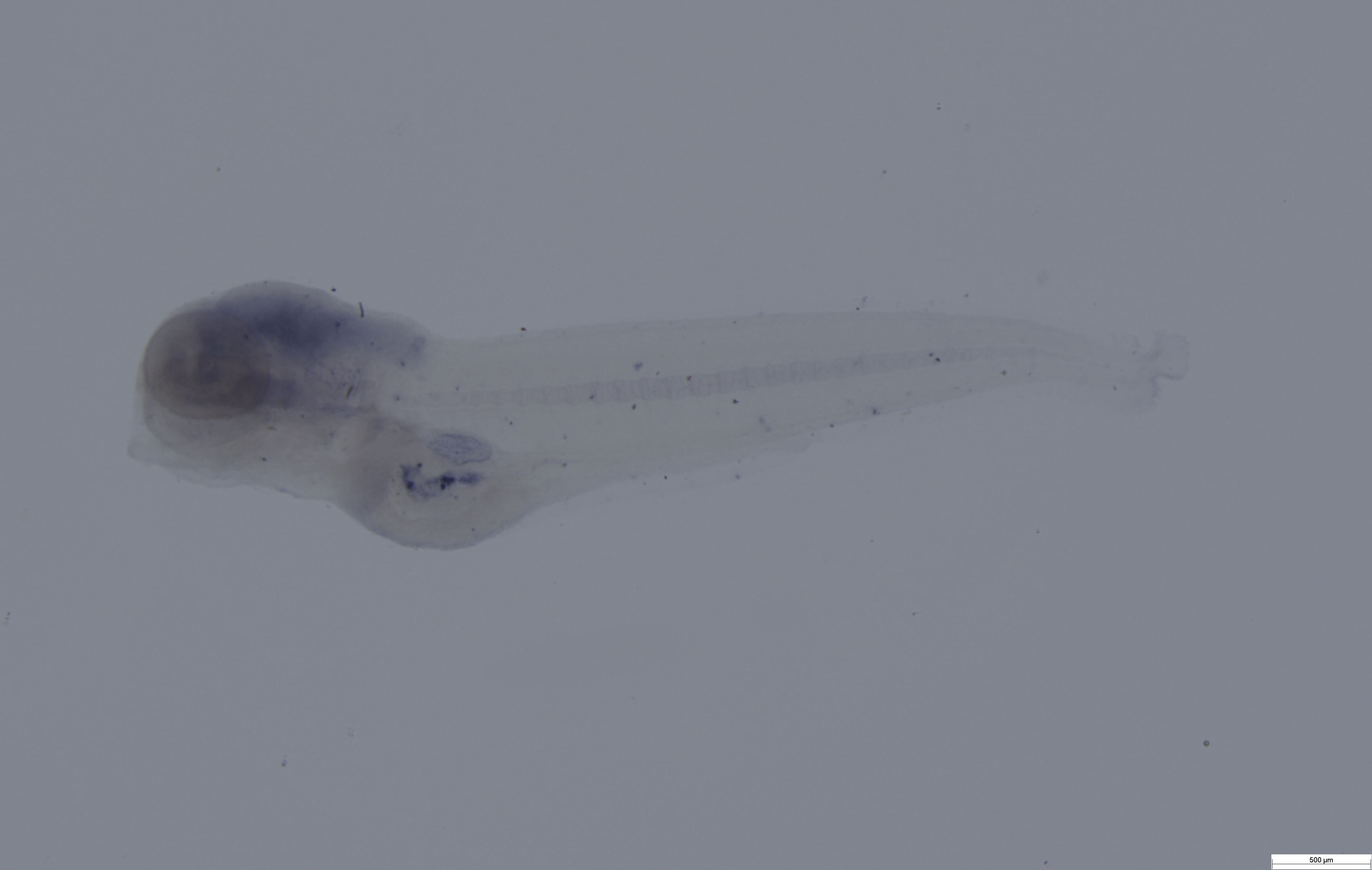

Supplement: Supplementary file 3 [file DataSheet4.ZIP › fig5/tu cyp26a1 4d e4 3.2X.jpg]

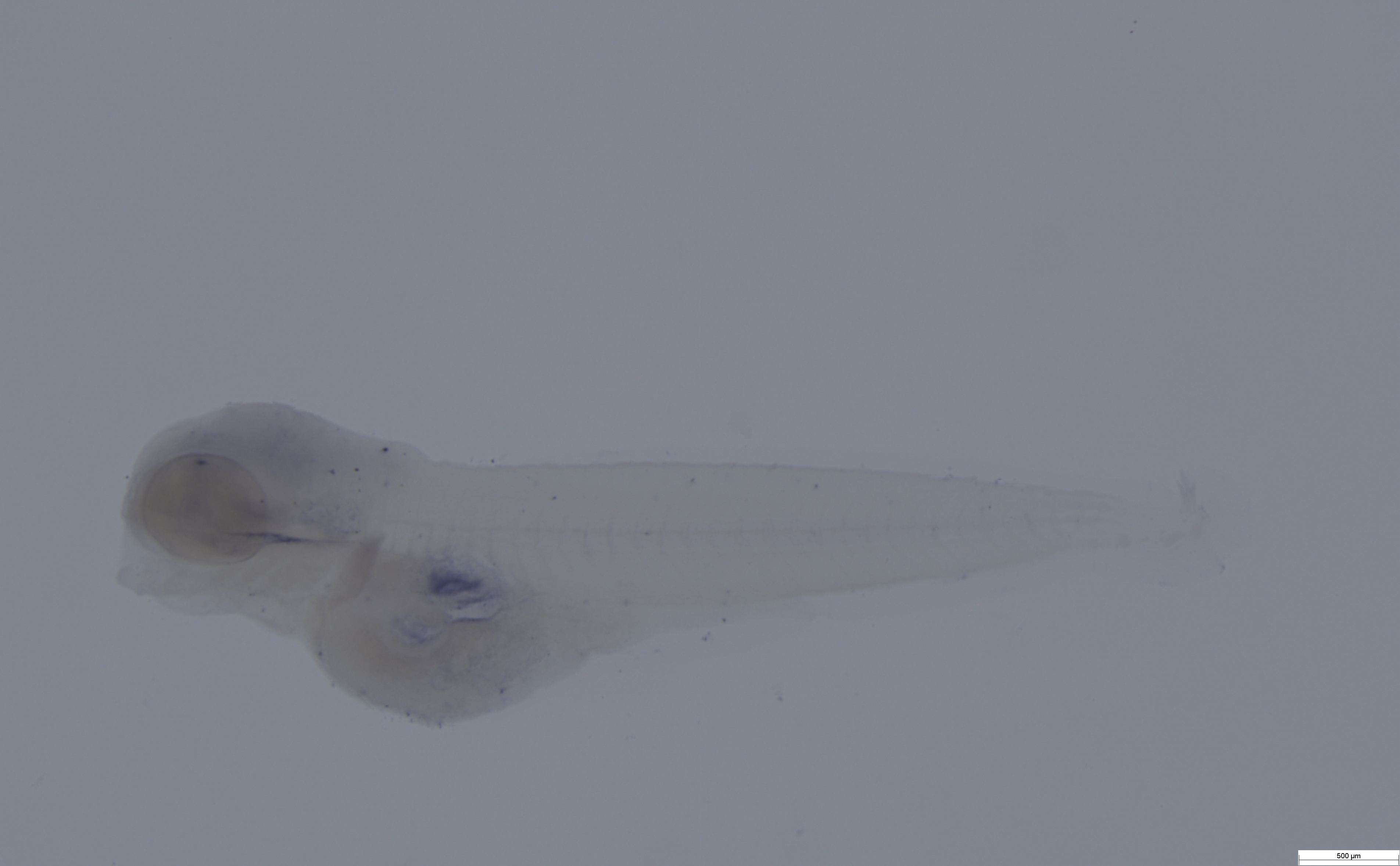

Supplement: Supplementary file 3 [file DataSheet4.ZIP › fig5/tu rdh10 4d e1 3.2X.jpg]

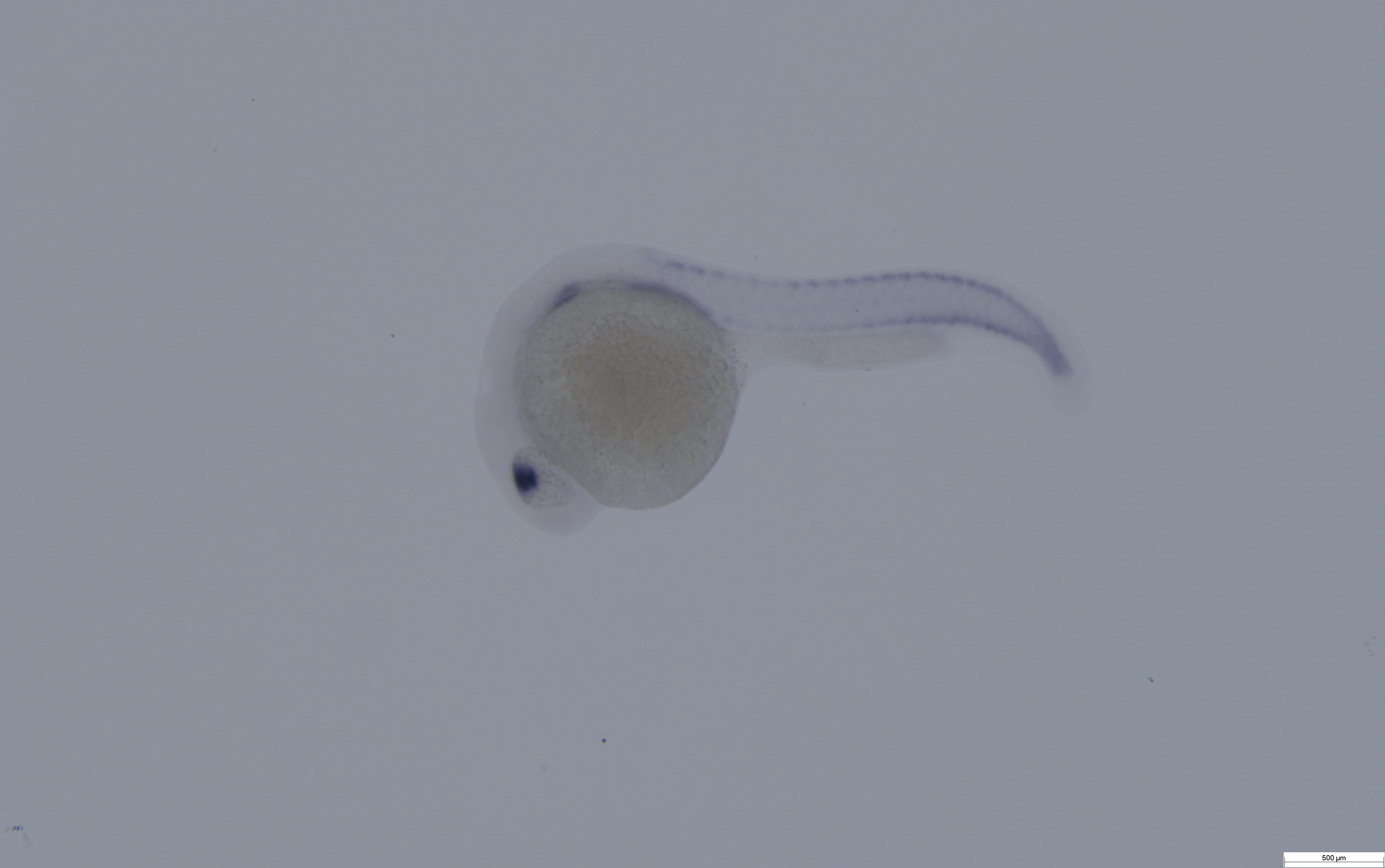

Supplement: Supplementary file 3 [file DataSheet4.ZIP › fig5/tu e1 aldh1a2 3.2x 24h.jpg]

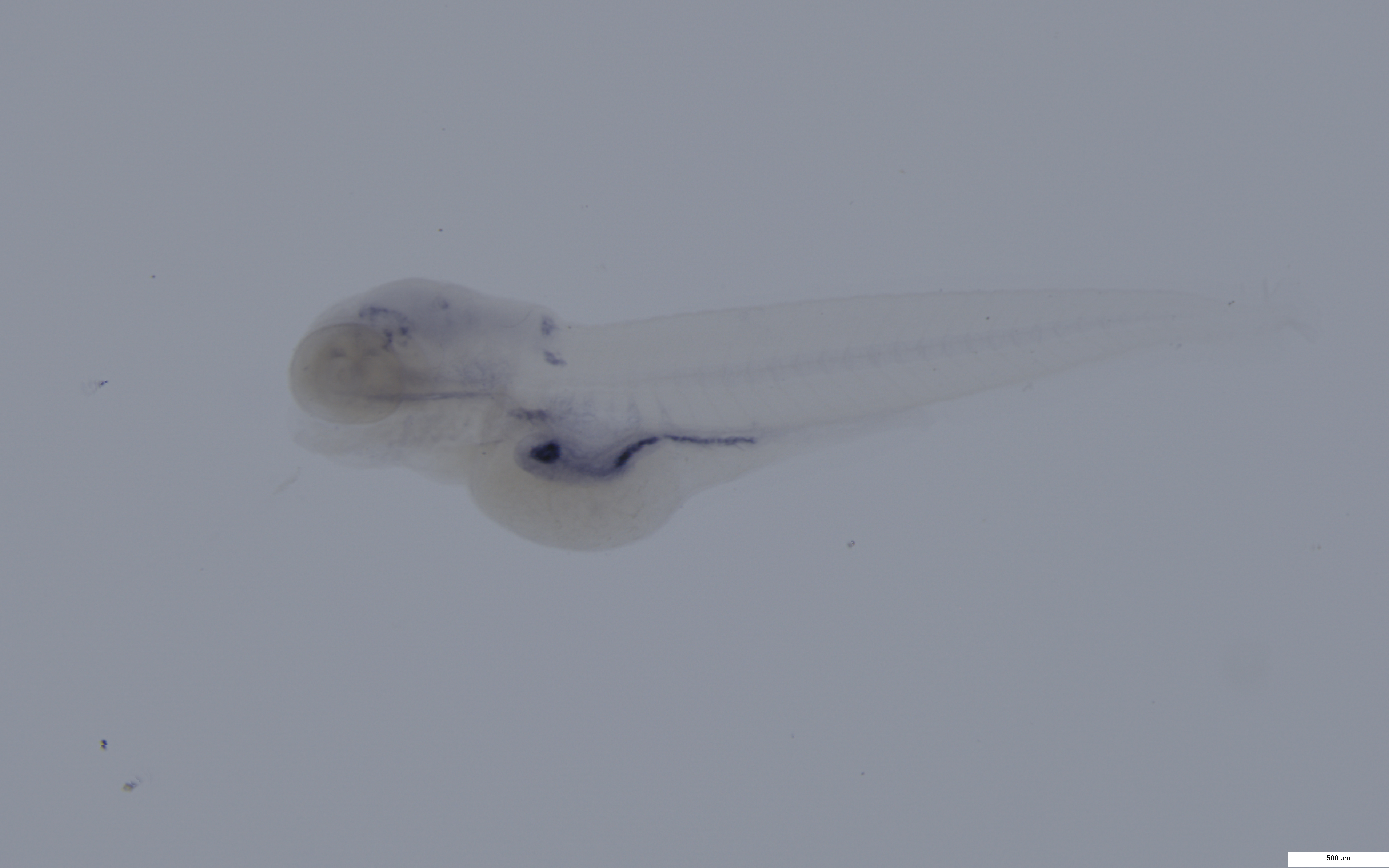

Supplement: Supplementary file 3 [file DataSheet4.ZIP › fig5/tu e1 aldh1a2 3.2X 4d.jpg]

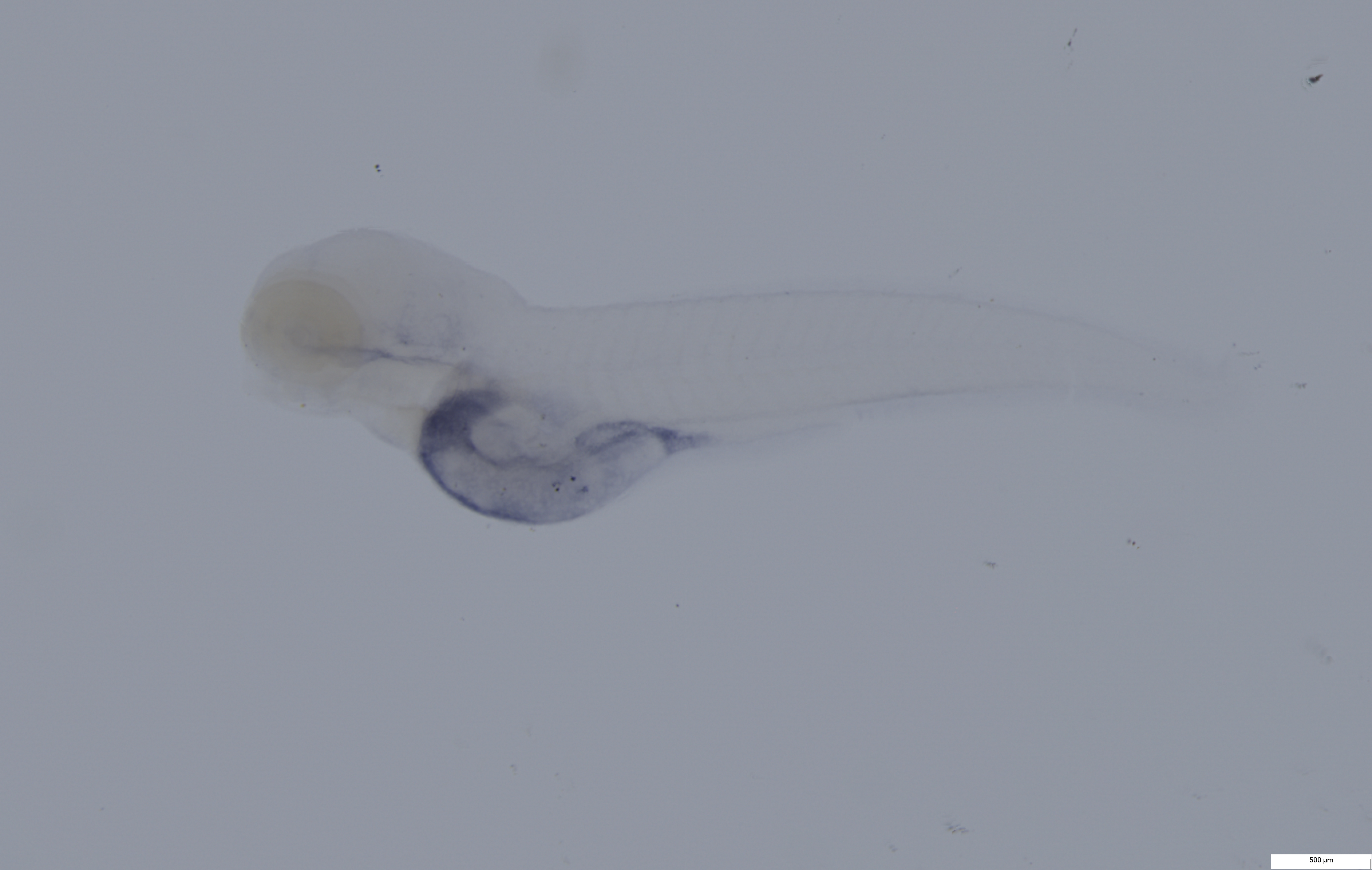

Supplement: Supplementary file 3 [file DataSheet4.ZIP › fig5/tu-con e3 rbp4 3.2x 4d.jpg]

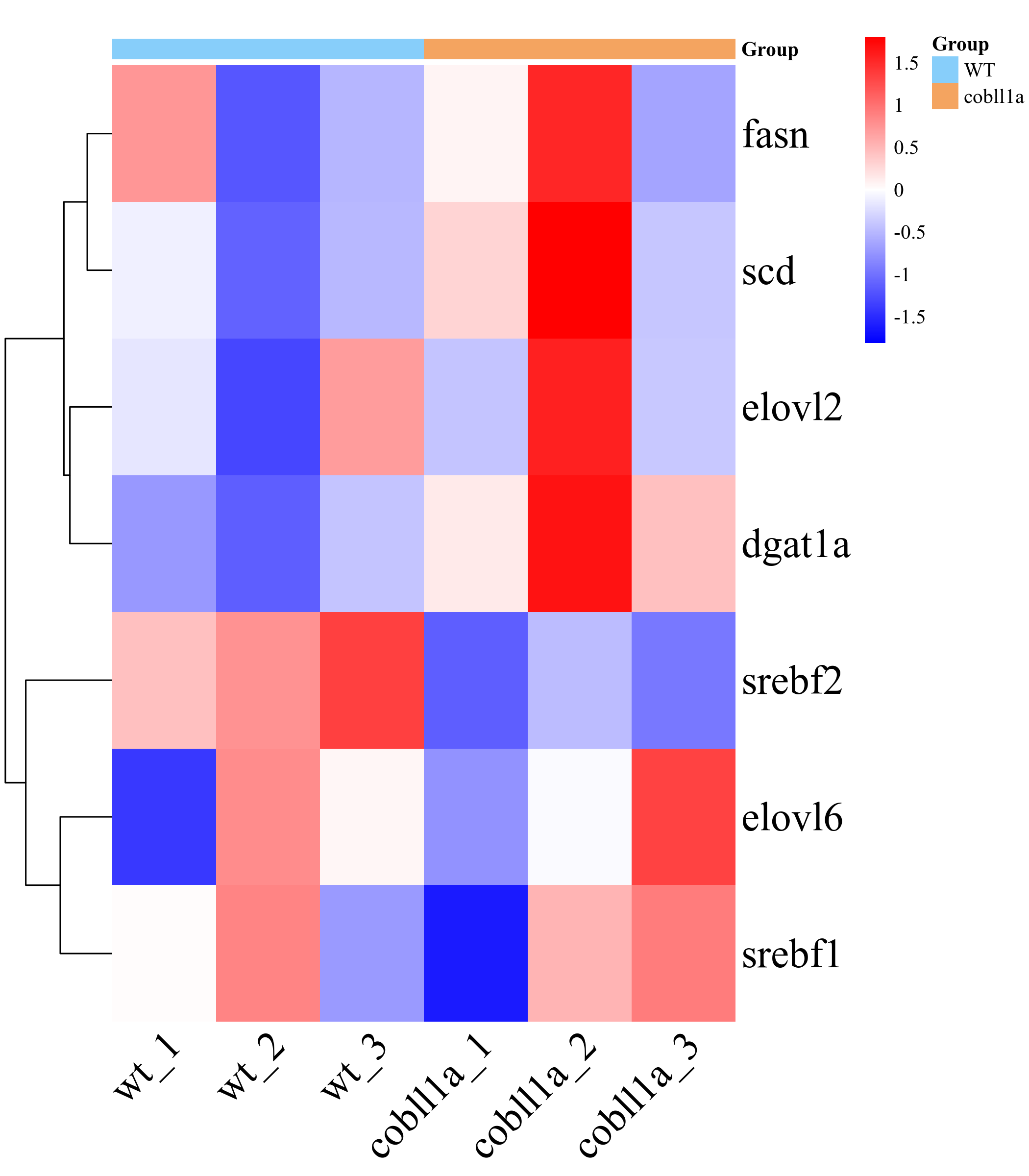

Supplement: Supplementary file 3 [file DataSheet4.ZIP › fig6/cluster-heatmap.png]

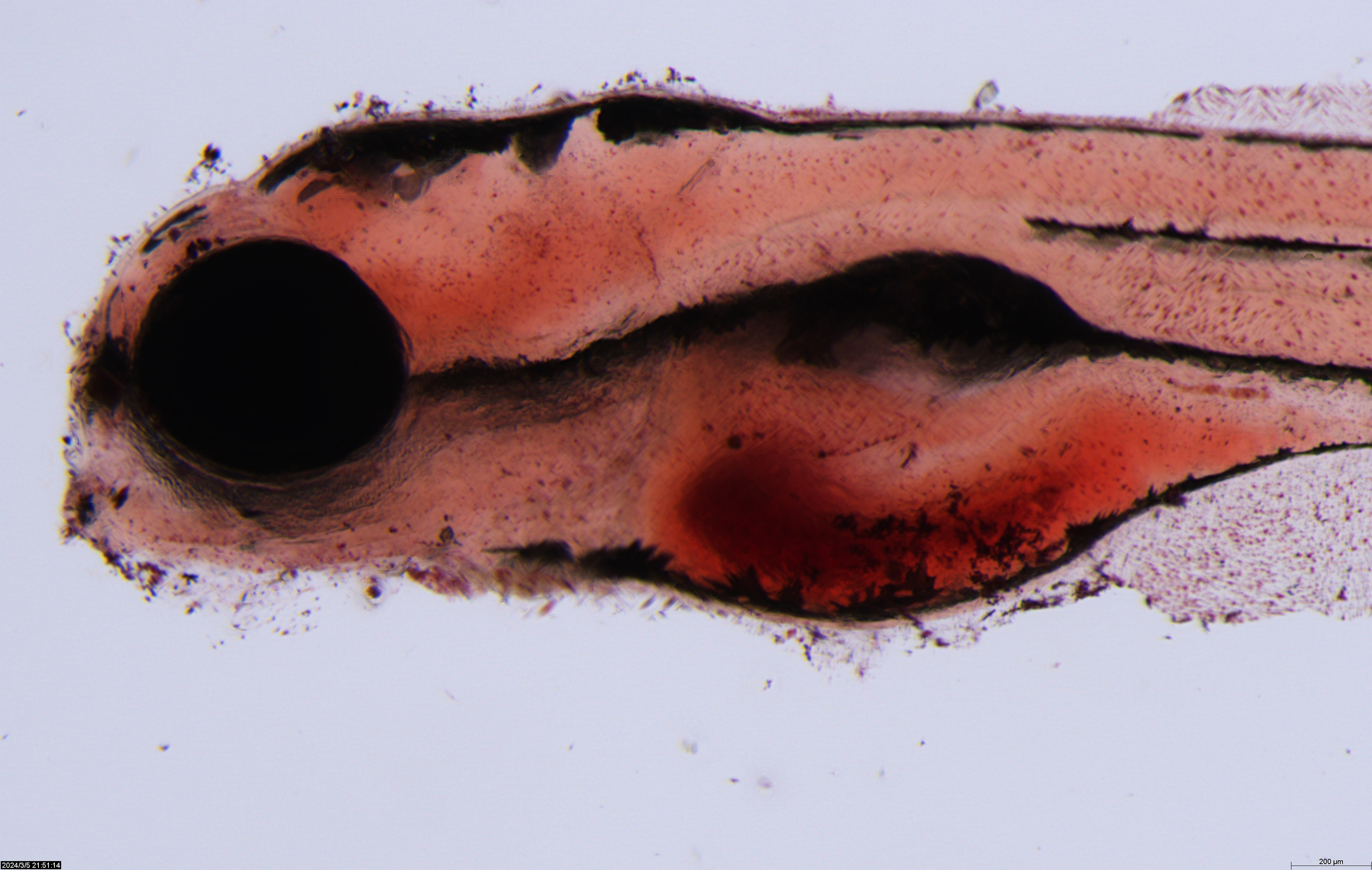

Supplement: Supplementary file 3 [file DataSheet4.ZIP › fig6/coa mut e1.jpg]

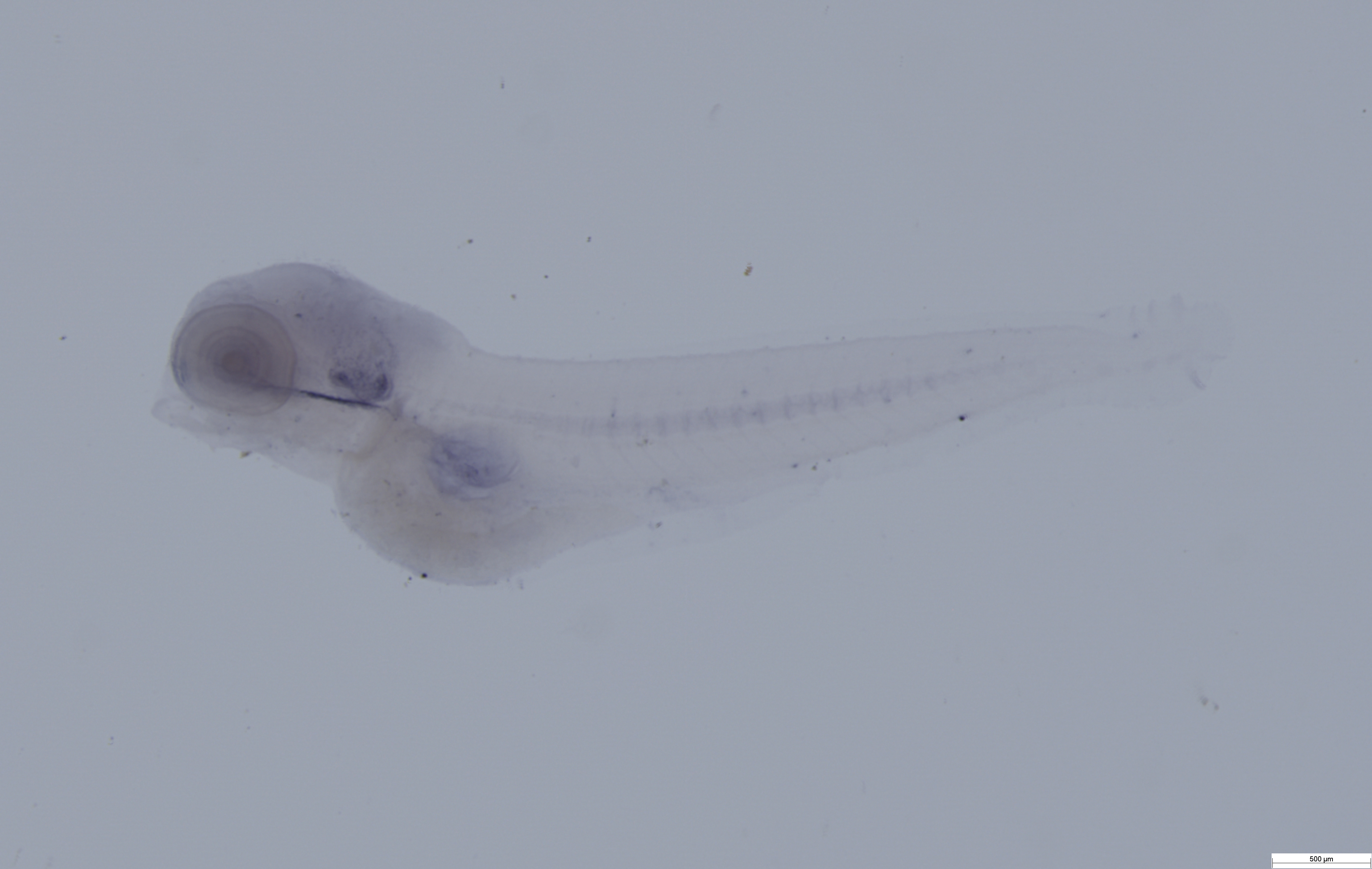

Supplement: Supplementary file 3 [file DataSheet4.ZIP › fig6/coa-mut e3 fasn 3.2x 4d.jpg]

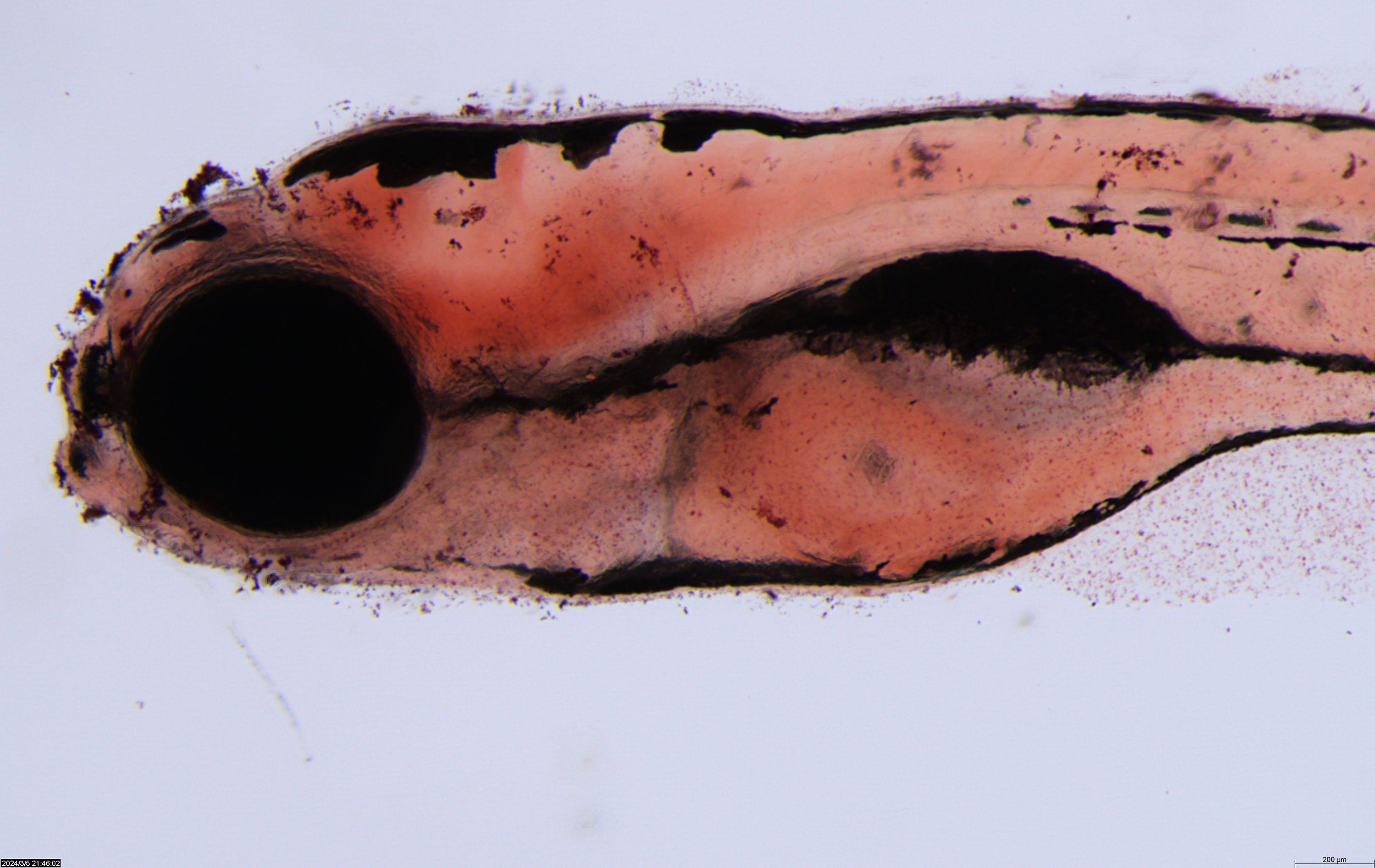

Supplement: Supplementary file 3 [file DataSheet4.ZIP › fig6/control e2 .jpg]

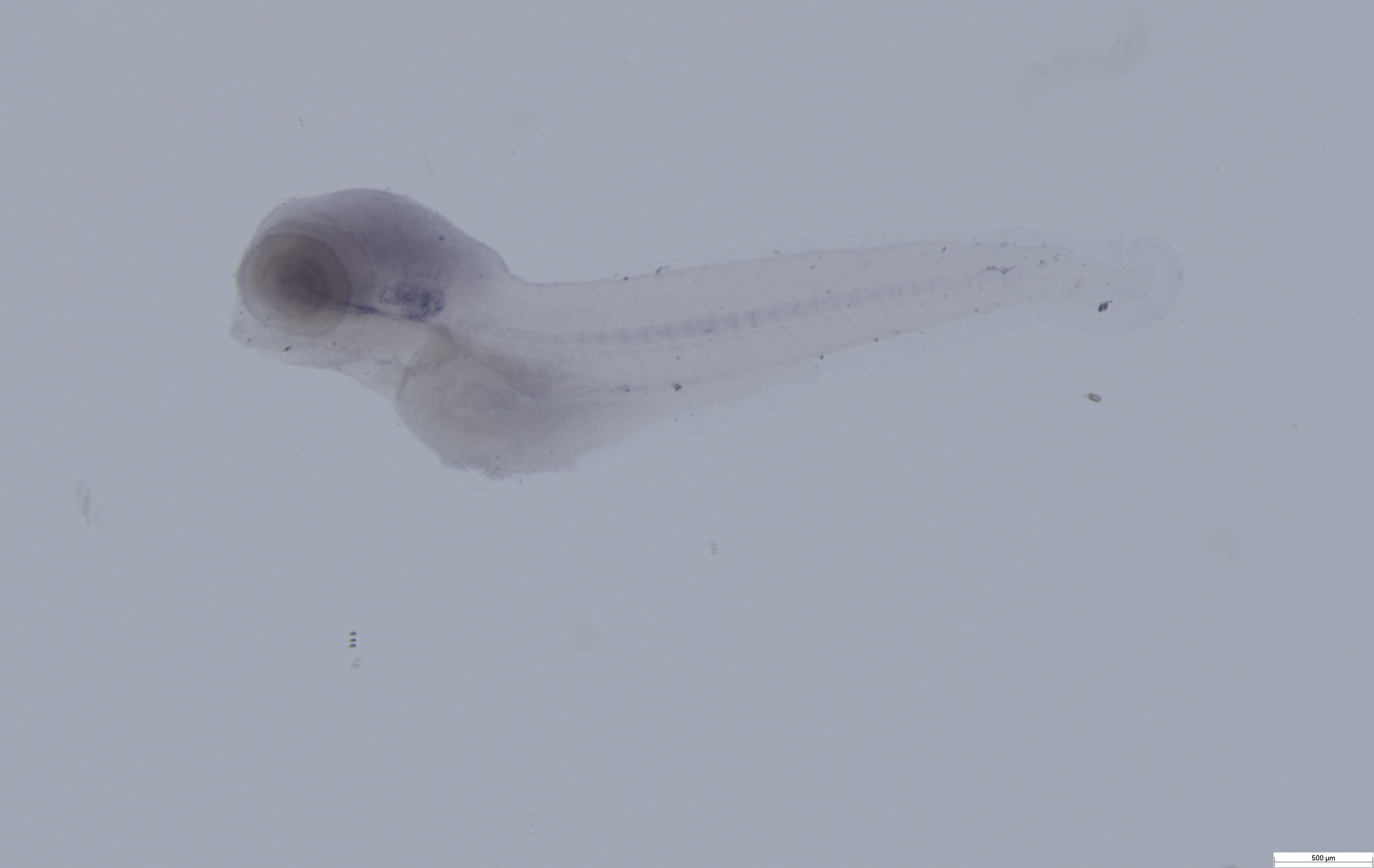

Supplement: Supplementary file 3 [file DataSheet4.ZIP › fig6/tu e1 fasn 3.2x 4d.jpg]

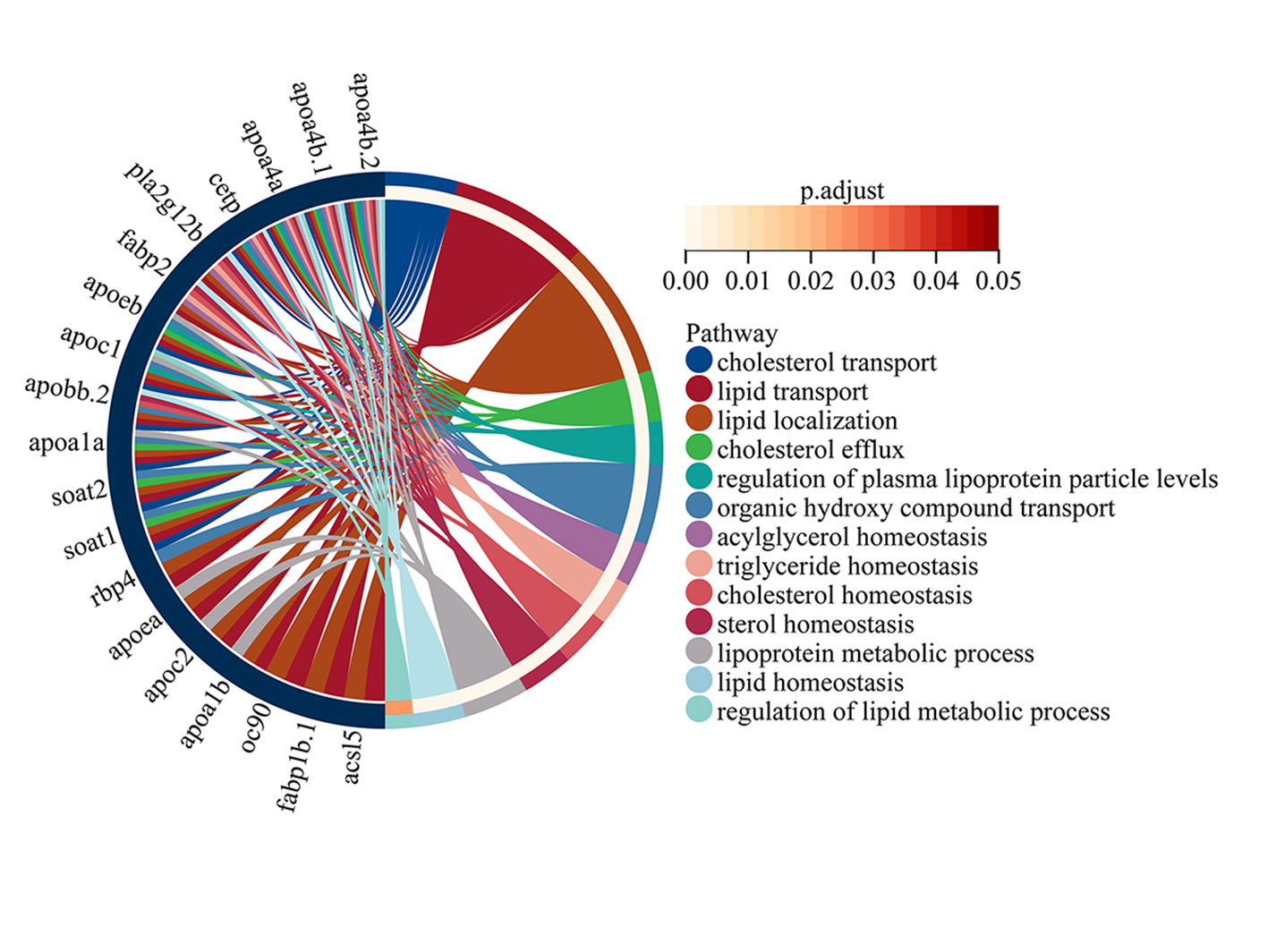

Supplement: Supplementary file 3 [file DataSheet4.ZIP › fig7/BP-01.jpg]

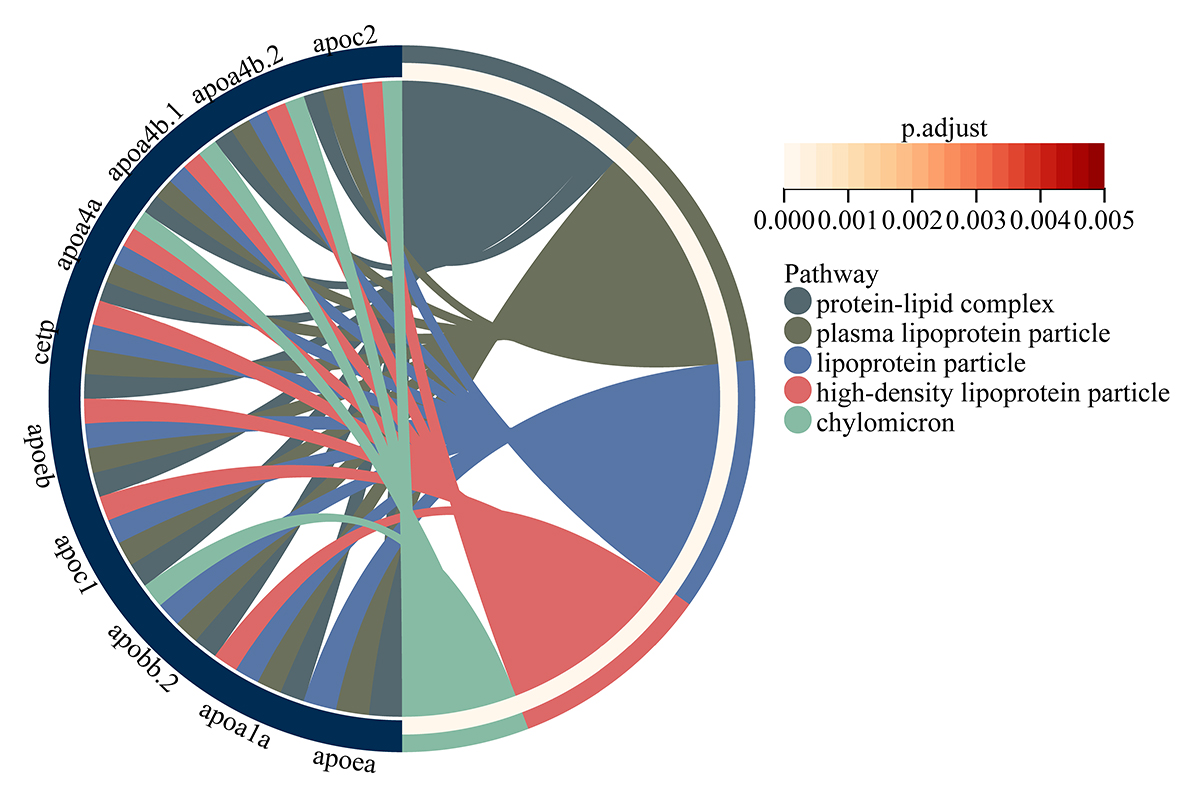

Supplement: Supplementary file 3 [file DataSheet4.ZIP › fig7/CC-01-01.jpg]

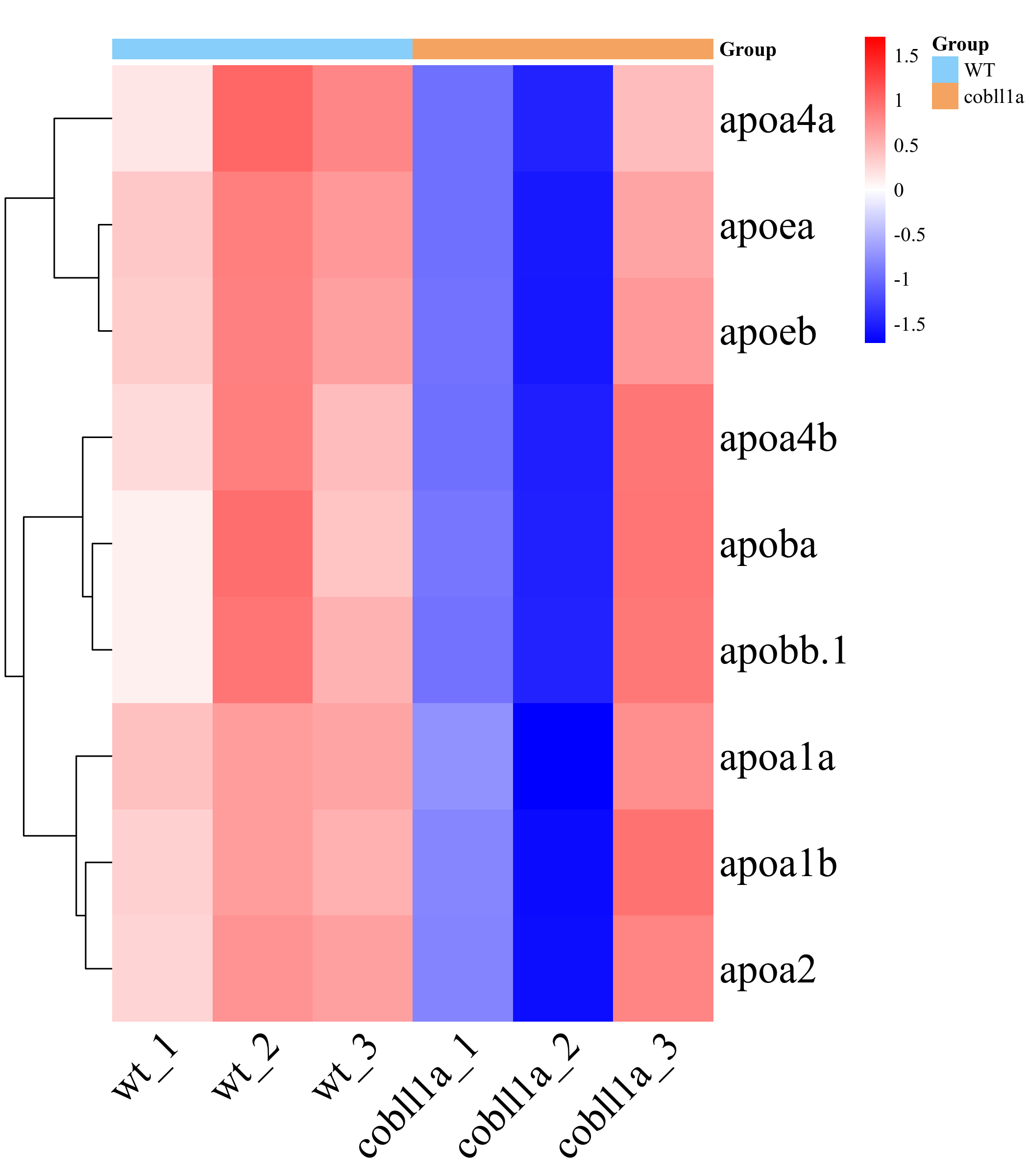

Supplement: Supplementary file 3 [file DataSheet4.ZIP › fig7/cluster-heatmap.png]

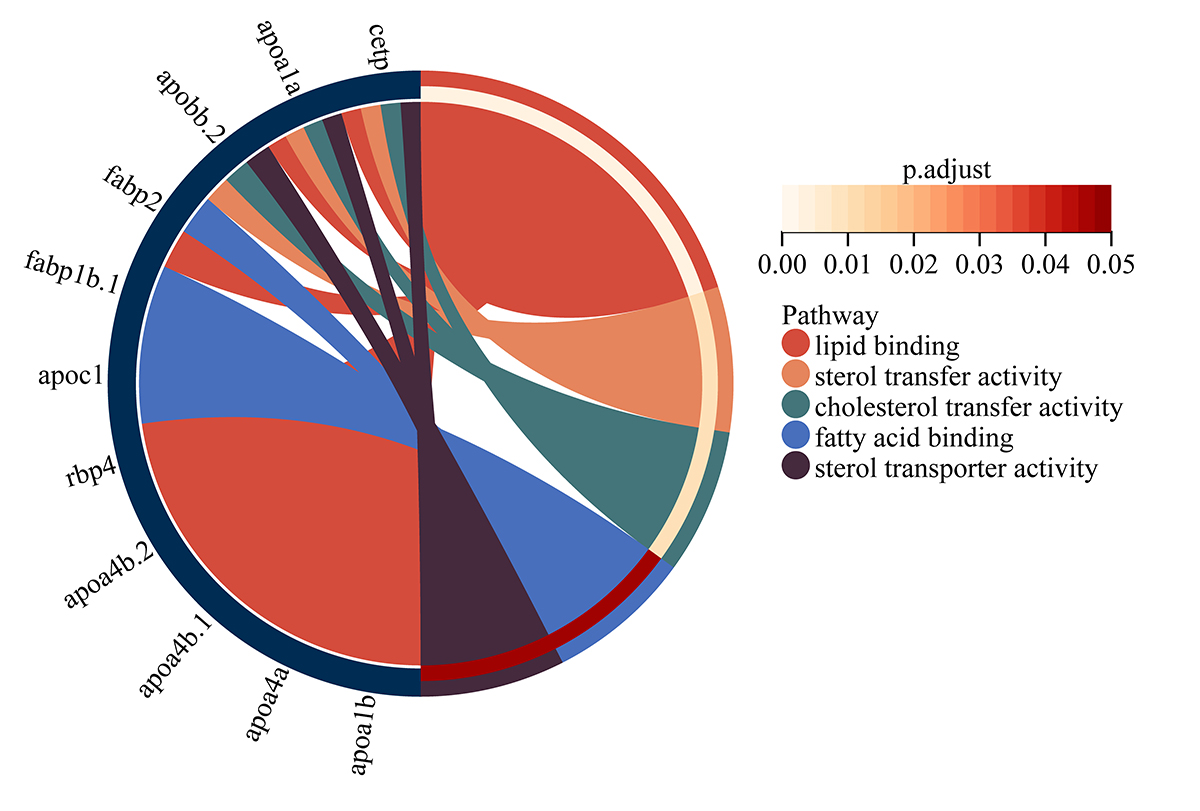

Supplement: Supplementary file 3 [file DataSheet4.ZIP › fig7/MF-01.jpg]

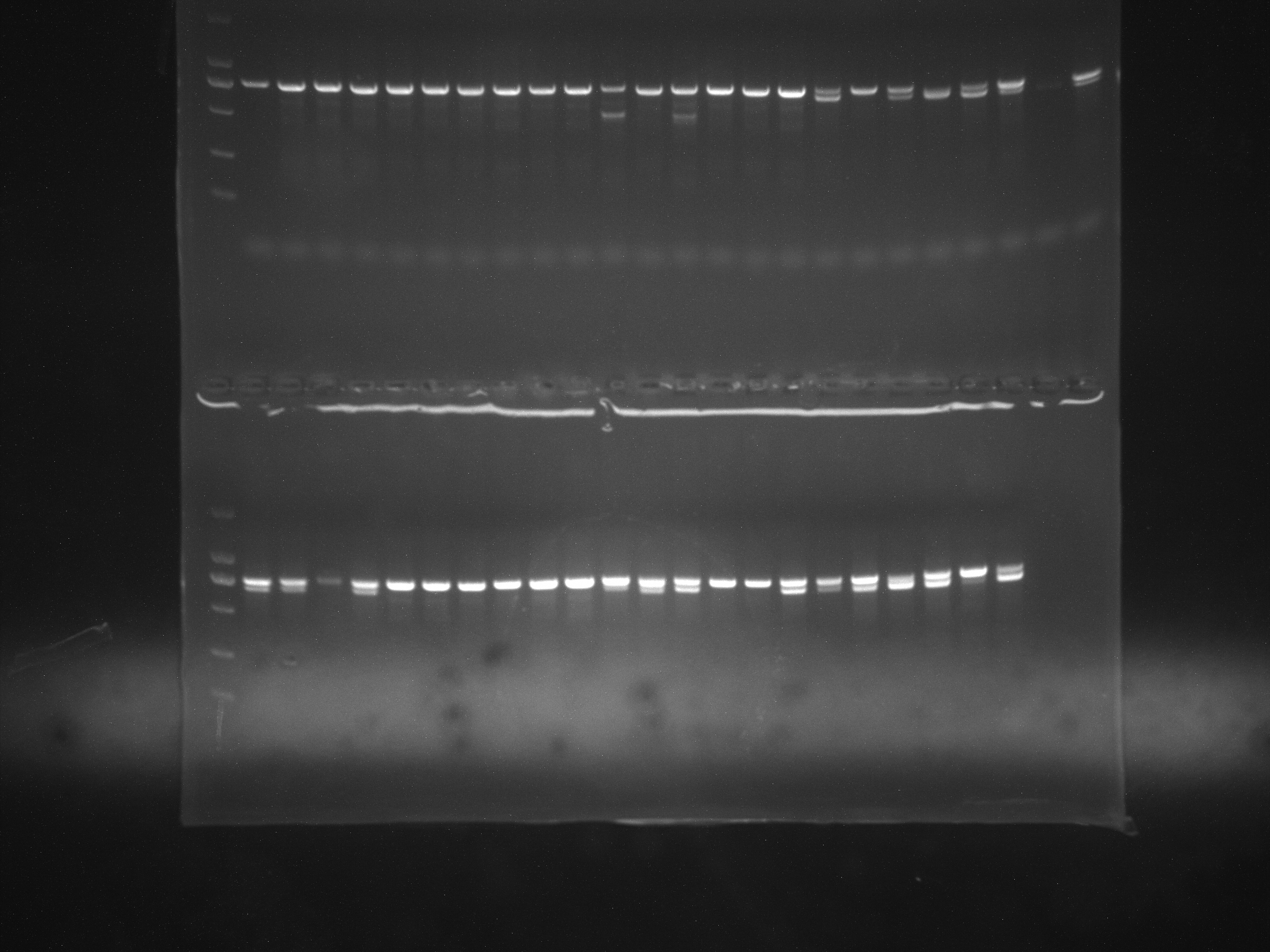

Supplement: Supplementary file 4 [file DataSheet1.ZIP › Supplementary data/figs1/coa ex2 ╥┼┤1⁄2╝°╢¿TU ┤╞F1-1ú¿1-6ú⌐F1-2ú¿1-3ú⌐ F1-3ú¿1-6ú⌐╨█F1-1ú¿1-6ú⌐F1-2ú¿1-6ú⌐ F1-3ú¿1-6ú⌐ F1-4ú¿1-6ú⌐ F1-5ú¿1-6ú⌐20211204-2.jpg]

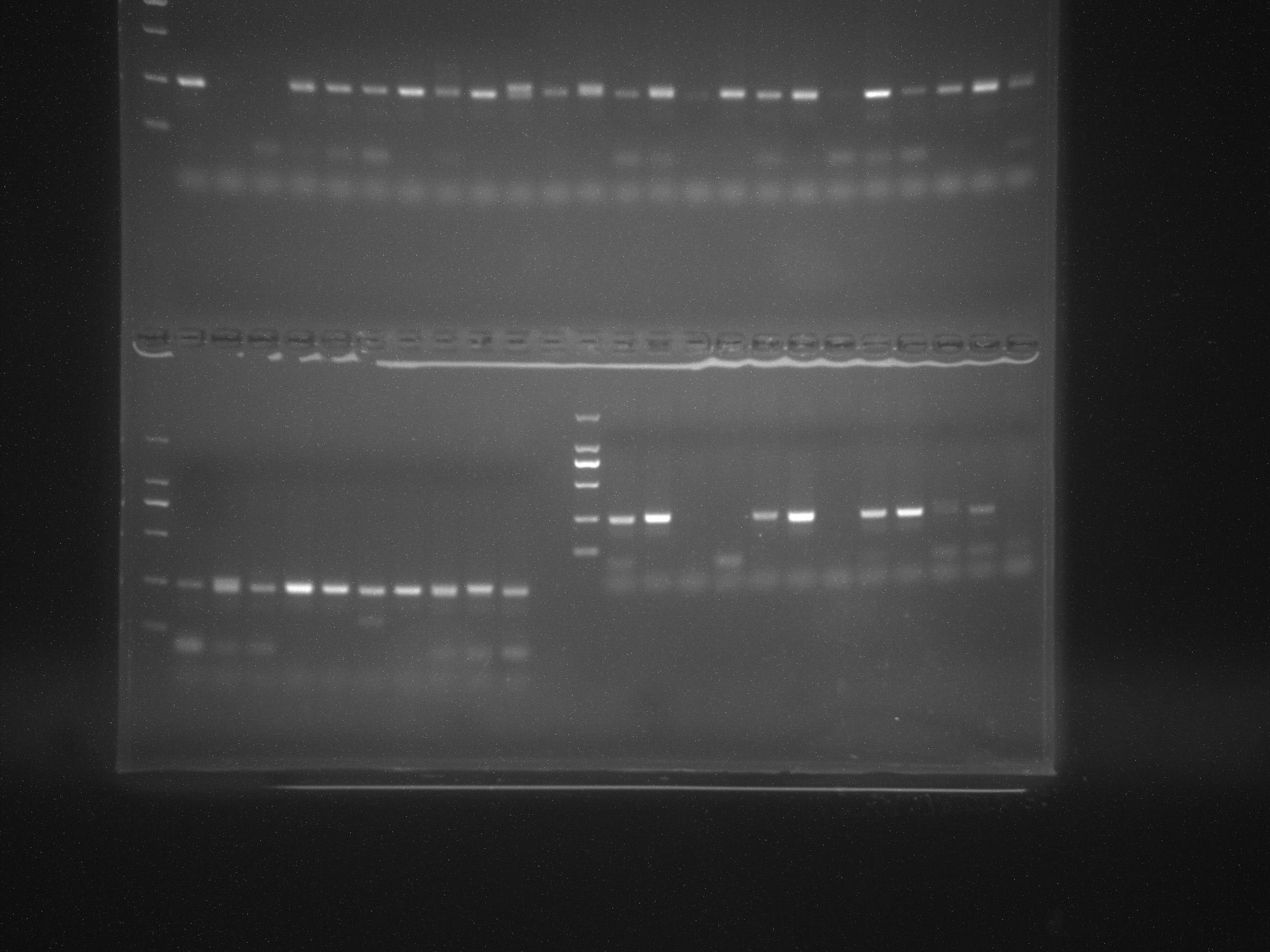

Supplement: Supplementary file 4 [file DataSheet1.ZIP › Supplementary data/figs1/coa ex2F0 ╝⌠╬▓ TU 101-134ú¿╬▐125 5║┼╝⌠ú⌐TU 70-80ú¿4║┼╝⌠ú⌐ 20211106.jpg]

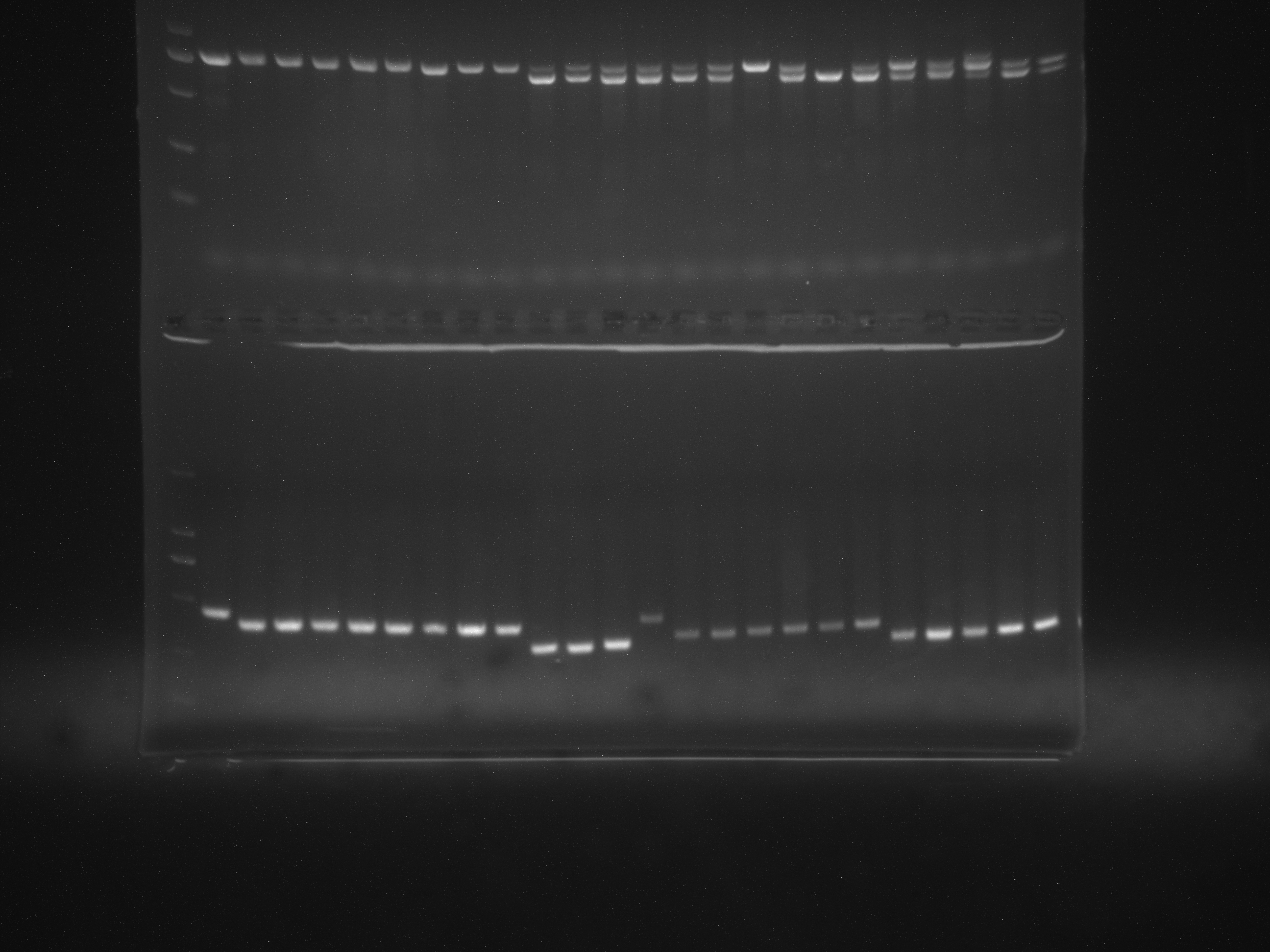

Supplement: Supplementary file 4 [file DataSheet1.ZIP › Supplementary data/figs2/coa ex2 ╥┼┤1⁄2╝°╢¿TU F1-1ú¿1-6ú⌐F1-2ú¿1-2ú⌐ F1-3ú¿1-6ú⌐ F1-4ú¿1-6ú⌐ F1-5ú¿1-3ú⌐ezra┤┐║╧╫╙╝°╢¿ Line2ú¿1-8ú⌐Line3ú¿1-3ú⌐cobll1b┤┐║╧╫╙╝°╢¿ Line2ú¿1-7ú⌐Line3ú¿1-5ú⌐20211129-2.jpg]

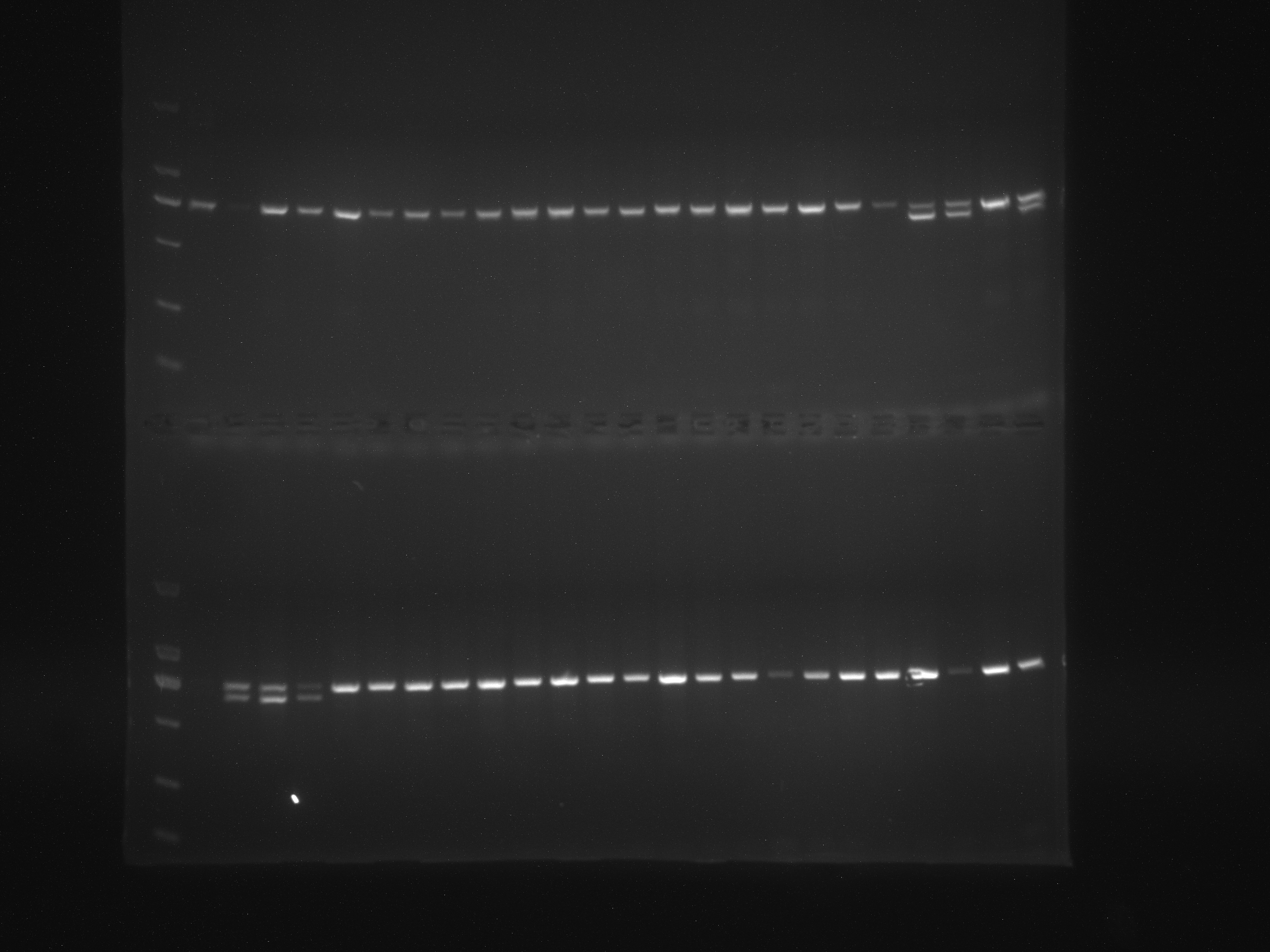

Supplement: Supplementary file 4 [file DataSheet1.ZIP › Supplementary data/figs2/cobll1a F-R2╥┼┤1⁄2╨╘╝°╢¿F1-1 2 3ú¿1-9ú⌐F1-4ú¿1-11ú⌐F1-5ú¿1-9ú⌐20211127.jpg]

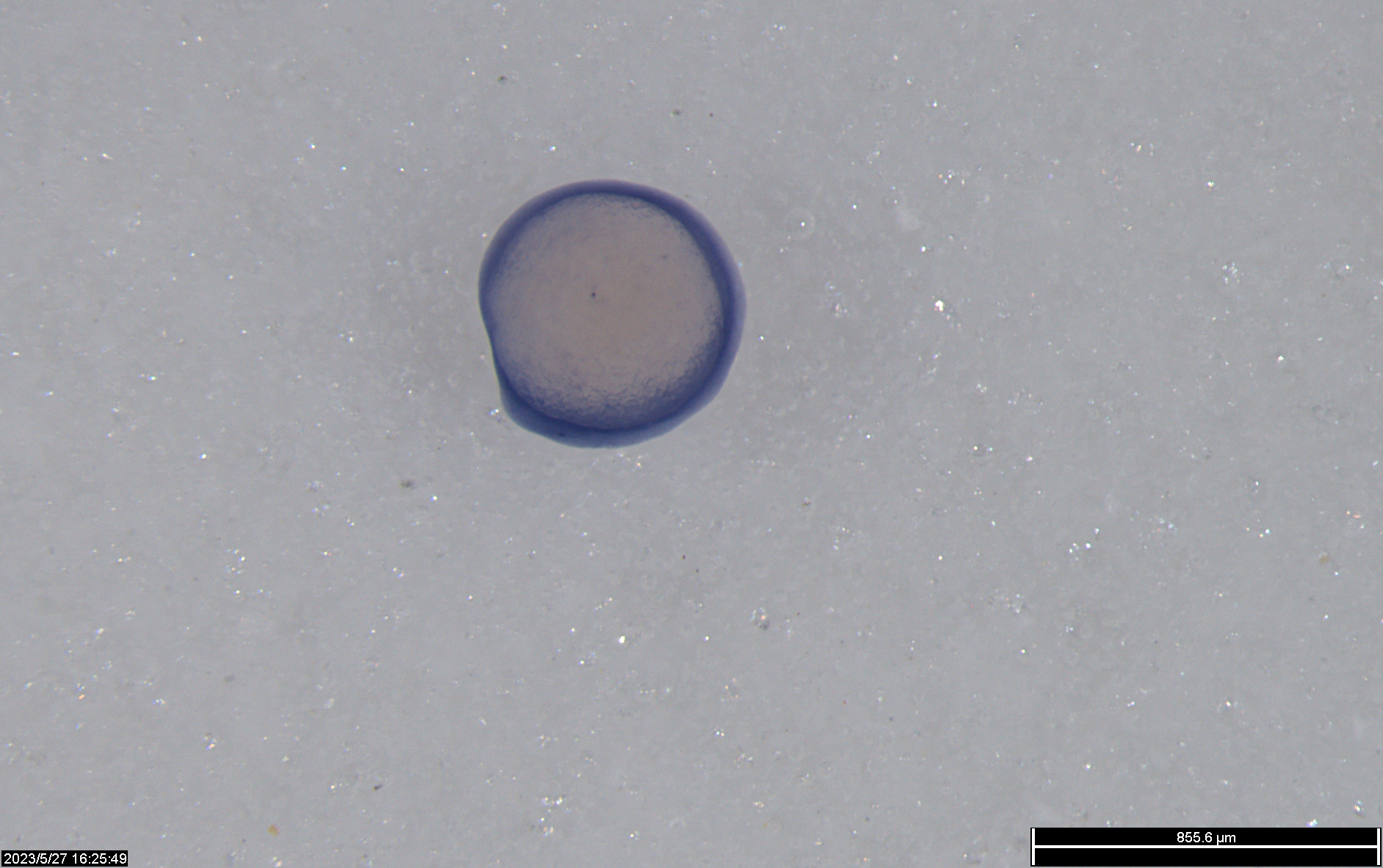

Supplement: Supplementary file 6 [file DataSheet2.ZIP › fig1/╩▒┐╒▒φ┤∩ISH/cobll1a 12h e1_ch00.jpg]

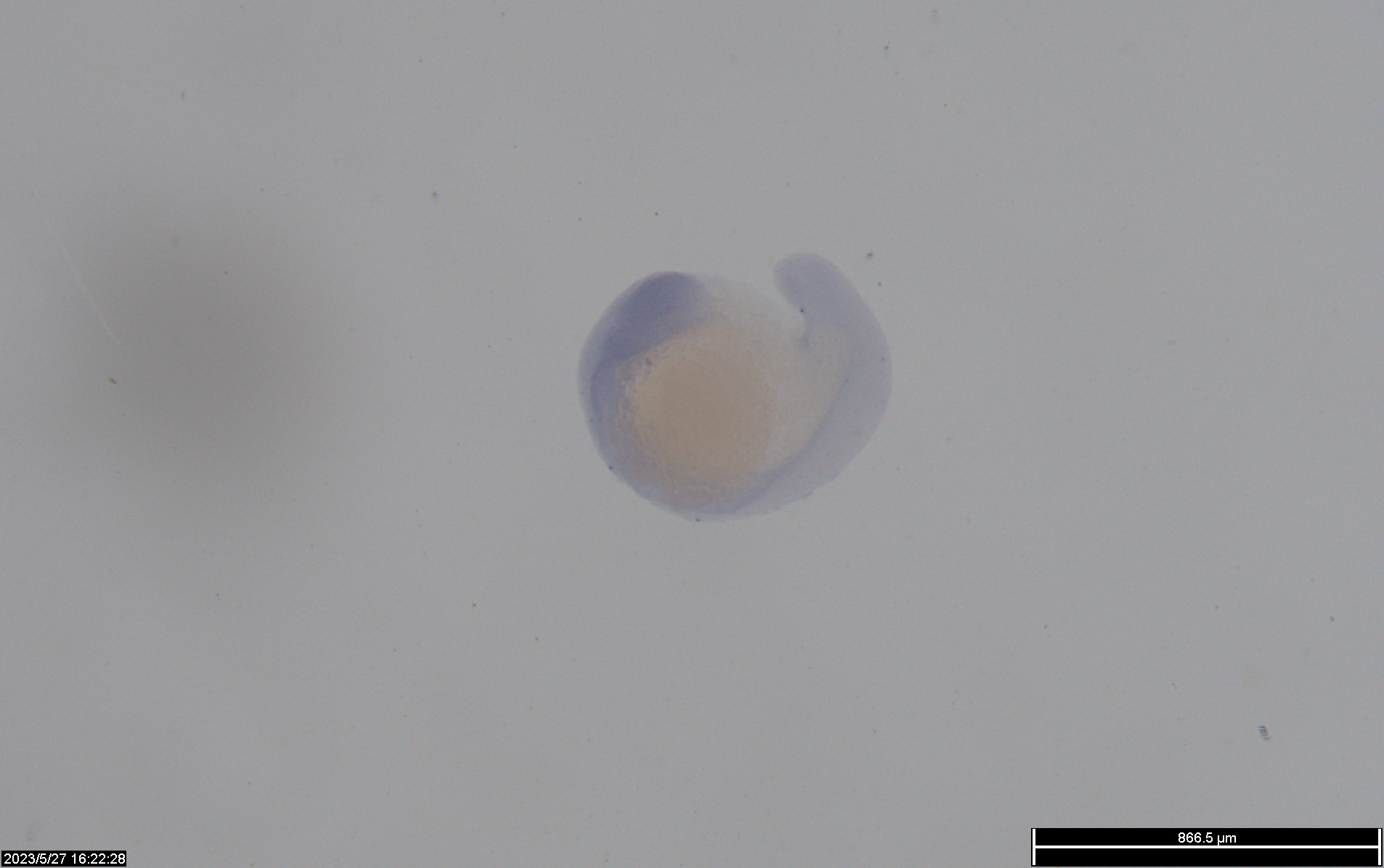

Supplement: Supplementary file 6 [file DataSheet2.ZIP › fig1/╩▒┐╒▒φ┤∩ISH/cobll1a 18ss e1_ch00.jpg]

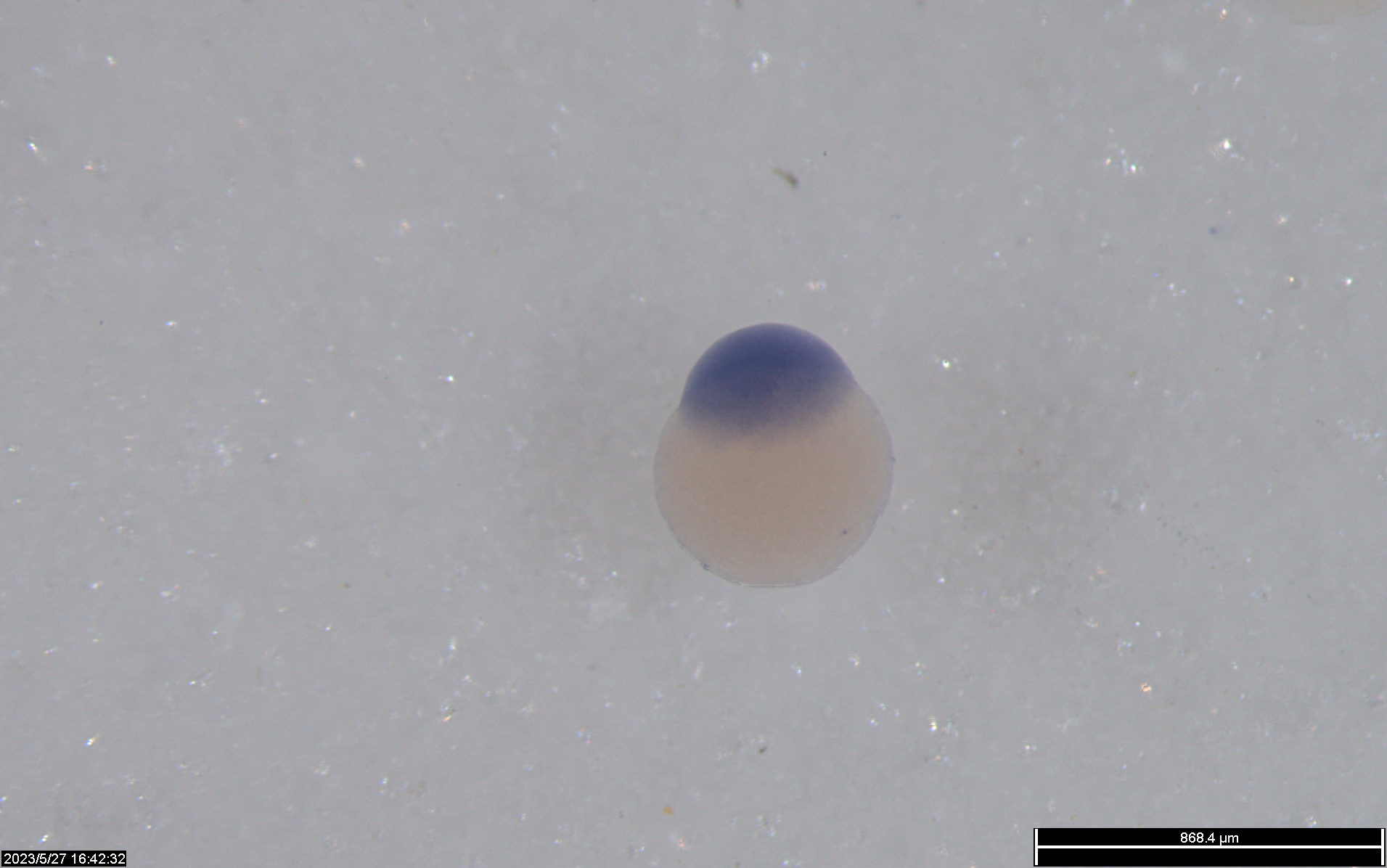

Supplement: Supplementary file 6 [file DataSheet2.ZIP › fig1/╩▒┐╒▒φ┤∩ISH/cobll1a 1cell e2_ch00.jpg]

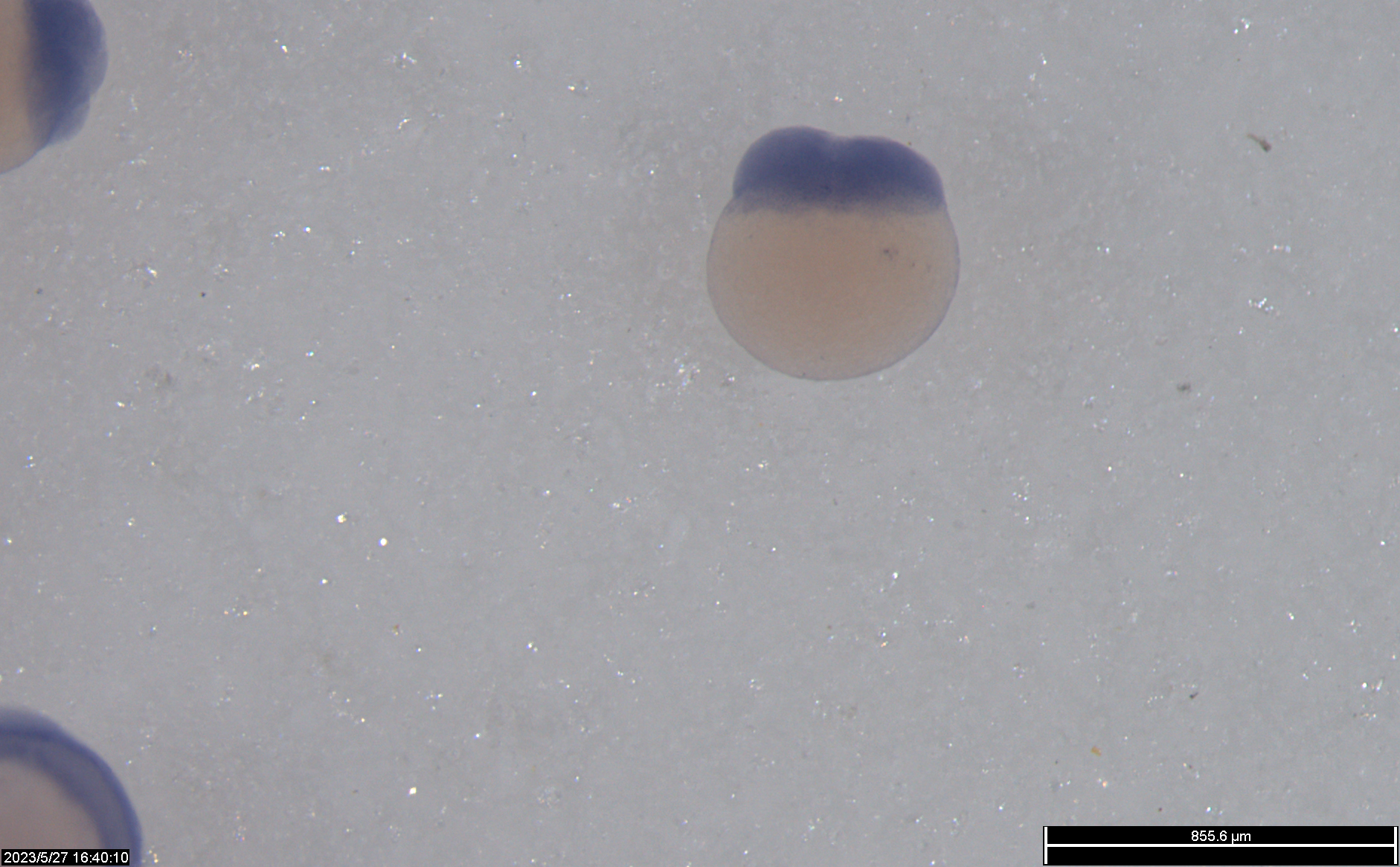

Supplement: Supplementary file 6 [file DataSheet2.ZIP › fig1/╩▒┐╒▒φ┤∩ISH/cobll1a 2cell e1_ch00.jpg]

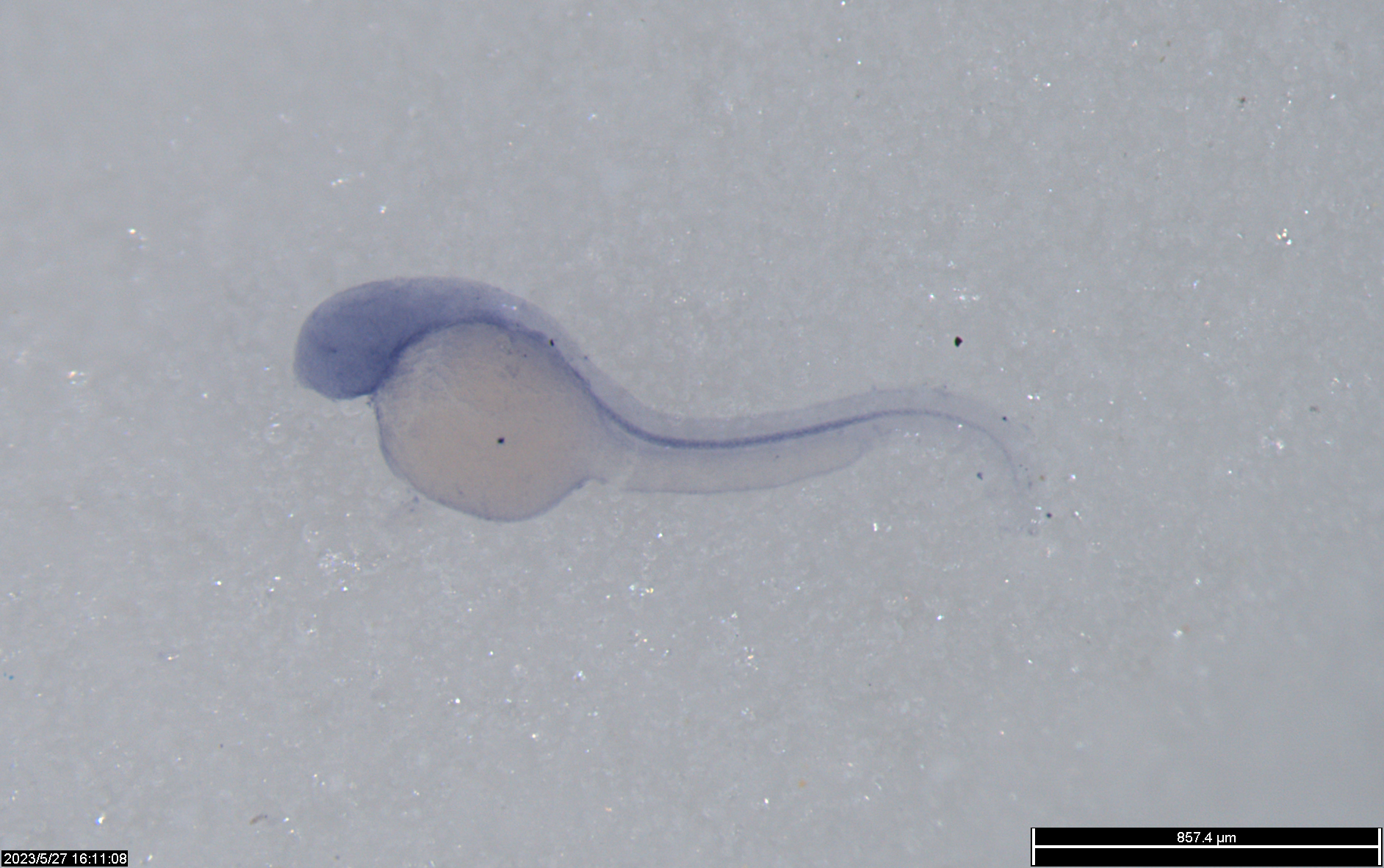

Supplement: Supplementary file 6 [file DataSheet2.ZIP › fig1/╩▒┐╒▒φ┤∩ISH/cobll1a 36h e1_ch00.jpg]

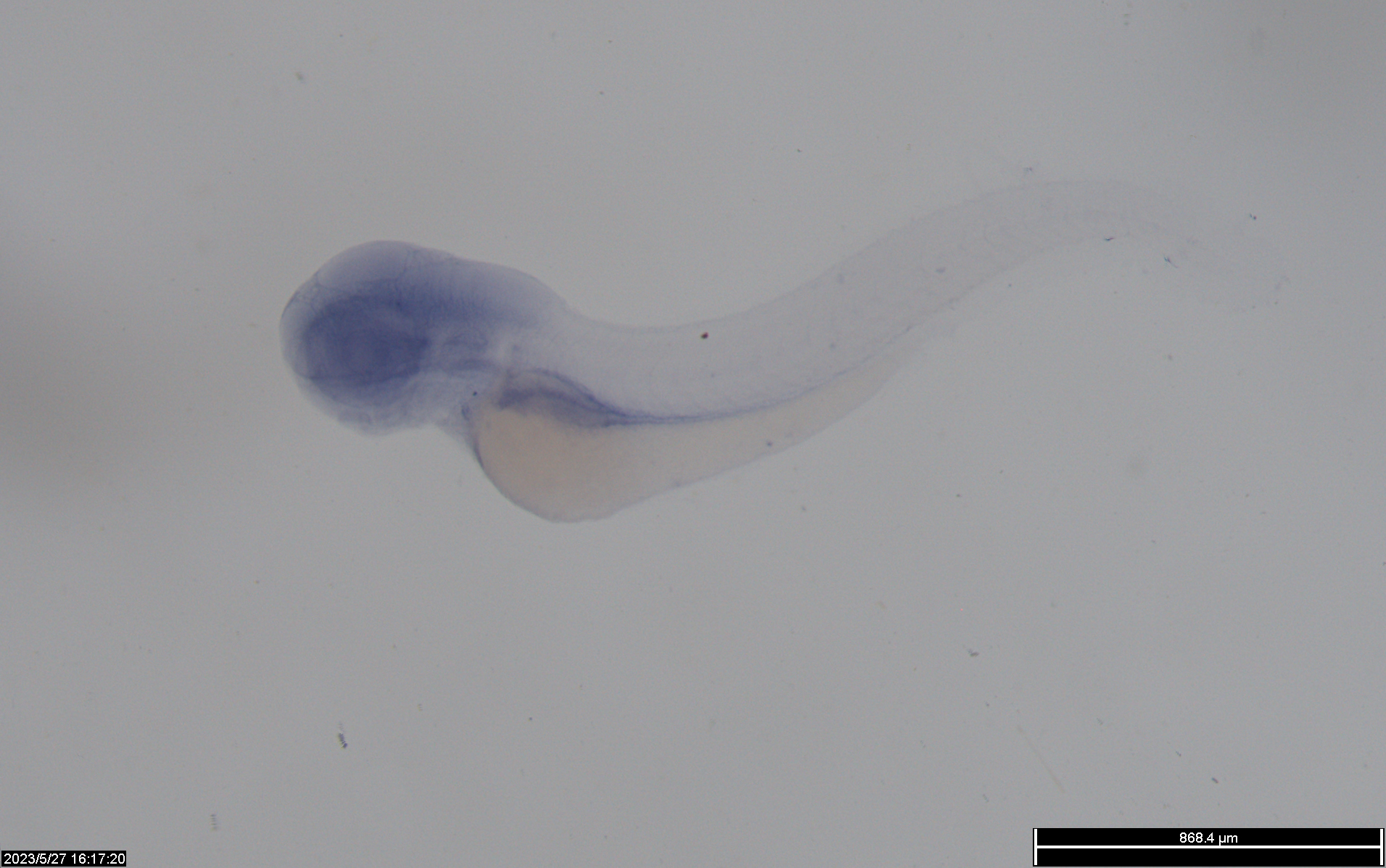

Supplement: Supplementary file 6 [file DataSheet2.ZIP › fig1/╩▒┐╒▒φ┤∩ISH/cobll1a 72h e2_ch00.jpg]

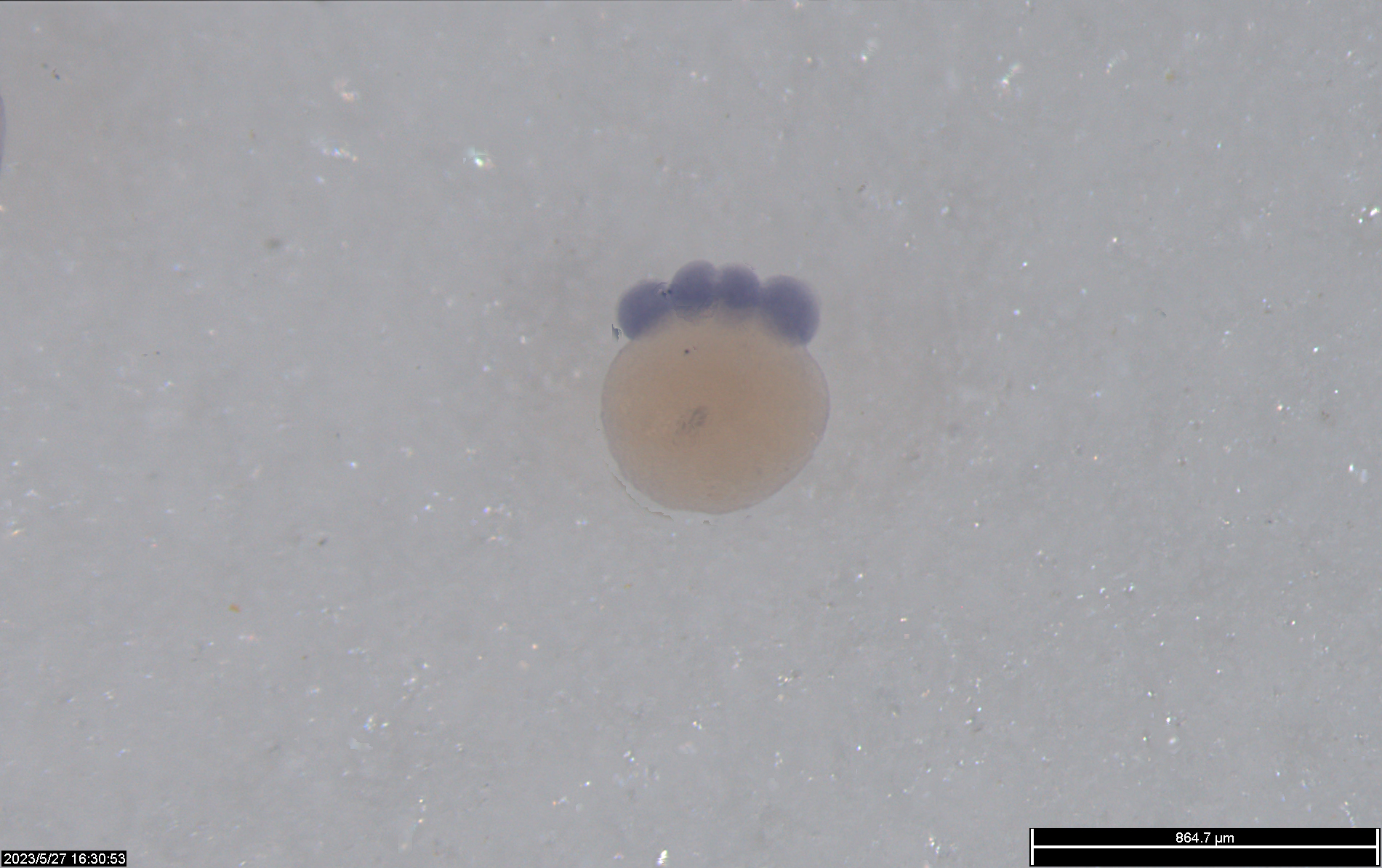

Supplement: Supplementary file 6 [file DataSheet2.ZIP › fig1/╩▒┐╒▒φ┤∩ISH/cobll1a 8cell e1_ch00.jpg]

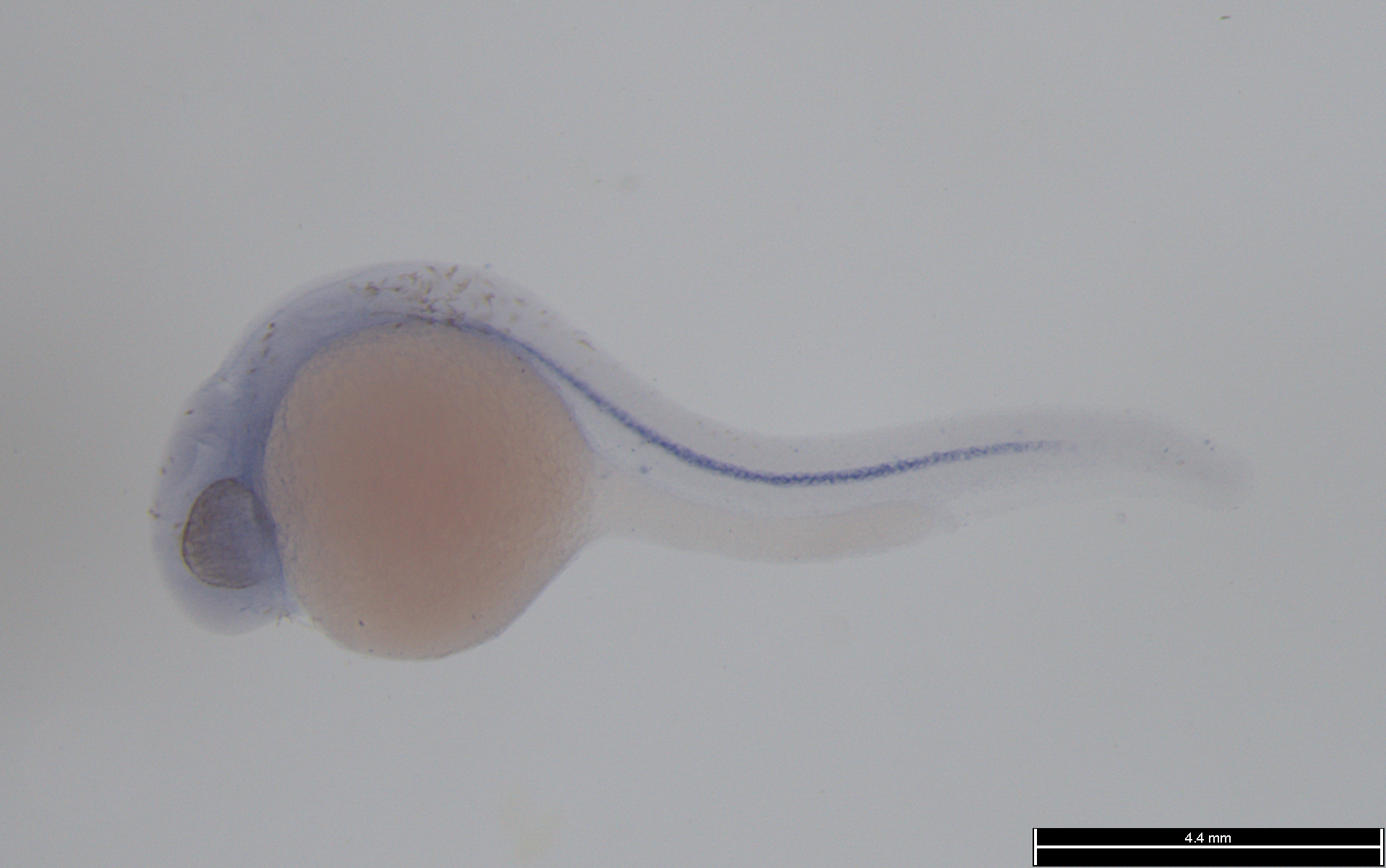

Supplement: Supplementary file 6 [file DataSheet2.ZIP › fig1/╩▒┐╒▒φ┤∩ISH/cobll1a 24h e3_ch00.jpg]

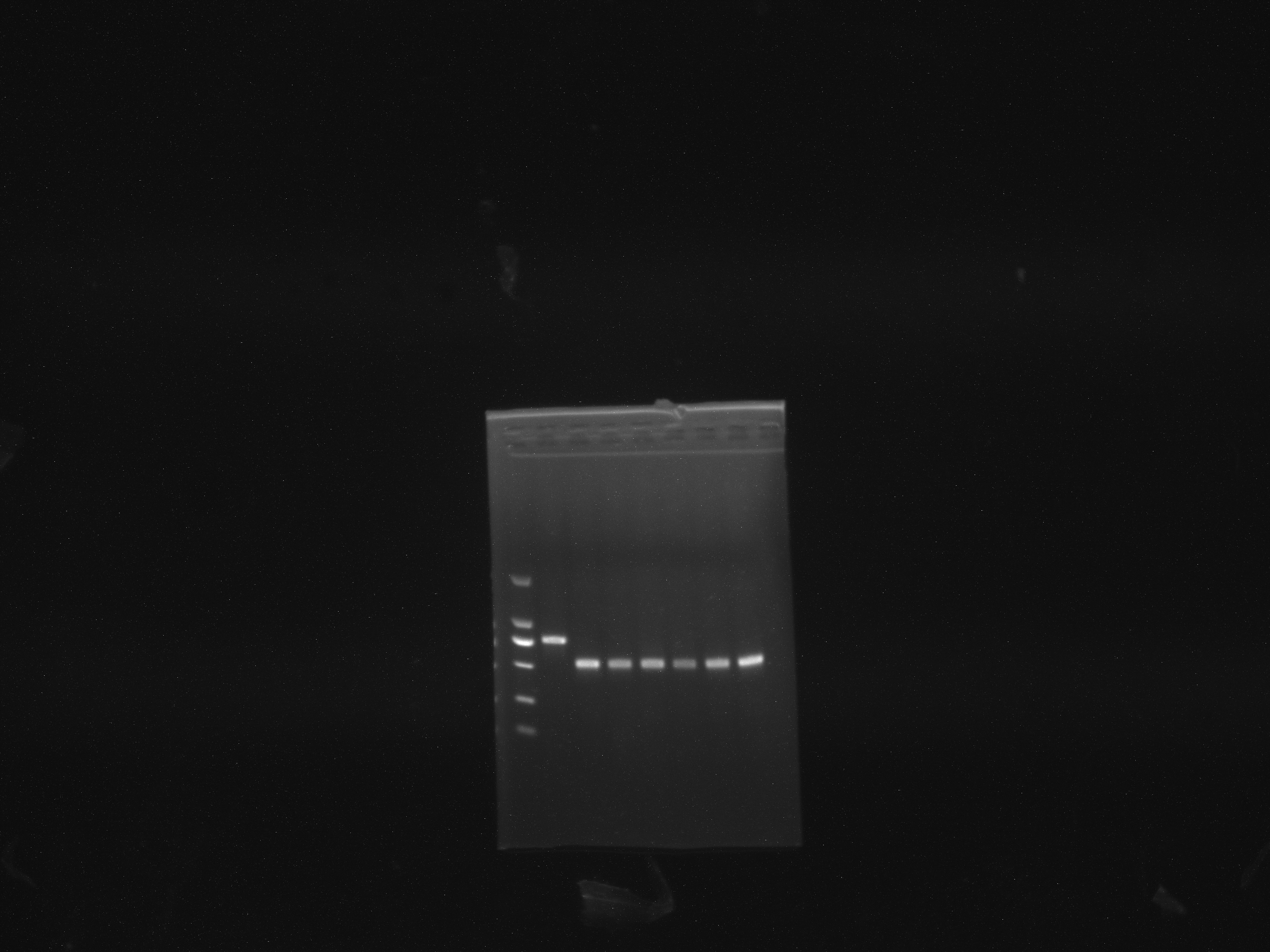

Supplement: Supplementary file 6 [file DataSheet2.ZIP › fig2/cobll1a line2 ┤┐║╧╫╙┼▀╠Ñ╤Θ╓ñtu ┤┐║╧╫╙ 1-5 20230519.jpg]

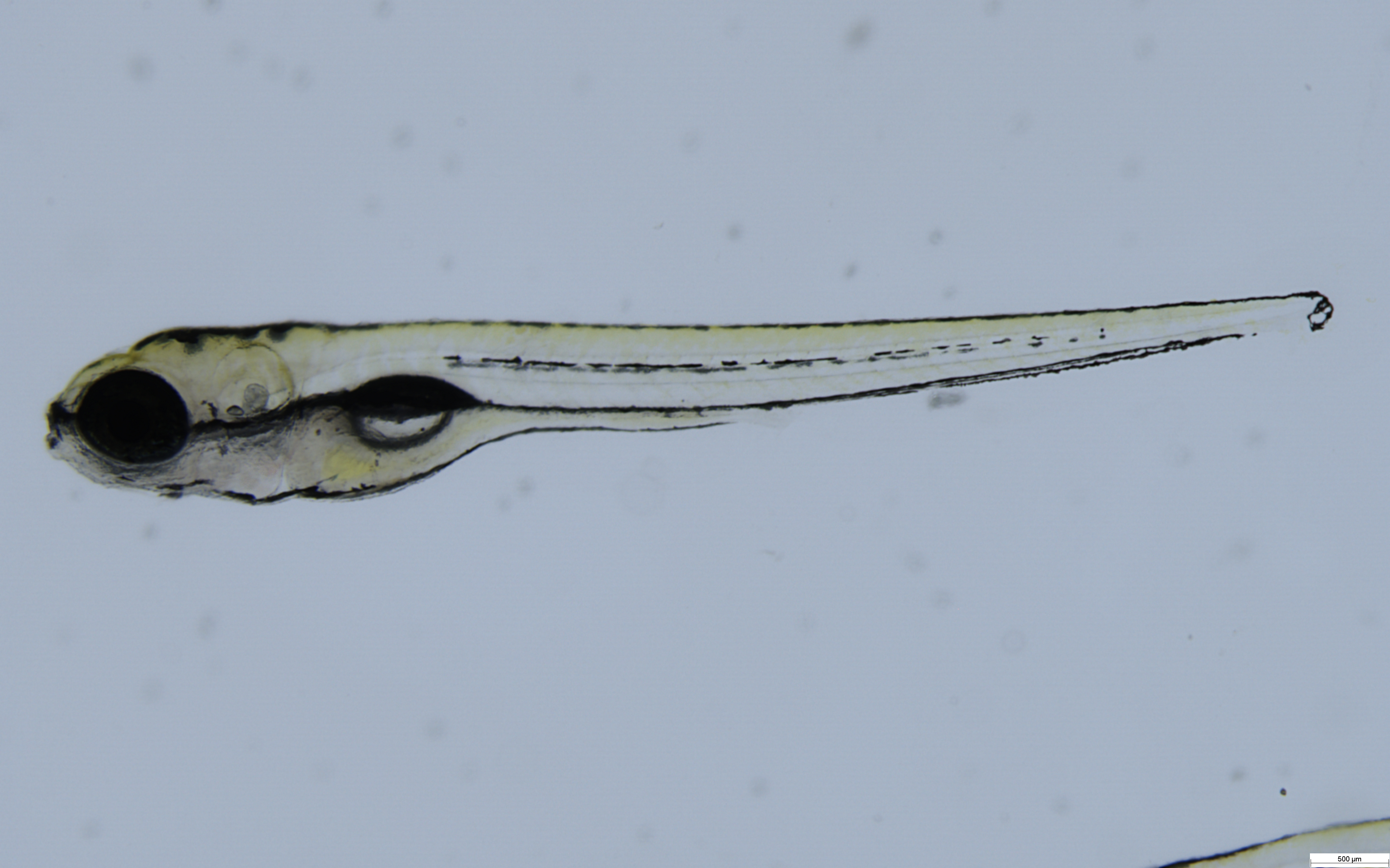

Supplement: Supplementary file 6 [file DataSheet2.ZIP › fig2/cobll1a mutant 5d 2.jpg]

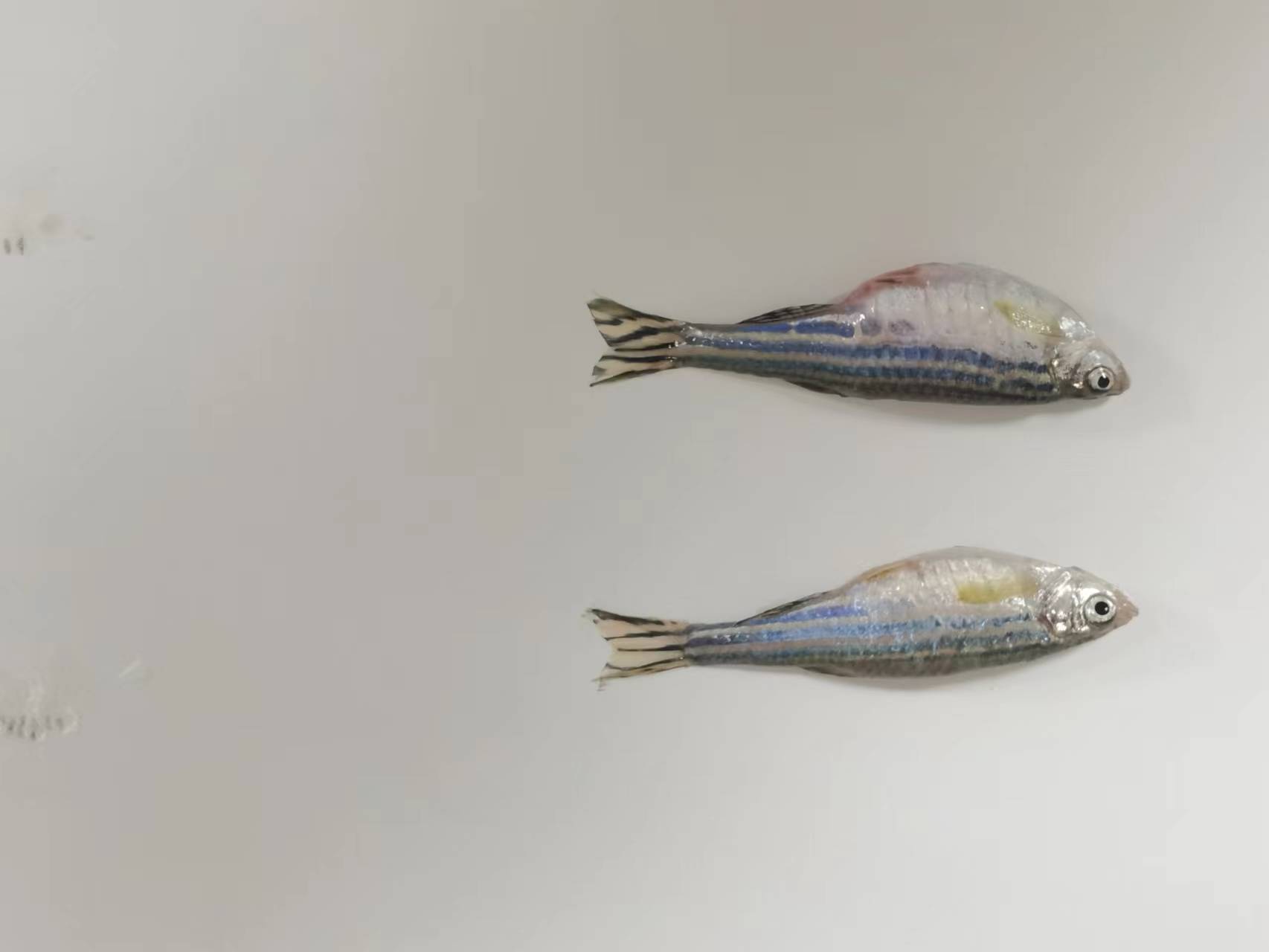

Supplement: Supplementary file 6 [file DataSheet2.ZIP › fig2/cobll1a mutant 8╘┬┴Σ 20230715.jpg]

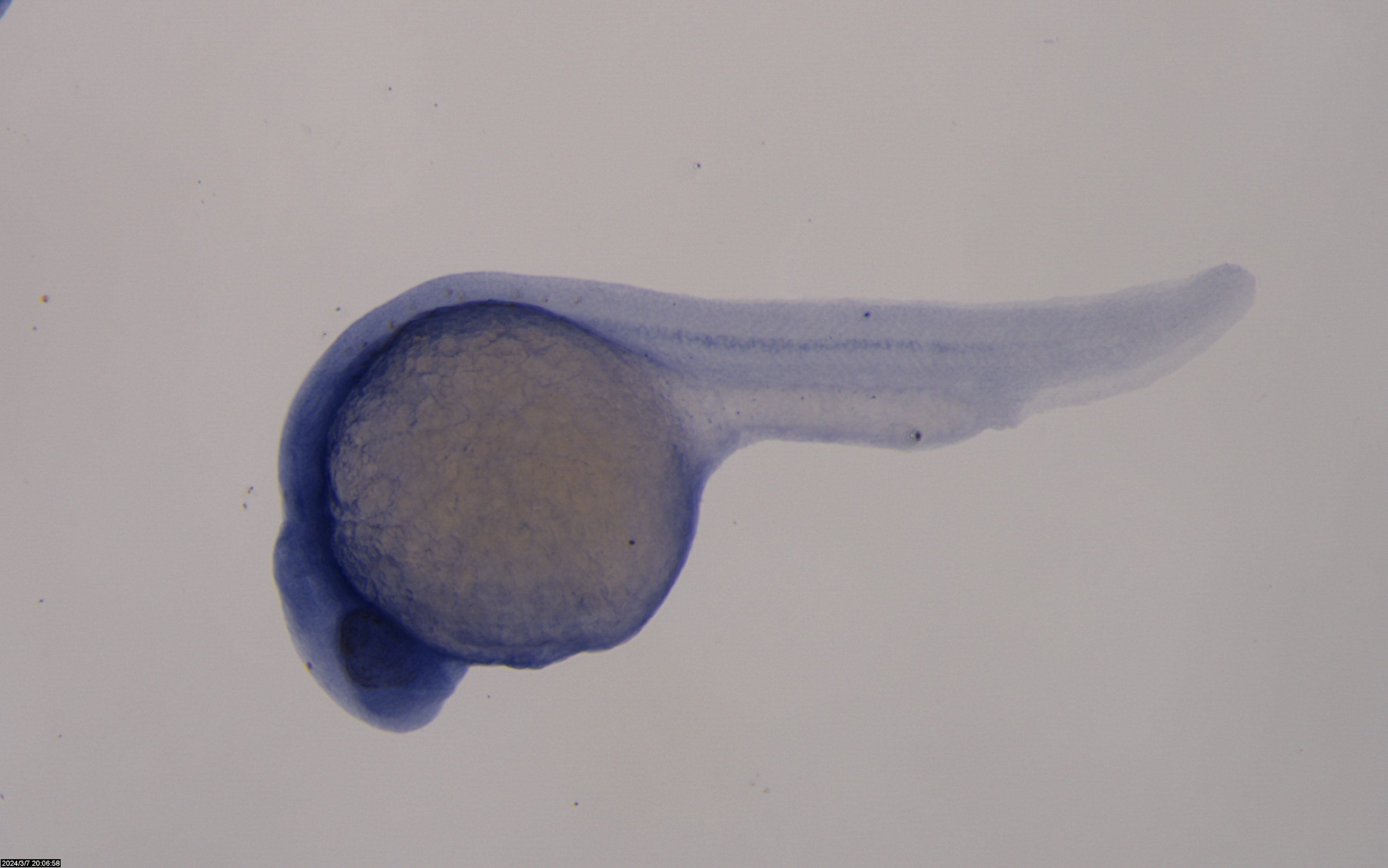

Supplement: Supplementary file 6 [file DataSheet2.ZIP › fig2/cobll1a-coa mut e2.jpg]

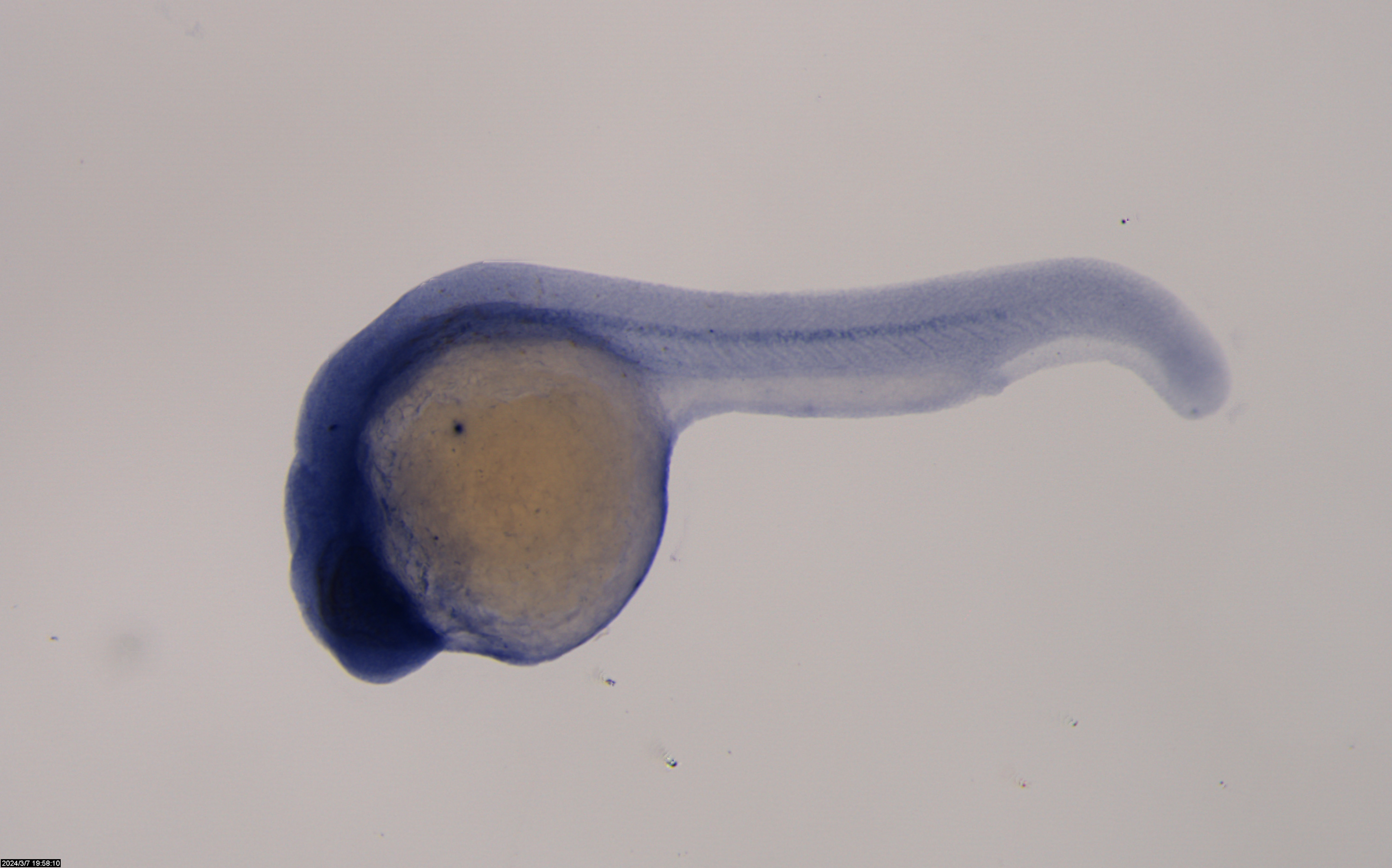

Supplement: Supplementary file 6 [file DataSheet2.ZIP › fig2/cobll1a-WT e3.jpg]

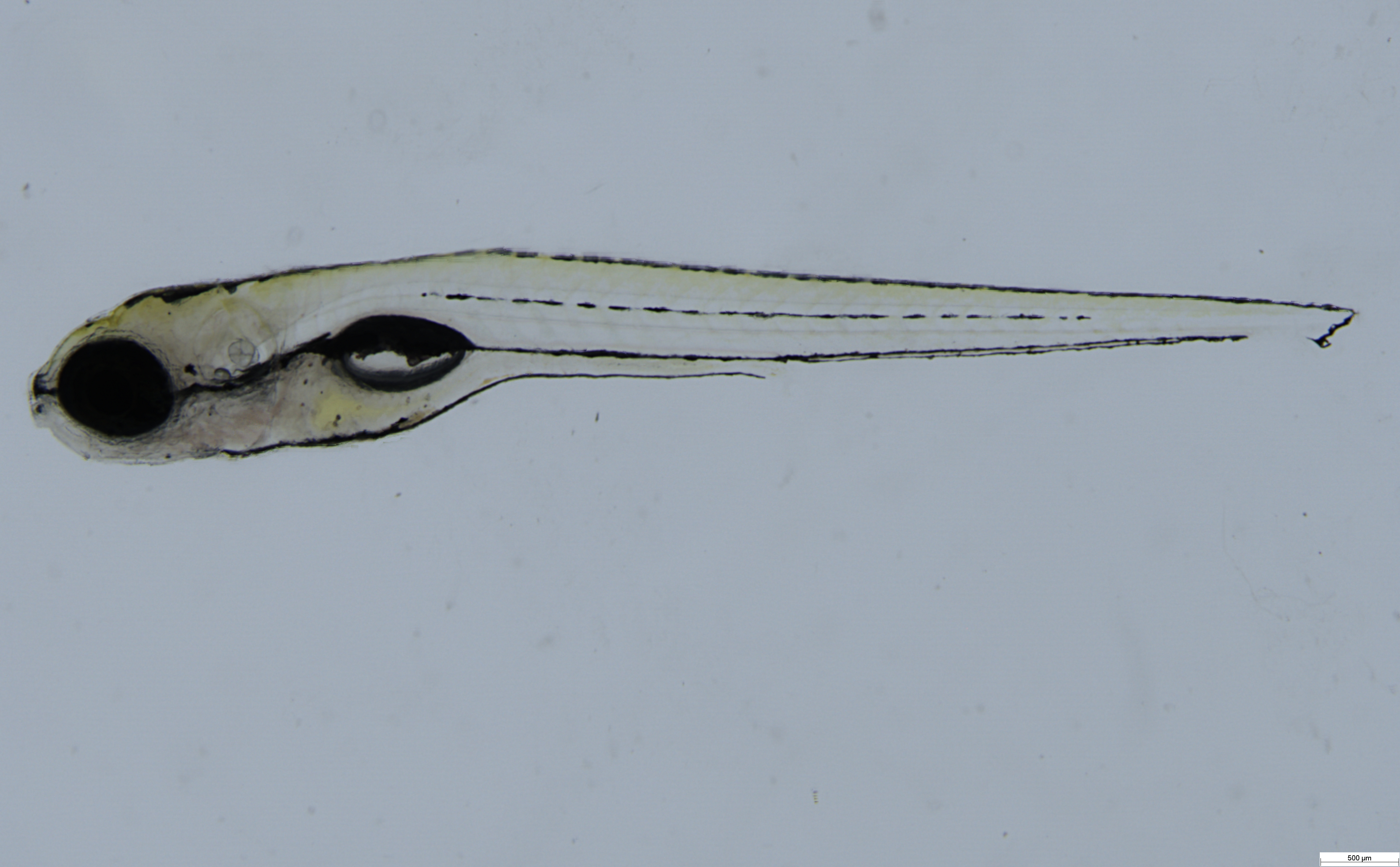

Supplement: Supplementary file 6 [file DataSheet2.ZIP › fig2/control 5d 2.jpg]
