# Supplementary material for: Zebrafish cobll1a regulates lipid homeostasis via the RA signaling pathway
Source: Front Cell Dev Biol. 2024 Apr 18;12:1381362. doi: 10.3389/fcell.2024.1381362 (PMC11063382; doi:10.3389/fcell.2024.1381362)
Supplement: Supplementary file 2 [file Table1.pdf]

Supplementary Material

Zebrafish *cobll1a* regulates lipid homeostasis via the RA signaling pathway

Huaping Xie*, Ting Zeng, Jinrui Lv

*** Correspondence:** hpxie@hunnu.edu.cn

1 Supplementary Figures and Tables

1.1 Supplementary Figures

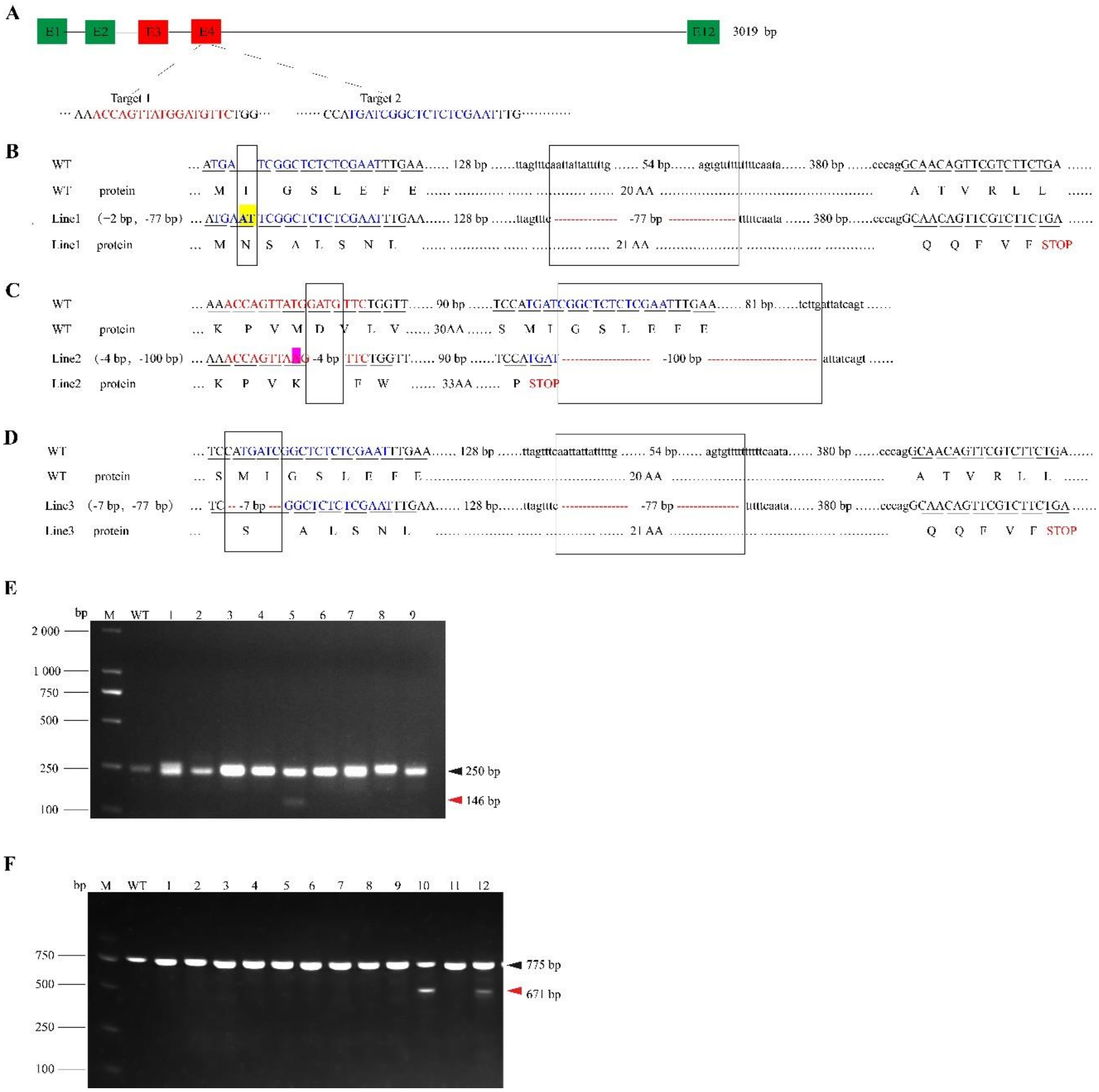

Supplementary Figure 1. Knockout of Zebrafish *cobll1a*. (A) Schematic of the CRSIPR/Cas9 target sites in the *cobll1a* gene. The green box indicates the 3’ UTR and 5’ UTR; the red box represents the exon. The target site 1 sequence is in red, and the target site 2 sequence is in blue. (B-D) Genomic DNA sequence and amino acid sequence alterations in the three independent mutant alleles. Compared to the control, *cobll1a* line1 mutation resulted in a 2 bp insertion and a 77 bp deletion, line 2 mutation led to a 104 bp deletion, and line 3 mutation caused an 84 bp deletion. The target site 1 sequence is shown in red font and the target site 2 sequence is shown in blue font. The rectangular boxes denote insertion or deletion sites in the mutants. Deletions are indicated by red dashed lines. Inserted DNA is highlighted in yellow, and mutated DNA is highlighted in purple. STOP signifies the termination of protein translation. (E) Screening results of *cobll1a* line2 F0 generation adult fish. (F) Screening results of *cobll1a* line2 F1 generation adult fish. M stands for DNA marker; WT refers to the wild type; the black arrowhead indicates the WT band; the red arrowhead refers to the mutant band.

.

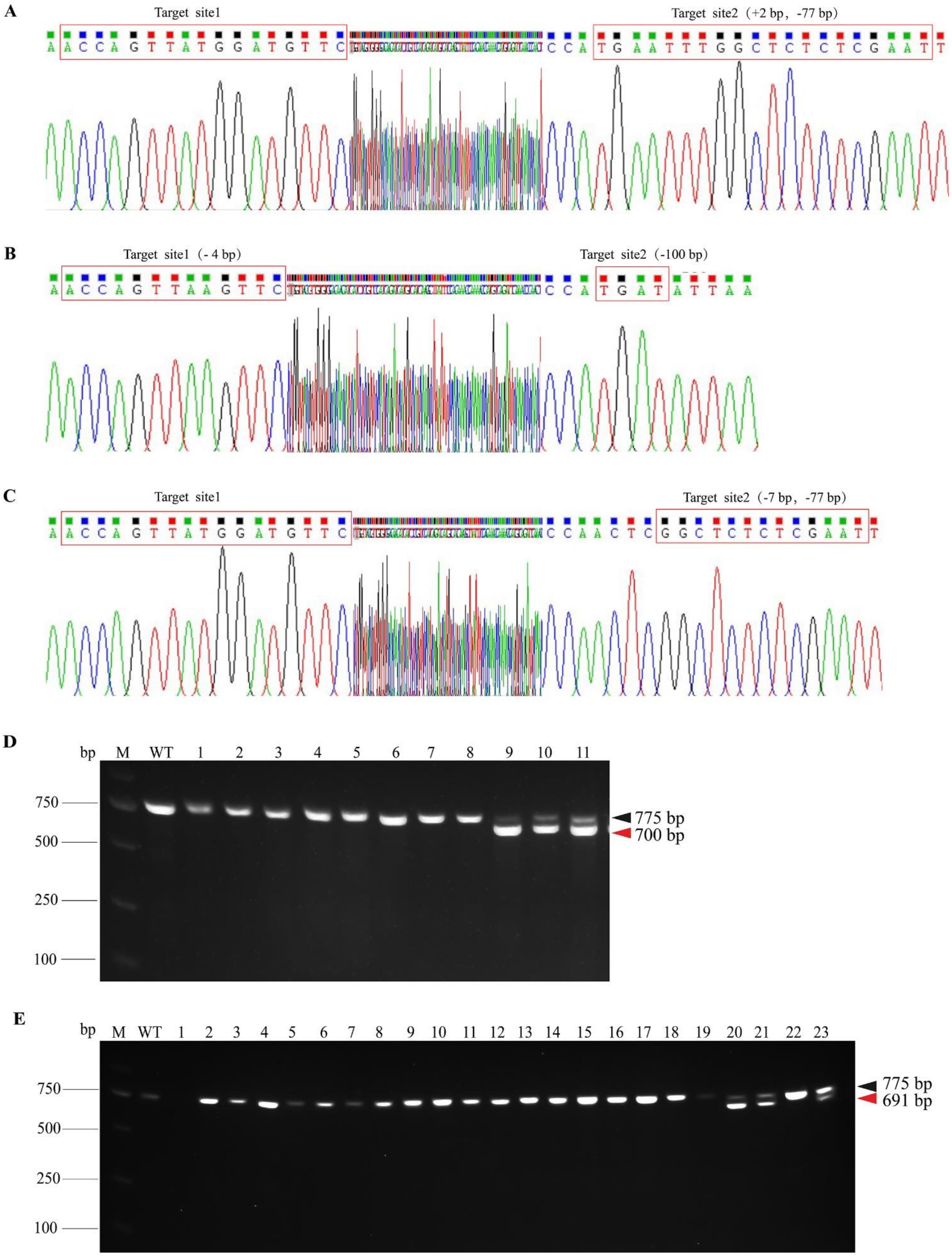
A
Target site1
Target site2（+2 bp，-77 bp）
B
Target site1（- 4 bp）
Target site2（-100 bp）
C
Target site1
Target site2（-7 bp，-77 bp）
D
bp M WT 1 2 3 4 5 6 7 8 9 10 11
750
500
250
100
775 bp
700 bp
E
bp M WT 1 2 3 4 5 6 7 8 9 10 11 12 13 14 15 16 17 18 19 20 21 22 23
750
500
250
100
775 bp
691 bp

Supplementary Figure 2. Sanger sequencing results are presented for the control. (A-C) Sanger sequencing of *cobll1a* Line1 (A), Line2 (B), and Line3 (C) mutants. (D, E) Screening for Line1 (D) and Line3 (E) F1 generation adult fish. M signifies DNA marker; WT stands for wild type; The black and red arrowheads represent the control and knockout alleles, respectively.

1.2 Supplementary Tables

Table S1. Primer sequences used for WISH

Primer Names	Primer Sequences (5′→3′)
cobll1a-ISH-F	CCCGAACACCCAGAAATCCA
cobll1a-ISH-R	TAATACGACTCACTATAGGGAGGGCTGGGATCAAACACTG
fabp10a-ISH-F	GTGGCAGGTTTACGCTCAGG
fabp10a-ISH-R	TAATACGACTCACTATAGGGCGACTGTCAGCGTCTCCACC
fabp2-ISH-F	TTCAACGGGACCTGGAAAGT
fabp2-ISH-R	TAATACGACTCACTATAGGGGGGCTGCCAATCATTAAAGC
insulin-ISH-F	CCATATCCACCATTCCTCGC
insulin-ISH-R	GCGTAATACGACTCACTATAGGCAAACGGAGAGCATTAAGGC
trypsin-ISH-F	TCTGGCTCTTTTCGCTGTGG
trypsin -ISH-R	TAATACGACTCACTATAGGGACACGCCATGATAACGACCT
aldh1a2 ISH -F	ACCGGTACCCAATCCTGAGA
aldh1a2 ISH -R	GCGTAATACGACTCACTATAGGGGCAGAGATGGTCATGGCCTT
rdh10 ISH -F	CACCATGAATATCGCCACCG
rdh10 ISH -R	GCGTAATACGACTCACTATAGGGTGATTCGTTGTTGTTCGTCGAC
cyp26a1 ISH -F	ACCATCGTGCTACCCGTTTT

cyp26a1 ISH -R	GCGTAATACGACTCACTATAGGGGCCTGTTTTCATTGTCGGGG
rbp4 ISH -F	ATAGCAGTGTGTGTCCTGGC
rbp4 ISH -R	GCGTAATACGACTCACTATAGGGGCAGCCTCACAGAAACCAGT
fasn ISH -F	GTTGTGTGTGGTGTCGAAGC
fasn ISH -R	GCGTAATACGACTCACTATAGGGTGCCCAGGGATCTCATCAGA

Table S2. Primer sequences used for quantitative RT-qPCR

**Primer Names**	**Primer Sequences (5′→3′)**
18s-qF	TCGCTAGTTGGCATCGTTTATG
18s-qF	CGGAGGTTCGAAGACGATCA
cobll1a-qF	GCTCCGCCTCTACAAAATCAG
cobll1a-qR	TGAGGACGGGGAAGAGTTTG
cp-qF	GGAGCACTTGGGAATAATGGGT
cp-qR	GTCGGTCCAGAGTTCTTCGG
hhex-qF	GCGTCCATGTACCCGTTCC
hhex-qR	GTTTGATCGTTGGAGAATCG
fabp10a-qF	GCAAGAAGCTCAAGTGCATCG
fabp10a-qR	TGATCATGGTGGTTCCTCCG
insulin-qF	CTCATCTGGTCGATGCCCTTT
insulin-qR	CAGATTTAGGAGGAAGGAAACCC
trypsin-qF	GGCTCTTTTCGCTGTGGCTTAT

trypsin-qR	ACTTGTAGCAGTGAGCAGCA
fabp2-qF	AACTTTACTCTGGGCGTCAC
fabp2-qR	TTGCGTGTGAAAGTCCCCTT
aldh1a2-qF	ACAGTGCTTACCTTGCTACCC
aldh1a2-qR	CTTATCTGCCCATCCAGCGT
rdh10-qF	AGGGCCAAGTGTGCGTTATT
rdh10-qR	CTCTATCTGAAGACCCGGTGG
cyp26a1-qF	AGTGGCCAGCATCAGTGAGAA
cyp26a1-qR	GAACGCCCTCATAATGGCCT
rbp4-qF	TGGAACCATGACAGCCACC
rbp4-qR	CAGCAGCTCCCCAGTACTTC
stra6-qF	GTCAATCTGCTCATGCTCATGA
stra6-qR	GACGGTCGGATACTCCTGTG
rara2a-qF	TTCACCACCCTTACCATCGC
rara2a-qR	CGTTGTGCATCTGTGTTCGG
rara2b-qF	TTGTGGGACTGACCCCTAGC
rara2b-qR	CTGGGTCTCCACAGAGTGAT
rarga-qF	TGGACCCTTTTGCTTGGACC
rarga-qR	GCACCATCTCCTCCGAACTG
rargb-qF	TCGTGTGCCAGGACAAATCT

rargb-qR	GCGCGTCACTTTGTTGATTG
fasn-qF	GAGAAAGCTTGCCAAACAGG
fasn-qR	GAGGGTCTTGCAGGAGACAG
scd-qF	CACCACACGTTTCCCTACGA
scd-qR	CATGGTGTCCACGAAGATGG
elovl2-qF	CACTGGACGAAGTTGGTGAA
elovl2-qR	GTTGAGGACACACCACCAGA
elovl6-qF	CTATGCTCTTCGGGCAGCC
elovl6-qR	ACCATCTGGGTGATCTGTGTCA
dgat1a-qF	CTGGGCGTTTCTGGGTATGA
dgat1a-qR	GCGTTGCCATAGTTACCCCTC
srebf1-qF	CATCCACATGGCTCTGAGTG
srebf1-qR	CTCATCCACAAAGAAGCGGT
srebf2-qF	CACTCACACAAGACACACAG
srebf2-qR	ACCTGGTTCTGGATGAATCG
apoa1a-qF	ACTATGAGCAGTACAAACTCCAGC
apoa1a-qR	CGTAGGGGGTCAAAGCCTG
apoa1b-qF	CCAGCCACATCCACAGATCC
apoa1b-qR	TCGAGGTGAGTGAGGGACTT
apoa4a-qF	GTGGACCAGCATCTGCAGGAGTT
apoa4a-qR	CTTCAGCATAGGGTGTGAGACTC

apoa4b-qF	TACCAGTGGGACTTAAAGCAAACA
apoa4b-qR	TGTGAATGCCACAAAGGCAAG
apoa2-qF	TGGTGGTTTGGCCTCTGAAC
apoa2-qR	GGTACGCACCAACATAGGGT
apoea-qF	TCGGCAGAGAATTAGACACAC
apoea-qR	TCGTTGAACTTCTGGGCTCTC
apoeb-qF	TGACGTGAAGAACCGTGTCG
apoeb-qR	TAGGTTGCTACGGTGTTGCG
apoba-qF	CTCTTTGGAGAGCGCTTGGA
apoba-qR	AGCGTGGAACGAAGACCATT
apobb.1-qF	GTCGTTATGCTTTTGCACCA
apobb.1-qR	CGATGGCATTGTCAATCAAG
lpl-qF	GGAATACACGGCGAGAAGGAG
lpl-qR	TGTCGGAGTTCCACCAGGC
lipc-qF	CACTGGCAAAAGCAAGAGGC
lipc-qR	TCTCCTCGACAAGTGTTA TGGG
