# Supplementary material for: Zebrafish cobll1a regulates lipid homeostasis via the RA signaling pathway
Source: Front Cell Dev Biol. 2024 Apr 18;12:1381362. doi: 10.3389/fcell.2024.1381362 (PMC11063382; doi:10.3389/fcell.2024.1381362)
Supplement: Supplementary file 5 [file DataSheet5.docx]

Supplementary Material

Zebrafish *cobll1a* regulate early hepatic development by con-trolling lipid transportation via the RA signaling pathway

Huaping Xie*, Ting Zeng, Jinrui Lv

*** Correspondence:** hpxie@hunnu.edu.cn

# Supplementary Figures and Tables

## Supplementary Figures


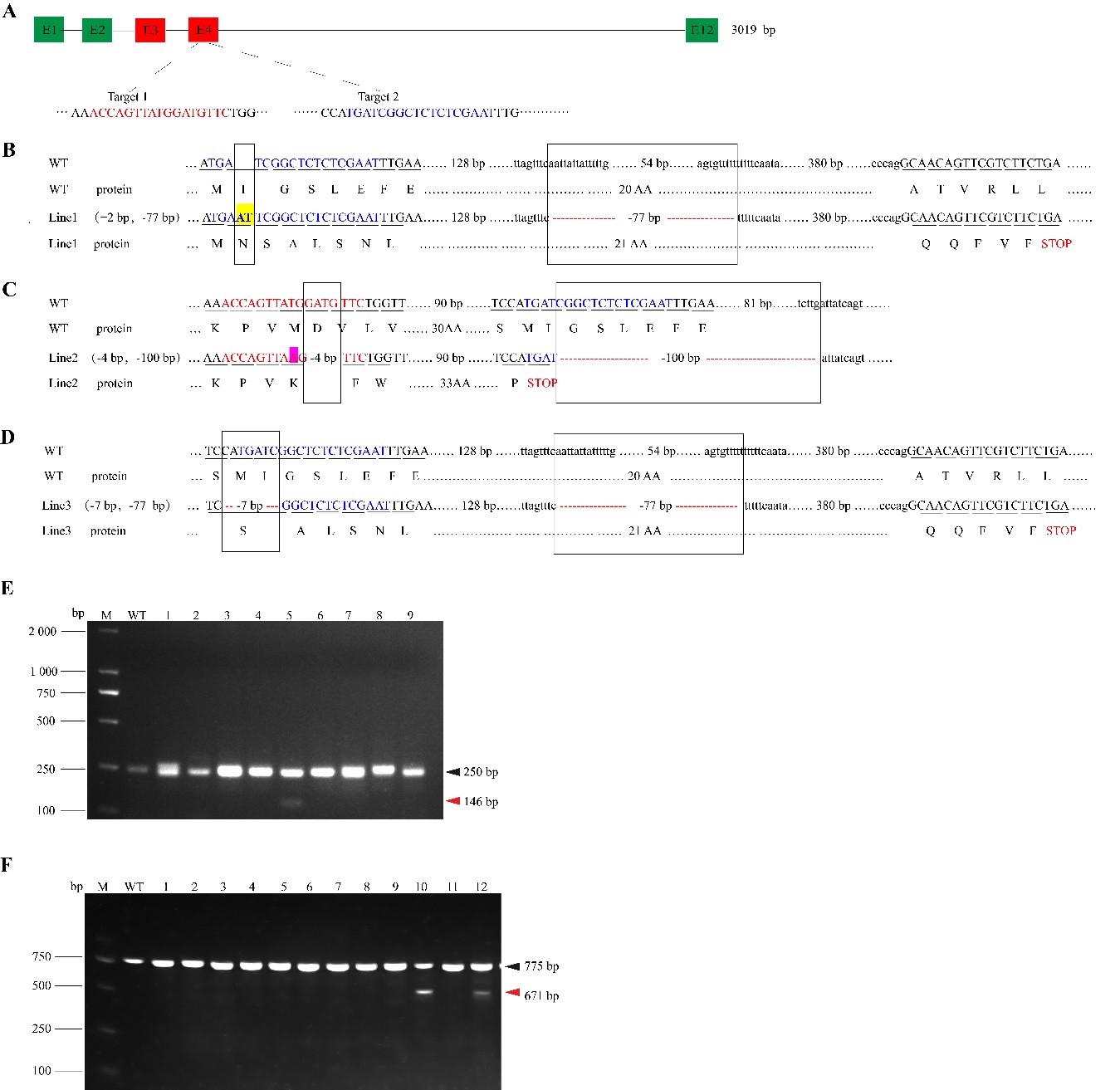


**Supplementary Figure 1. Knockout of Zebrafish *cobll1a*. (**A) Schematic of the CRSIPR/Cas9 target sites in the *cobll1a* gene. The green box indicates the 3’ UTR and 5’ UTR; the red box represents the exon. The target site 1 sequence is in red, and the target site 2 sequence is in blue. (B-D) Genomic DNA sequence and amino acid sequence alterations in the three independent mutant alleles. Compared to the control, *cobll1a* line1 mutation resulted in a 2 bp insertion and a 77 bp deletion, line 2 mutation led to a 104 bp deletion, and line 3 mutation caused an 84 bp deletion. The target site 1 sequence is shown in red font and the target site 2 sequence is shown in blue font. The rectangular boxes denote insertion or deletion sites in the mutants. Deletions are indicated by red dashed lines. Inserted DNA is highlighted in yellow, and mutated DNA is highlighted in purple. STOP signifies the termination of protein translation. (E) Screening results of *cobll1a* line2 F0 generation adult fish. (F) Screening results of *cobll1a* line2 F1 generation adult fish. M stands for DNA marker; WT refers to the wild type; the black arrowhead indicates the WT band; the red arrowhead refers to the mutant band.

.


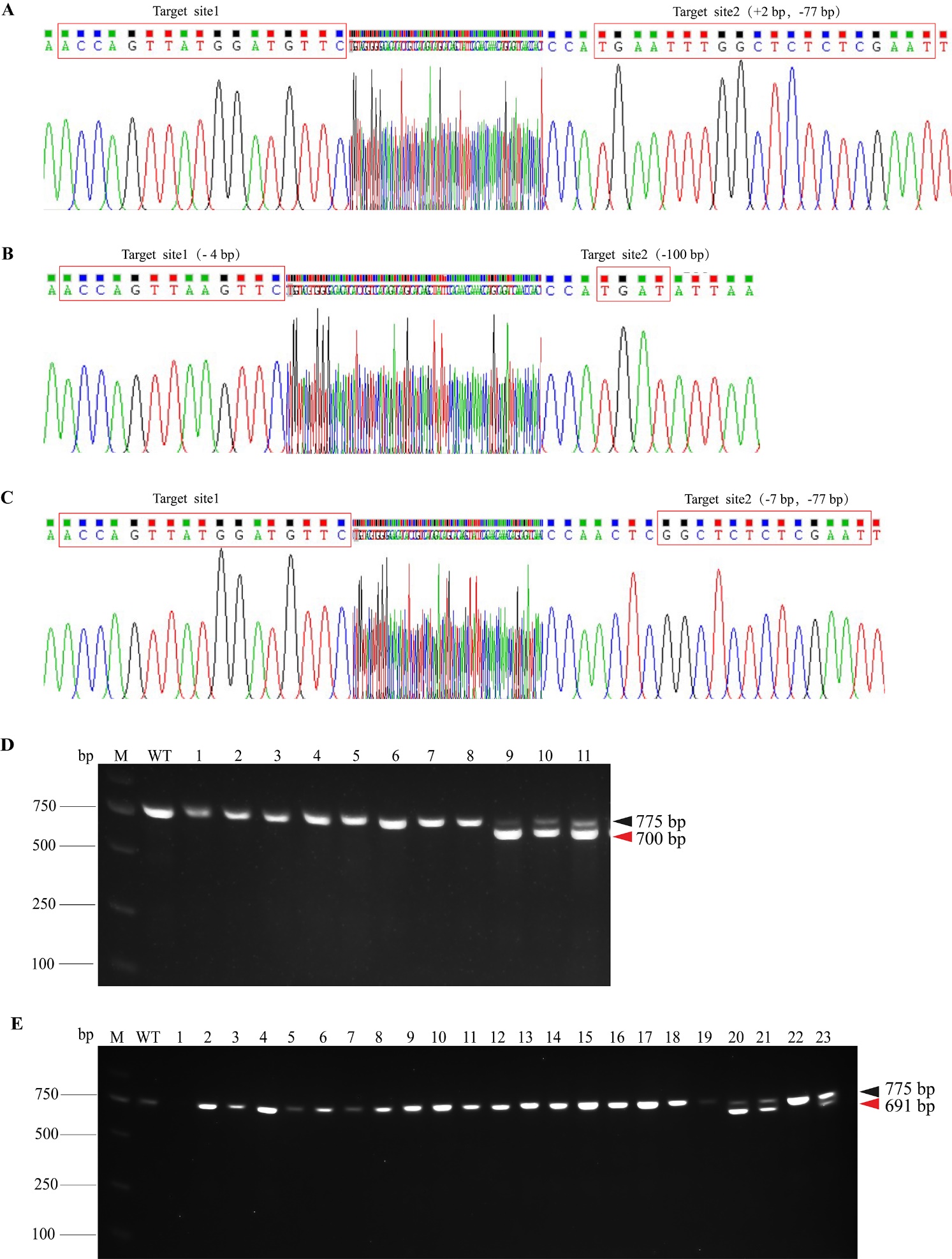


**Supplementary Figure 2. Sanger sequencing results are presented for the control.** (A-C) Sanger sequencing of *cobll1a* Line1 (A), Line2 (B), and Line3 (C) mutants. (D, E) Screening for Line1 (D) and Line3 (E) F1 generation adult fish. M signifies DNA marker; WT stands for wild type; The black and red arrowheads represent the control and knockout alleles, respectively.

## Supplementary Tables

Table S1. Primer sequences used for WISH

| **Primer Names** | **Primer Sequences (5′→3′)** |
| --- | --- |
| *cobll1a*-ISH-F | CCCGAACACCCAGAAATCCA |
| *cobll1a*-ISH-R | TAATACGACTCACTATAGGGAGGGCTGGGATCAAACACTG |
| *fabp10a*-ISH-F | GTGGCAGGTTTACGCTCAGG |
| *fabp10a*-ISH-R | TAATACGACTCACTATAGGGCGACTGTCAGCGTCTCCACC |
| *fabp2*-ISH-F | TTCAACGGGACCTGGAAAGT |
| *fabp2*-ISH-R | TAATACGACTCACTATAGGGGGGCTGCCAATCATTAAAGC |
| *insulin*-ISH-F | CCATATCCACCATTCCTCGC |
| *insulin*-ISH-R | GCGTAATACGACTCACTATAGGCAAACGGAGAGCATTAAGGC |
| *trypsin*-ISH-F | TCTGGCTCTTTTCGCTGTGG |
| *trypsin* -ISH-R | TAATACGACTCACTATAGGGACACGCCATGATAACGACCT |
| *aldh1a2* ISH -F | ACCGGTACCCAATCCTGAGA |
| *aldh1a2* ISH -R | GCGTAATACGACTCACTATAGGGGCAGAGATGGTCATGGCCTT |
| *rdh10* ISH -F | CACCATGAATATCGCCACCG |
| *rdh10* ISH -R | GCGTAATACGACTCACTATAGGGTGATTCGTTGTTGTTCGTCGAC |
| *cyp26a1* ISH -F | ACCATCGTGCTACCCGTTTT |
| *cyp26a1* ISH -R | GCGTAATACGACTCACTATAGGGGCCTGTTTTCATTGTCGGGG |
| *rbp4* ISH -F | ATAGCAGTGTGTGTCCTGGC |
| *rbp4* ISH -R | GCGTAATACGACTCACTATAGGGGCAGCCTCACAGAAACCAGT |
| *fasn* ISH -F | GTTGTGTGTGGTGTCGAAGC |
| *fasn* ISH -R | GCGTAATACGACTCACTATAGGGTGCCCAGGGATCTCATCAGA |

Table S2. Primer sequences used for quantitative RT-qPCR

| **Primer Names** | **Primer Sequences (5′→3′)** |
| --- | --- |
| *18s*-qF | TCGCTAGTTGGCATCGTTTATG |
| *18s*-qF | CGGAGGTTCGAAGACGATCA |
| *cobll1a*-qF | GCTCCGCCTCTACAAAATCAG |
| *cobll1a*-qR | TGAGGACGGGGAAGAGTTTG |
| *cp*-qF | GGAGCACTTGGGAATAATGGGT |
| *cp*-qR | GTCGGTCCAGAGTTCTTCGG |
| *hhex*-qF | GCGTCCATGTACCCGTTCC |
| *hhex*-qR | GTTTGATCGTTGGAGAATCG |
| *fabp10a*-qF | GCAAGAAGCTCAAGTGCATCG |
| *fabp10a*-qR | TGATCATGGTGGTTCCTCCG |
| *insulin*-qF | CTCATCTGGTCGATGCCCTTT |
| *insulin*-qR | CAGATTTAGGAGGAAGGAAACCC |
| *trypsin*-qF | GGCTCTTTTCGCTGTGGCTTAT |
| *trypsin*-qR | ACTTGTAGCAGTGAGCAGCA |
| *fabp2*-qF | AACTTTACTCTGGGCGTCAC |
| *fabp2*-qR | TTGCGTGTGAAAGTCCCCTT |
| *aldh1a2*-qF | ACAGTGCTTACCTTGCTACCC |
| *aldh1a2*-qR | CTTATCTGCCCATCCAGCGT |
| *rdh10*-qF | AGGGCCAAGTGTGCGTTATT |
| *rdh10*-qR | CTCTATCTGAAGACCCGGTGG |
| *cyp26a1*-qF | AGTGGCCAGCATCAGTGAGAA |
| *cyp26a1*-qR | GAACGCCCTCATAATGGCCT |
| *rbp4*-qF | TGGAACCATGACAGCCACC |
| *rbp4*-qR | CAGCAGCTCCCCAGTACTTC |
| *stra6*-qF | GTCAATCTGCTCATGCTCATGA |
| *stra6*-qR | GACGGTCGGATACTCCTGTG |
| *rara2a*-qF | TTCACCACCCTTACCATCGC |
| *rara2a*-qR | CGTTGTGCATCTGTGTTCGG |
| *rara2b*-qF | TTGTGGGACTGACCCCTAGC |
| *rara2b*-qR | CTGGGTCTCCACAGAGTGAT |
| *rarga*-qF | TGGACCCTTTTGCTTGGACC |
| *rarga*-qR | GCACCATCTCCTCCGAACTG |
| *rargb*-qF | TCGTGTGCCAGGACAAATCT |
| *rargb*-qR | GCGCGTCACTTTGTTGATTG |
| *fasn*-qF | GAGAAAGCTTGCCAAACAGG |
| *fasn*-qR | GAGGGTCTTGCAGGAGACAG |
| *scd*-qF | CACCACACGTTTCCCTACGA |
| *scd*-qR | CATGGTGTCCACGAAGATGG |
| *elovl2*-qF | CACTGGACGAAGTTGGTGAA |
| *elovl2*-qR | GTTGAGGACACACCACCAGA |
| *elovl6*-qF | CTATGCTCTTCGGGCAGCC |
| *elovl6*-qR | ACCATCTGGGTGATCTGTGTCA |
| *dgat1a*-qF | CTGGGCGTTTCTGGGTATGA |
| *dgat1a*-qR | GCGTTGCCATAGTTACCCCTC |
| *srebf1*-qF | CATCCACATGGCTCTGAGTG |
| *srebf1*-qR | CTCATCCACAAAGAAGCGGT |
| *srebf2*-qF | CACTCACACAAGACACACAG |
| *srebf2*-qR | ACCTGGTTCTGGATGAATCG |
| *apoa1a*-qF | ACTATGAGCAGTACAAACTCCAGC |
| *apoa1a*-qR | CGTAGGGGGTCAAAGCCTG |
| *apoa1b*-qF | CCAGCCACATCCACAGATCC |
| *apoa1b*-qR | TCGAGGTGAGTGAGGGACTT |
| *apoa4a*-qF | GTGGACCAGCATCTGCAGGAGTT |
| *apoa4a*-qR | CTTCAGCATAGGGTGTGAGACTC |
| *apoa4b*-qF | TACCAGTGGGACTTAAAGCAAACA |
| *apoa4b*-qR | TGTGAATGCCACAAAGGCAAG |
| *apoa2*-qF | TGGTGGTTTGGCCTCTGAAC |
| *apoa2*-qR | GGTACGCACCAACATAGGGT |
| *apoea*-qF | TCGGCAGAGAATTAGACACAC |
| *apoea*-qR | TCGTTGAACTTCTGGGCTCTC |
| *apoeb*-qF | TGACGTGAAGAACCGTGTCG |
| *apoeb*-qR | TAGGTTGCTACGGTGTTGCG |
| *apoba*-qF | CTCTTTGGAGAGCGCTTGGA |
| *apoba*-qR | AGCGTGGAACGAAGACCATT |
| *apobb.1*-qF | GTCGTTATGCTTTTGCACCA |
| *apobb.1*-qR | CGATGGCATTGTCAATCAAG |
| *lpl*-qF | GGAATACACGGCGAGAAGGAG |
| *lpl*-qR | TGTCGGAGTTCCACCAGGC |
| *lipc*-qF | CACTGGCAAAAGCAAGAGGC |
| *lipc*-qR | TCTCCTCGACAAGTGTTA TGGG |
